# Reference gene analysis and its use for kinase expression profiling in *Fasciola hepatica*

**DOI:** 10.1038/s41598-019-52416-x

**Published:** 2019-11-01

**Authors:** Hicham Houhou, Oliver Puckelwaldt, Christina Strube, Simone Haeberlein

**Affiliations:** 1 Institute of Parasitology, BFS, Justus Liebig University, Giessen, Germany; 2 Institute for Parasitology, Centre for Infection Medicine, University of Veterinary Medicine Hanover, Hanover, Germany

**Keywords:** Drug discovery, Molecular biology, Zoology, Diseases

## Abstract

The liver fluke *Fasciola hepatica* causes fasciolosis, a foodborne zoonosis affecting humans and livestock worldwide. A reliable quantification of gene expression in all parasite life stages relevant for targeting by anthelmintics in the mammalian host is fundamental. The aim of this study was to define a set of stably expressed reference genes for qRT-PCR in *Fasciola* studies. We determined the expression stabilities of eight candidate reference genes by the algorithms NormFinder, geNorm, BestKeeper, and comparative ΔCT method. The most stably expressed reference genes for the comparison of intra-mammalian life stages were glutamyl-prolyl-tRNA synthetase (Fh*eprs*) and tubulin-specific chaperone D (Fh*tbcd*). The two best reference genes for analysis of *in vitro*-cultured juveniles were Fh*tbcd* and proteasome subunit beta type-7 (Fh*psmb7*). These genes should replace the housekeeping gene *gapdh* which is used in most *Fasciola* studies to date, but in fact was differentially expressed in our analysis. Based on the new reference genes, we quantified expression of five kinases (Abl1, Abl2, PKC, Akt1, Plk1) discussed as targets in other parasitic flatworms. Distinct expression patterns throughout development were revealed and point to interesting biological functions. We like to motivate using this set of validated reference genes for future *F*. *hepatica* research, such as studies on drug targets or parasite development.

## Introduction

The liver fluke *Fasciola hepatica* is a cosmopolitan parasitic flatworm causing zoonotic disease in humans and tremendous economic losses by infecting livestock^1^. The life cycle of *F*. *hepatica* is complex and includes a snail as an intermediate host and a mammal as a definitive host. The fluke develops through multiple stages: from eggs to miracidia, sporocysts, rediae, cercariae, metacercariae, newly excysted juveniles (NEJs), and immature flukes which eventually reach the adult stage. Molecular research on this and other helminths has considerably advanced in recent years, with genome data and tools such as RNA interference (RNAi) becoming available for these complex multicellular organisms. Relative quantification of gene expression by quantitative real-time PCR (qRT-PCR) is an essential component of many experimental approaches. The accuracy of such relative gene expression analyses is largely dependent on the stable expression of the reference genes used for normalisation. Housekeeping genes are typically used as reference in qRT-PCR, although in some cases their expression is known to vary in helminths depending on the experimental condition, the parasite stage, or the parasite’s sex^2–4^. Classical housekeeping genes such as glyceraldehyde-3-phosphate dehydrogenase (*gapdh*) and *ß-actin* have often been used based on tradition rather than being experimentally validated as stably expressed genes for the species or parasite stage of interest. However, the validation of expression stabilities of reference genes under the desired experimental conditions is an essential step, which should precede any comparative studies on expression levels of target genes.

Comparative gene expression analysis in different life-stages is of major interest in *F*. *hepatica* research. This includes for instance the validation of expression of potential drug target genes. A new active compound should ideally target all life stages within the final host, including NEJs emerging from metacercariae in the host’s intestine, immature flukes migrating through the body cavity to the liver capsule and through the liver parenchyma causing acute fascioliasis, and adult flukes, which trigger chronic fascioliasis while residing inside the bile ducts where egg deposition occurs^5^. Gene expression often varies between intra-mammalian life stages of *F*. *hepatica* as metabolic, nutritional and locomotor demands vary between stages^6,7^. Other research approaches include the *in vitro* culture of flukes, such as the recently established *in vitro* maturation of NEJs to immature flukes in order to study early development^8^, or RNAi-mediated knockdown of gene expression which might involve culture for more than 3 weeks^9^. Gene expression stability in parasites during *in vitro* culture is likely to differ from non-cultured parasites, because of the known differences from the *in vitro* conditions compared to the natural environment provided by the host. This motivated us to identify reference genes with stable expression in the intra-mammalian life stages and during *in vitro* culture of *F*. *hepatica*. To this end, we used four different algorithms to assess eight different candidate reference genes and their expression stability in the relevant *in vivo*-stages (NEJs, 4 week-old immature flukes, 12-week old adults), and in juvenile flukes cultured for 4 weeks *in vitro*. Out of these candidate genes, three were identified as the most stably expressed reference genes.

As a first application example, we utilised these selected reference genes to determine the expression level of potentially druggable target genes in the different life stages and during *in vitro* culture. Kinases are discussed as promising drug targets in parasitic flatworms^10,11^. For instance, promising anthelmintic effects were observed in immature and adult schistosomes by RNAi or inhibitor treatment against Abelson (Abl) tyrosine kinases and polo-like kinases (PLK)^12–15^. Surprisingly, in *Fasciola* research, kinases as drug targets have been largely neglected so far although new anthelmintics are urgently needed facing the spread of triclabendazole resistance around the globe^1,16,17^. We have identified five kinase orthologs in *F*. *hepatica* and characterised their expression by qRT-PCR. Based on the new reference genes, an interesting change in kinase expression patterns during fluke development was obtained. This may substantiate further research activity on kinases in *F*. *hepatica*.

## Results

### Selection of candidate reference genes

**Figure 1:**
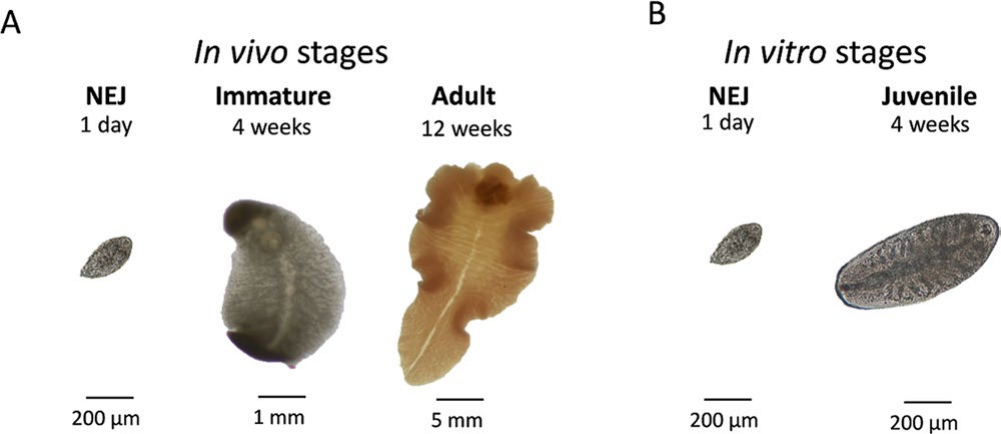
*In vivo* and *in vitro* stages of *F*. *hepatica* under investigation. Stably expressed reference genes were identified for the comparison of gene expression of (**A**) newly excysted juveniles (NEJs), immature and adult worms, i.e. stages relevant for the mammalian host; and (**B**) NEJs and *in vitro*-cultured juvenile worms, which are frequently studied as part of drug testing or knockdown of gene expression.

Eight different candidate reference genes were chosen among which we assumed to find stably expressed reference genes suitable for the quantification of gene expression independent from the parasite stage or *in vitro* cultivation (Fig. 1). The candidates were selected based on already available transcriptome datasets or literature on related trematode species. The candidate reference gene names, biological function and accession numbers are listed in Table 1. In detail, orthologs of two of the genes were previously shown to be stably expressed in selected life stages of the Chinese liver fluke *Clonorchis sinensis*^2^: β-actin (*actb*) and small nuclear ribonucleoprotein (*snrpa1*). Another two candidate genes were chosen because previous transcriptome analyses suggested a stable expression of their orthologs in all life stages of the blood fluke *Schistosoma mansoni*^18^: tubulin-specific chaperone D (*tbcd*) and protein phosphatase 1 catalytic subunit beta (*ppp1cb*). Two candidates were selected because we found them to be most stably expressed during *in vitro* culture of *S*. *mansoni*^4^: leucine zipper and EF-hand containing transmembrane protein 1 (*letm1*) and proteasome subunit beta type-7 (*psmb7*). The ortholog of a glutamyl-prolyl-tRNA synthetase (*eprs*) was included based on its stable expression in different strains of *F*. *hepatica*^19^. Finally, for comparison, we included the well-known housekeeping gene *gapdh*, which is currently widely used for normalisation also in *F*. *hepatica* studies^9,20,21^. Thus, all reference gene candidates play roles in conserved cellular processes like mRNA splicing and translation, cytoskeleton arrangement, glycolysis, mitochondrial organisation, and the regulation of cell growth (Table 1). Orthologs for all eight genes were identified by BLASTp search of known genes in *Homo sapiens* (accession numbers see Table 1) against the genome of *F*. *hepatica*. The presence of relevant conserved protein domains was confirmed by SMART analysis (see Supplementary Fig. S1). The identity of the orthologs was further confirmed by multiple alignment of the amino acid sequences with those of model species (see Supplementary Table S1 and Fig. S8).

**Table 1:** Overview of candidate reference genes for the study of gene expression in *F*. *hepatica*.

Gene name	Gene ID	Homology (e-value)*	Protein function
Fh*tbcd*	maker-scaffold10x_815_pilon-snap-gene-1.87	Tubulin-specific chaperone D [*H*. *sapiens*, NP_005984.3] (7e-177)	Cofactor D is one of four proteins involved in the pathway leading to correctly folded beta-tubulin from folding intermediates.
Fh*eprs*	maker-scaffold10x_14_pilon-snap-gene-0.109	Glutamyl-prolyl-tRNA synthetase [*H*. *sapiens*, NP_004437.2] (0.0)	The protein encoded by this gene is a multifunctional aminoacyl-tRNA synthetase that catalyses the aminoacylation of glutamic acid and proline tRNA species.
Fh*letm1*	maker-scaffold10x_721_pilon-snap-gene-0.10	Leucine zipper and EF-hand containing transmembrane protein 1 [*Homo sapiens*, NP_036450.1] (2e-85)	The protein functions to maintain the mitochondrial tubular shapes and is required for normal mitochondrial morphology and cellular viability.
Fh*actb*	augustus_masked-scaffold10x_269_pilon-processed-gene-0.18	Actin, cytoplasmic 1 [*H*. *sapiens*, NP_001092.1] (0.0)	Actins are highly conserved proteins that are involved in cell motility, structure, integrity and intracellular signaling. The encoded protein is a major constituent of the contractile apparatus and one of the two non-muscle cytoskeletal actins that are ubiquitously expressed.
Fh*snrpa1*	maker-scaffold10x_234_pilon-snap-gene-0.20	U2 small nuclear ribonucleoprotein A’ [*H*. *sapiens*, NP_003081.2] (5e-72)	This gene encodes a protein which is a component of the spliceosome and it is involved in pre-mRNA splicing.
Fh*ppp1cb*	maker-scaffold10x_238_pilon-snap-gene-0.95	Protein phosphatase 1 catalytic subunit beta [*H*. *sapiens*, NP_002700.1] (0.0)	The protein encoded by this gene is one of the three catalytic subunits of protein phosphatase 1 (PP1). PP1 is a serine/threonine specific protein phosphatase known to be involved in the regulation of a variety of cellular processes.
Fh*psmb7*	maker-scaffold10x_1452_pilon-augustus-gene-0.11	Proteasome subunit beta type-7 [*H*. *sapiens*, NP_002790.1] (1e-79)	Important component of the cellular protein degradation complex.
Fh*gapdh*	maker-scaffold10x_2706_pilon-snap-gene-0.15	Glyceraldehyde-3-phosphate dehydrogenase [*H*. *sapiens*, NP_001276674.1] (0.0)	GAPDH catalyzes the sixth step of the glycolysis by converting D-glyceraldehyde 3-phosphate to 3-phospho-D-glyceroyl phosphate.

^*^Determined by NCBI BLAST.

### RNA quality of parasite samples and performance of qRT-PCR primers

**Figure 2:**
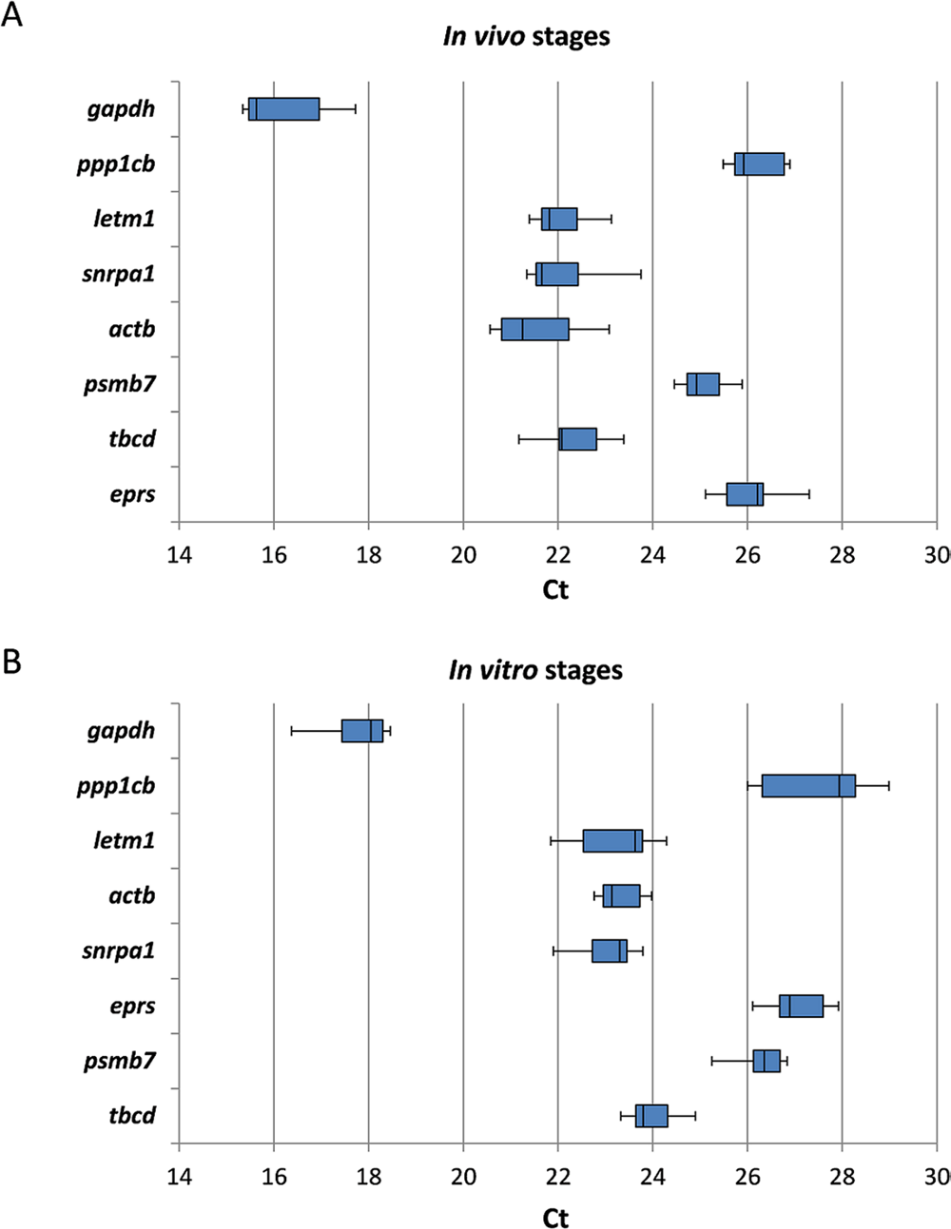
Distribution of threshold cycle (Ct) values of 8 candidate reference genes across all samples. The solid line represents the median, boxes indicate the 25^th^ and 75^th^ percentile, and the whiskers represent the minimum and maximum values of averaged qRT-PCR expression data from (**A**) 9 samples of intra-mammalian *in vivo* stages (NEJs, immature and adult flukes), and (**B**) 6 samples of *in vitro* cultured juveniles (NEJs and 4-week cultured juveniles).

Isolation of sufficient amounts of high-quality RNA from low numbers of NEJs is often problematic because of their small size. Using the Monarch RNA Extraction Kit, we managed to isolate and analyse RNA from as little as 10 NEJs. Representative electropherograms from BioAnalyzer analysis of RNA quantity and quality are shown in Supplementary Fig. S2. From 10 and 20 NEJs, we obtained in average 4–6 ng and 9–14 ng RNA, respectively. A good RNA integrity was reflected by the distinct 18 S RNA peak. Primer specificity was confirmed by PCR yielding one specific amplification product of expected size (see Supplementary Table S2) for each candidate reference gene, without primer-dimer formation or genomic DNA contamination. The primer sequences and amplicon lengths can be found in Supplementary Table S2. Absence of unspecific products was also confirmed by melt-curve analysis, which showed a single peak. The expression level of the eight candidate reference genes was assessed by qRT-PCR by determining the Ct value for each sample. With average Ct values of 15.27 to 27.63, all candidate genes were within the range of an acceptable reference gene expression level (15 < Ct < 30) (Fig. 2). The highest expression was found for Fh*gapdh*, and the lowest for Fh*ppp1cb*.

### Identification of the most stably expressed reference genes

Expression stabilities of the eight candidate reference genes were determined by four different algorithms: NormFinder, geNorm, BestKeeper, and the comparative ΔCt method. As input data, we used expression values either of different intra-mammalian stages (NEJs, 4 week-old immature flukes, 12 week-old adults), or of NEJs prior and after 4 weeks of *in vitro*-culture (then called 4 week-old juveniles) (Fig. 1). Transcript levels for all samples were determined by absolute quantification against a standard curve. Input data for stability analysis were raw Ct values (BestKeeper), relative Ct values (geNorm, ΔCt method), and calculated concentrations of amplification products (NormFinder), respectively. The most stably expressed reference gene has a low stability value (NormFinder, geNorm, ΔCt method), or a high coefficient of correlation (BestKeeper).

#### Expression stability of candidate reference genes in different fluke stages

**Figure 3:**
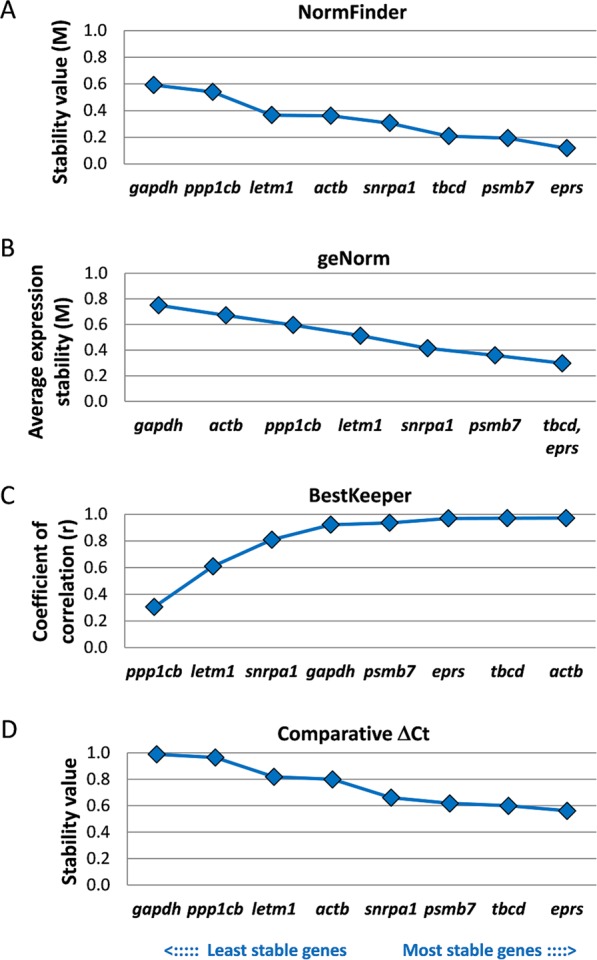
Stability of expression of eight candidate reference genes in three different intra-mammalian life stages of *F*. *hepatica*. Stability values were obtained for NEJs, 4 week-old immature, and 12 week-old adult worms using NormFinder (**A**), geNorm (**B**), BestKeeper (**C**), and the comparative ΔCT method (**D**). Genes were ranked from the least stable (on the left) to the most stable (on the right).

In a first step, the expression stabilities of the selected candidate genes between three different fluke stages relevant for the final host were determined. The obtained ranking of each algorithm is summarised in Fig. 3 and Supplementary Table S3.

NormFinder ranks genes according to their stability value M, which is based on the size of intra- and inter-group expression variations, i.e. the variation within an experimental group (here: biological replicates) and between different experimental groups (here: fluke stages). A good reference gene is characterised by an M value below 1 in heterogeneous cell or tissue sample sets (Vandesompele 2002). NormFinder identified the tRNA-synthetase Fh*eprs* and the proteasome subunit Fh*psmb7* as the two best candidates, whereas Fh*gapdh* turned out to be the least stably transcribed gene in a combined analysis of all fluke stages (Fig. 3A). Additionally, expression stabilities of the eight reference gene candidates were calculated using the geNorm algorithm and ranked from the most stable to least stable candidate gene (Fig. 3B). geNorm analysis ranked the tubulin chaperone Fh*tbcd* and Fh*eprs* as the two best reference genes for fluke stage comparison and, again, Fh*gapdh* as least suitable candidate. In BestKeeper analysis, the average of the pairwise variations of each gene with all other genes is used to create the stability value M: the lower M is, the more stable are the transcript levels of a gene. The calculated standard deviation [±CP] should not exceed 1. Indeed, standard deviations of all eight genes were <1. BestKeeper identified Fh*tbcd* and the actin Fh*actb* as the best reference genes, and the protein phosphatase subunit Fh*ppp1cb* as worst candidate (Fig. 3C). The fourth algorithm, the ΔCt method, revealed Fh*eprs* and Fh*tbcd* as the best candidates and Fh*gapdh* as the least stably expressed gene (Fig. 3D). These results were equivalent to those generated by geNorm. For some genes, such as Fh*actb* and Fh*gapdh*, the predicted expression stability (here: coefficient of correlation) according to BestKeeper was fairly good, whereas these genes were among the least or only average stably expressed genes in the three other analyses. This argues for using more than just one algorithm when performing studies of reference gene validation.

Because of the slightly heterogeneous rankings produced by the four algorithms, a final global ranking was obtained by assigning the numbers 1–8 to each stability coefficient (with 1 as the most stable and 8 as the least stable gene), and creating the geometric mean of these ranks for each gene. This procedure is equivalent to the global ranking done by the RefFinder tool^22^. This final ranking revealed Fh*eprs* and Fh*tbcd* as the two most stably expressed genes and, therefore, the most suitable reference genes for comparisons of intra-mammalian fluke stages. The commonly used housekeeping gene *gapdh* ranked last. The final ranking from the lowest to highest calculated average rank was as follows: Fh*eprs* < Fh*tbcd* < Fh*psmb7* < Fh*actb* < Fh*snrpa1* < Fh*letm1* < Fh*ppp1cb* < Fh*gapdh* (Fig. 5A).

#### Expression stability of candidate reference genes during *in vitro* culture of juvenile flukes

**Figure 4:**
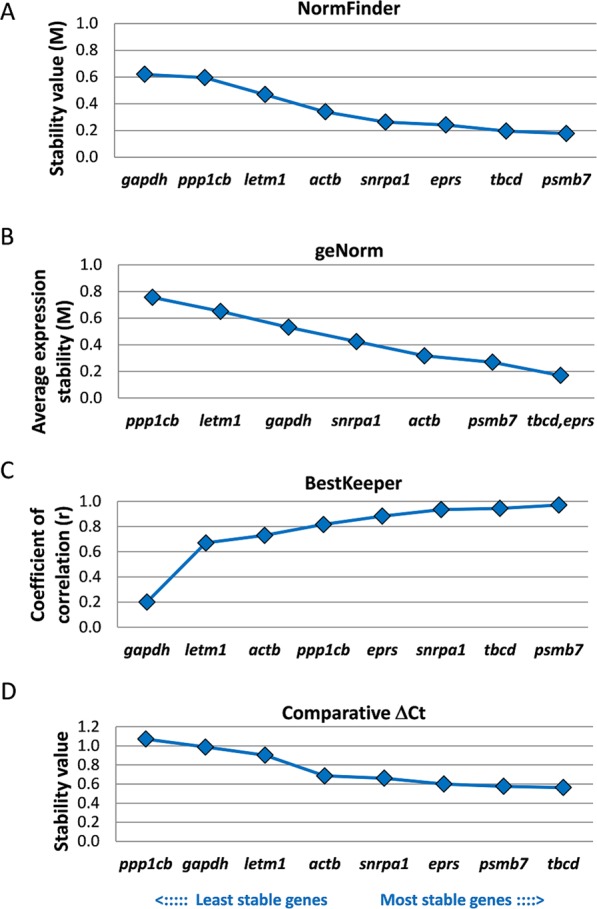
Stability of expression of eight candidate reference genes during *in vitro* culture of juvenile *F*. *hepatica*. Stability values were obtained for 1-day old NEJs and juveniles grown for 4 weeks in serum-rich medium using NormFinder (**A**), geNorm (**B**), BestKeeper (**C**), and the comparative ΔCT method (**D**). Genes were ranked from the least stable (on the left) to the most stable (on the right).

Next to quantification of gene expression in different fluke stages, gene expression analyses in *in vitro*-cultured NEJs is another standard approach for various research questions such as expression changes during maturation of juveniles or characterisation of gene function by RNAi^9,20,23^. Analogous to the stability ranking for the intra-mammalian stages, we used the four different algorithms to identify the most stably expressed reference genes for NEJs before and after 28 days of *in vitro* culture in an established long-term culture medium^8^. The individual ranking for each algorithm is summarised in Fig. 4 and Supplementary Table S4.

NormFinder, BestKeeper, and the comparative ΔCT method identified Fh*tbcd* and Fh*psmb7* as the two most stably expressed genes. geNorm identified Fh*tbcd* and Fh*eprs* as most suitable reference genes, as for the analysis of intra-mammalian stages before, followed by Fh*psmb7*. The least stably expressed gene was Fh*ppp1cb* (geNorm and ΔCT method) or Fh*gapdh* (NormFinder and BestKeeper).

**Figure 5:**
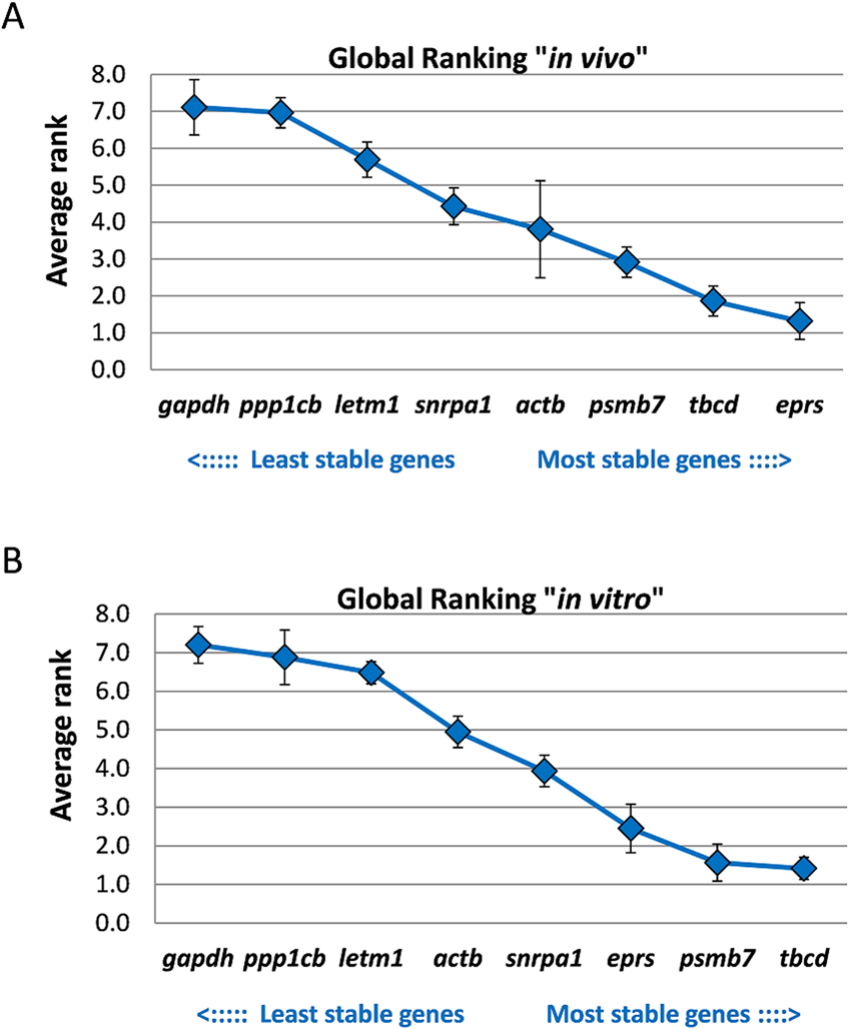
Mean rank of expression stability of eight candidate reference genes in *F*. *hepatica*. A number (from 1 to 8) was assigned to each stability coefficient obtained from four algorithms. The mean rank with SEM is shown for (**A**) the analysis of three intra-mammalian stages (NEJs, immature and adult flukes), and (**B**) the analysis of *in vitro*-cultured juveniles (NEJs before and after 28 days culture). Genes were ranked from the least stable (on the left) to the most stable (on the right).

Accordingly, the global ranking based on the geometric mean of individual ranks revealed Fh*tbcd* and Fh*psmb7* as the most stably expressed reference genes. The average calculated ranks were as follows: Fh*tbcd* < Fh*psmb7* < Fh*eprs* < Fh*snrpa1* < Fh*actb* < Fh*letm1* < Fh*ppp1cb* < Fh*gapdh* (Fig. 5B).

### Relative expression levels of reference gene candidates during development and anthelmintic treatment

**Figure 6:**
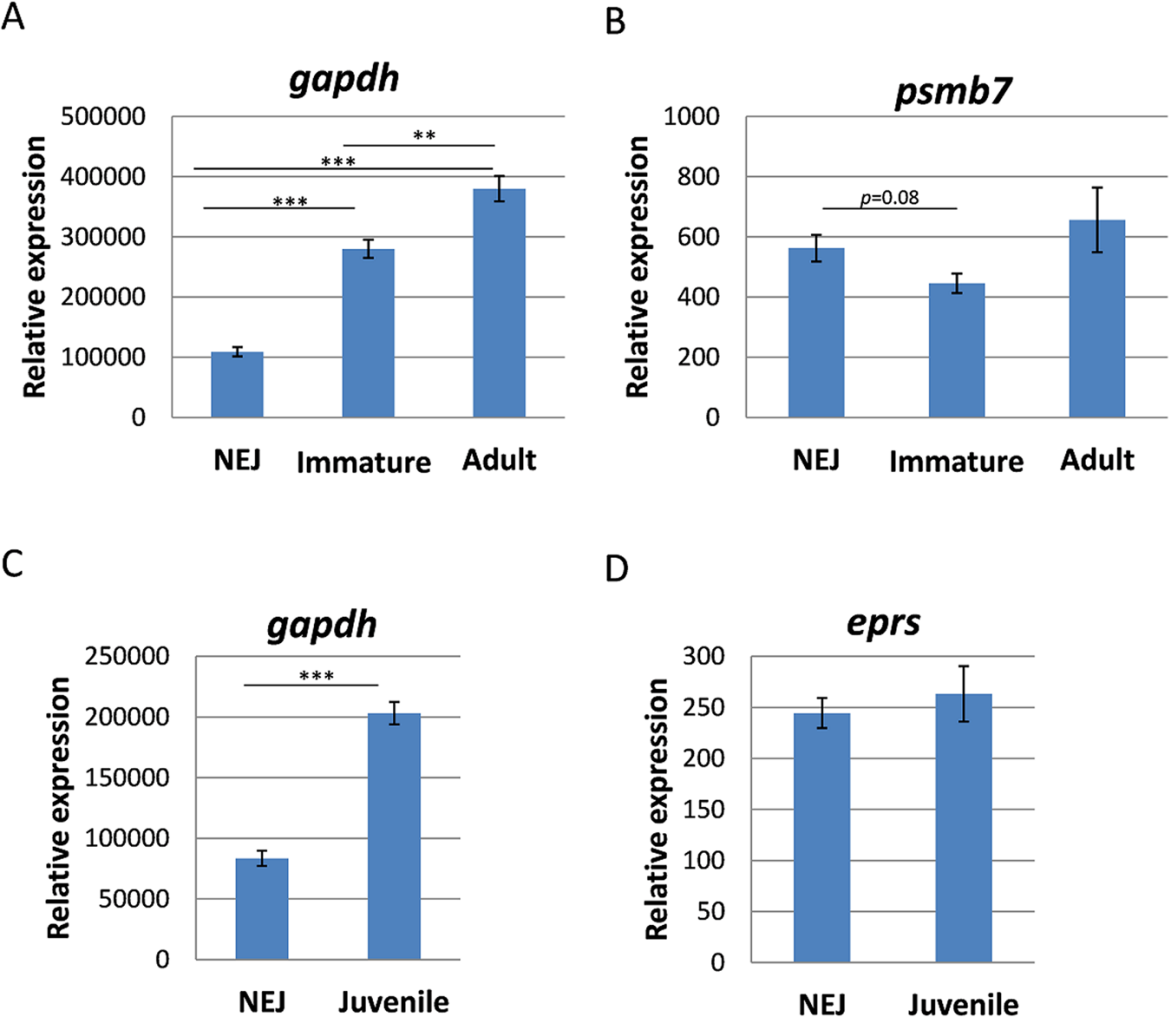
Relative expression levels of a stable and the least stable reference gene candidate in different stages of *F*. *hepatica*. Relative quantification is based on normalisation against the geometric mean of the two most stably expressed reference genes identified for (**A**,**B**) intra-mammalian stages (*tbcd* and *eprs*), or for (**C**,**D**) *in vitro* cultured juveniles (*tbcd* and *psmb7*). *gapdh* (**A**,**C**) was previously revealed as the least stable gene, *psmb7* and *eprs* (**B**,**D**) were among the three most stably expressed genes. Average values of 3–4 biological replicates with SEM are shown. Significant differences are indicated with **p < 0.01, ***p < 0.001 (t-test).

To clarify in how far the previously used reference gene Fh*gapdh*^9,20,21^ is differentially regulated during *in vivo* development or *in vitro* culture, we relatively quantified its expression compared to the geometric mean of the two best reference genes. Fh*gapdh* was clearly differentially expressed with a significant upregulation during *in vitro* culture and during development from NEJs to immature and adult flukes (Fig. 6A,C). A differential expression was also found for most other reference gene candidates positioned on number 4 to 7 in the global ranking (see Supplementary Fig. S3). On the contrary, Fh*psmb7* and Fh*eprs* were not differentially expressed, being in line with their fairly good stability rank (number 3 in the global rankings) (Fig. 6B,D). Taken together, Fh*gapdh* as well as all genes from global stability rank 4 and higher appear not to be suitable as reference genes for inter-stage comparisons.

**Figure 7:**
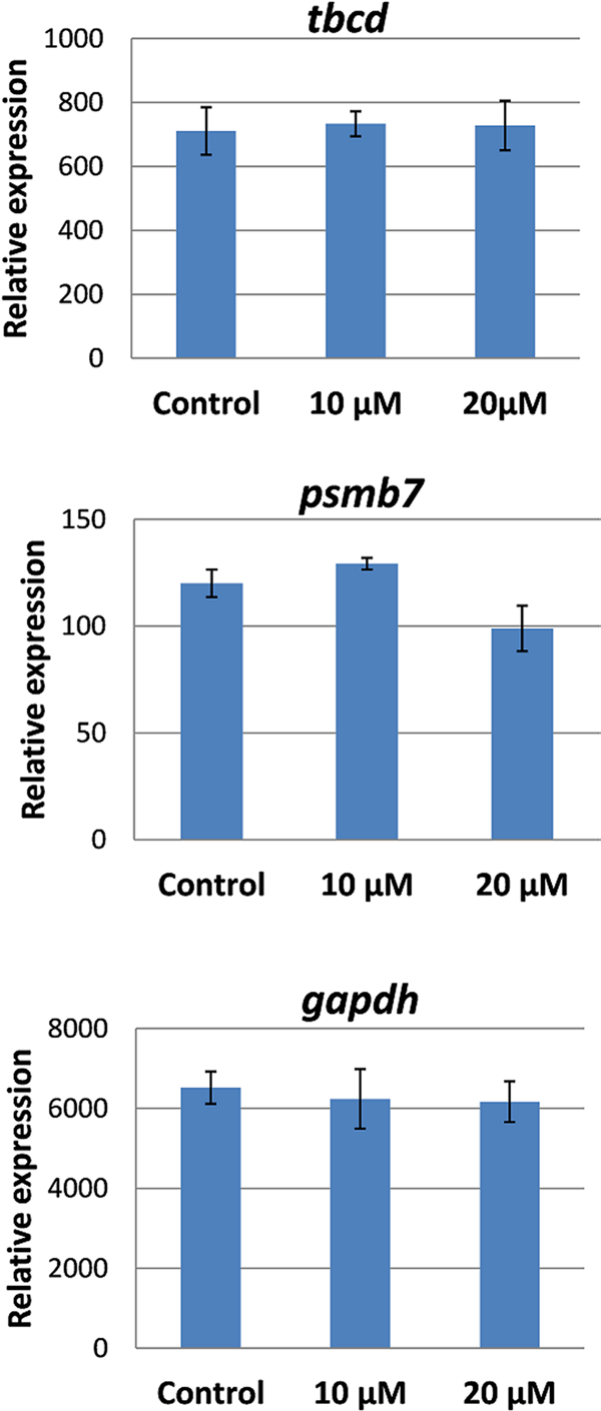
Relative expression of selected reference genes after culture with triclabendazole. Adult *F*. *hepatica* were cultured for 2 days with 10 µM and 20 µM triclabendazole, or in an equivalent concentration of DMSO as control. Expression of *tbcd* (**A**) was normalised against the geometric mean of *eprs* and *psmb7*, expression of *psmb7* (**B**) and *gapdh* (**C**) were normalised against the geometric mean of *eprs* and *tbcd*. Average values of 4 biological replicates with SEM are shown.

Next, we addressed whether selected reference genes are also stably expressed during anthelmintic treatment of flukes. To this end, adult *F*. *hepatica* were cultured with different sublethal concentrations of triclabendazole for 2 days *in vitro*. Because triclabendazole is known to affect tubulin in *F*. *hepatica*^24^, it was of particular interest whether the tubulin-specific chaperone D Fh*tbcd* would be stably expressed. The expression of all genes investigated (Fh*tbcd*, Fh*psmb7*, Fh*gapdh*) did not change by drug exposure (Fig. 7) and thus appear suitable as reference genes for this type of experimental setting.

### Validation of selected reference genes for quantification of target gene expression in different fluke stages

The performance of the selected reference genes was validated by quantification of relative expression levels of five orthologs of kinase genes in *F*. *hepatica*. Kinases are discussed as promising anthelmintic target of inhibitors^10,25^. In particular, Abl kinases and polo-like kinase 1 have been studied in the past as potential targets in schistosomes, tape-worms, and filariae^12,26–28^. Furthermore, the protein kinases B (also called Akt) and C have been investigated as potential targets in *S*. *mansoni*^13,29,30^. For all these kinases, RNAi or inhibitor treatment *in vitro* revealed anthelmintic effects on immature or adult schistosome stages.

All the more surprising is that neither of these kinases has been studied to date in *Fasciola*. Knowledge on kinase expression in all life stages relevant for drug targeting is desirable, as well as a reliable quantification of expression during *in vitro* culture, for instance as part of knockdown experiments. Therefore, we identified orthologs of above mentioned kinases by BLASTp search (Table 2): Fh*abl1* and Fh*abl2* as orthologs of the protein tyrosine kinases *abl1* and *abl2*, which play roles in a variety of cellular processes including cell differentiation and cytoskeletal rearrangements^31^; Fh*akt1* and Fh*pkc* as orthologs of the serine/threonine-protein kinases B and C, which are known to regulate many processes including cell metabolism, proliferation, and survival^32,33^; Fh*plk1* as an ortholog of polo-like kinase 1 with important roles during cell cycle progression^34^. The presence of conserved protein domains was confirmed by SMART analysis (see Supplementary Fig. S4). For instance, Fh*plk1* contained a typical kinase domain and two polo-box domains. The identity of the kinase orthologs was further confirmed by multiple alignment of the amino acid sequences against several model species (see Supplementary Fig. S9).

**Table 2:** Overview of genes of interest for the study of gene expression in *F*. *hepatica*.

Gene of interest	Gene ID	Homology (e-value)^*^	Protein function
Fh*abl1*	maker-scaffold10x_1995_pilon-snap-gene-0.46	ABL proto-oncogene 1, non-receptor tyrosine kinase [*H*. *sapiens*, NP_005148.2] (2e-119)	Protein tyrosine kinase involved in a variety of cellular processes, including cell division, adhesion, differentiation, and response to stress.
Fh*abl2*	maker-scaffold10x_873_pilon-snap-gene-0.69	ABL proto-oncogene 2, non-receptor tyrosine kinase isoform e [*H*. *sapiens*, NP_001129473.1] (2e-135)	Protein tyrosine kinase with a role in cytoskeletal rearrangements through its F-actin- and microtubule-binding sequences.
Fh*akt1*	maker-scaffold10x_205_pilon-augustus-gene-0.40	Rac-alpha serine/threonine-protein kinase [*H*. *sapiens*, NP_001014431.1] (9e-137)	The serine/threonine-protein kinase AKT1 is also known as protein kinase B. AKT kinases regulate many processes including metabolism, proliferation, cell survival, and growth.
Fh*pkc*	maker-scaffold10x_608_pilon-snap-gene-0.5	Protein kinase C iota [*H*. *sapiens*, NP_002731.4] (0.0)	A serine/threonine protein kinase involved in cell survival, differentiation and polarity. It plays a role in microtubule dynamics in the early secretory pathway.
Fh*plk1*	maker-scaffold10x_784_pilon-snap-gene-0.36	Polo-like kinase 1 [*H*. *sapiens*, NP_005021.2] (0.0)	The serine/threonine protein kinase is highly expressed during mitosis and performs several important functions throughout the M phase of the cell cycle.

^*^Determined by NCBI BLAST.

**Figure 8:**
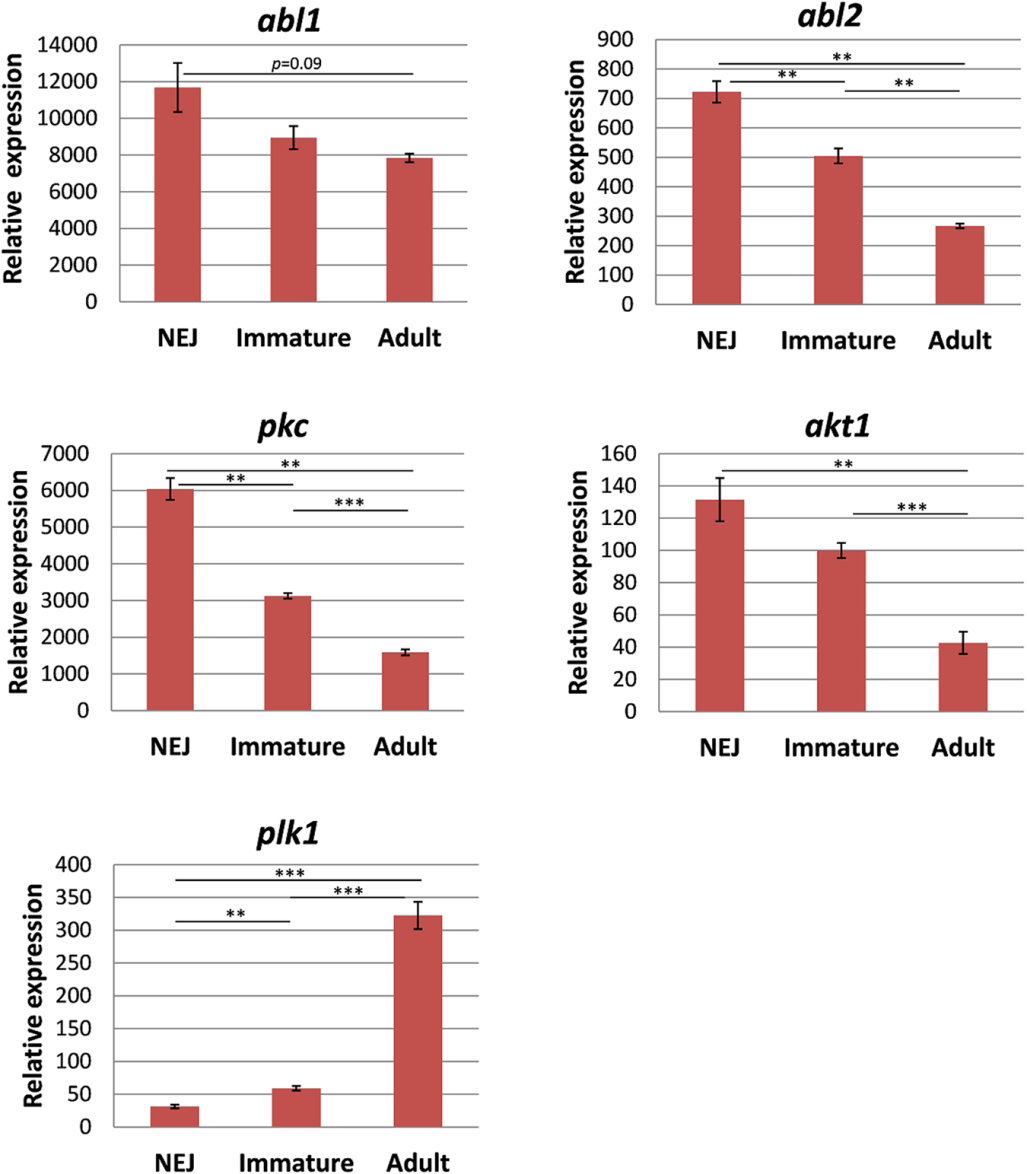
Relative expression levels of kinases in three different intra-mammalian life stages of *F*. *hepatica*. Expression data from NEJs, 4 week-old immature, and 12 week-old adult worms were normalised against the geometric mean of the two most stably expressed reference genes (*eprs* and *tbcd*). Average values of 3–4 biological replicates with SEM are shown. Significant differences are indicated with **p < 0.01, ***p < 0.001 (t-test). *abl1*, tyrosine-protein kinase Abl1; *abl2*, tyrosine-protein kinase Abl2; *pkc*, protein kinase C; *akt1*, Rac-alpha serine/threonine-protein kinase 1; *plk1*, polo-like kinase 1

**Figure 9:**
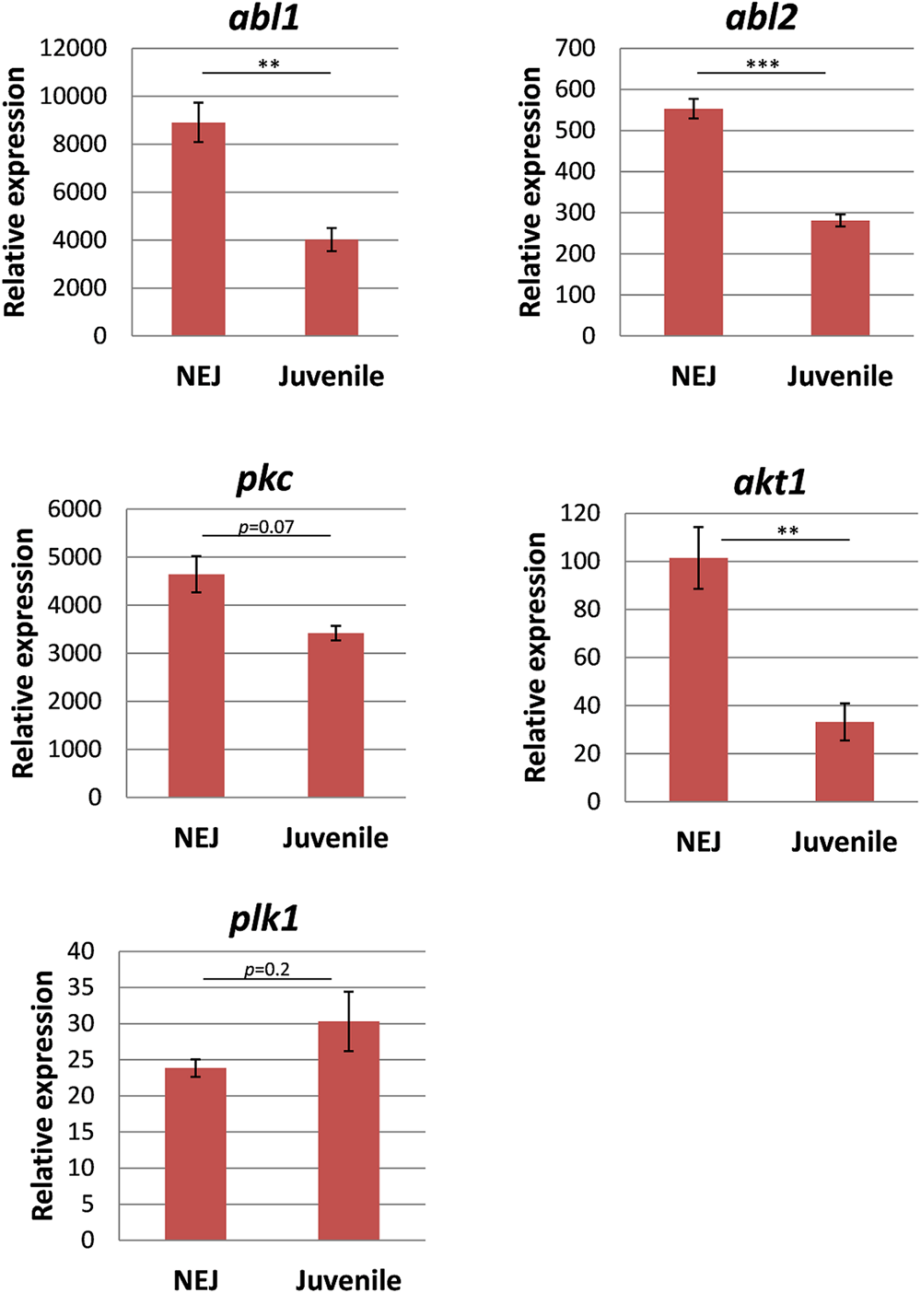
Relative expression levels of kinases during *in vitro* culture of juvenile *F*. *hepatica*. Expression data from NEJs and juvenile worms grown for 4 weeks in serum-rich medium were normalised against the geometric mean of the two most stably expressed reference genes (*tbcd* and *psmb7*). Average values of 3–4 biological replicates with SEM are shown. Significant differences are indicated with **p < 0.01, ***p < 0.001 (t-test). *abl1*, tyrosine-protein kinase Abl1; *abl2*, tyrosine-protein kinase Abl2; *pkc*, protein kinase C; *akt1*, Rac-alpha serine/threonine-protein kinase 1; *plk1*, polo-like kinase 1.

For both experimental groups (*in vivo* and *in vitro* stages), we quantified kinase expression using the geometric mean of the two most stably expressed reference genes for normalisation, which were Fh*eprs* and Fh*tbcd*, and Fh*psmb7* and Fh*tbcd*, respectively. The five kinase genes were found to be expressed in all intra-mammalian life stages. The highest relative expression was observed for Fh*abl1* and Fh*pkc*. Interestingly, two types of expression patterns during fluke development were revealed: while Fh*plk1* was expressed highest in adult flukes and low in NEJs and immature flukes, expression of all other kinases was highest in NEJs and significantly downregulated during development to immature and adult flukes (Fig. 8). *In vitro* maturation of NEJs to immature flukes by 4-week culture revealed very similar expression patterns (Fig. 9) as seen during *in vivo* development.

As a proof of principle, we also used Fh*gapdh* for normalisation of kinase gene expressions to assess whether using a suboptimal gene would yield different results compared to using the top ranked reference genes. While the overall kinase expression patterns were largely similar, a differing expression pattern was revealed for some kinase genes and some stages. As example, Fh*gapdh* suggested a significant downregulation of Fh*abl1* and Fh*akt1* expression from NEJs to immature flukes (Supplementary Fig. S5), while there was no expression difference based on the stable expressed reference genes (Fig. 8). On the other hand, the significantly upregulated expression of Fh*plk1* from NEJs to immature flukes was not evident when normalised against Fh*gapdh*. Even more striking, Fh*gapdh* suggested a significant downregulation of Fh*plk1* during *in vitro* culture of juveniles (Supplementary Fig. S6), while in fact a trend for increased expression was demonstrated before (Fig. 9). This clearly shows that using a suboptimal reference gene for normalisation might give differing or even opposite expression results for a gene of interest.

The developmental expression changes for kinase genes found in *F*. *hepatica* matched in parts with expression patterns of orthologs in the related blood fluke *S*. *mansoni*. Previous studies showed that the schistosome *abl* kinase genes Sm*abl1* and Sm*abl2* were downregulated during maturation from schistosomula to adults, and *plk1* was strongly upregulated, at least in adult females^3,18^ (Supplementary Fig. S7). Taken together, various kinases are expressed in intra-mammalian life stages of *F*. *hepatica*, and expression changes during *in vivo* development are mimicked by *in vitro* culture.

## Discussion

Proteomic studies revealed striking differences of gene expression among the life stages of *F*. *hepatica* occurring in the mammalian host^6,7^. This has important practical implication for vaccine development and drug target research. Knowledge on target gene expression in all life stages relevant for anthelmintics development is desirable as well as reliable methods for the quantification of gene expression during *in vitro* culture, for instance as part of target gene validation using knockdown experiments.

Per definition, housekeeping genes are essential for maintaining the cellular function and therefore, in theory, should be stably expressed. In practice, however, they may turn out to be regulated to some extent depending on the organisms, developmental stages, tissue types, and experimental settings^35^. This requires an accurate validation of candidate genes as reference genes for e.g. qRT-PCR studies. As classical housekeeping genes, *gapdh* and *ß-actin* have been used for normalisation in gene expression studies in all kind of organisms, including helminths^9,20,21,36^. At least for the oriental liver fluke, *Clonorchis sinensis*, a stable expression of *ß-actin* was demonstrated in the comparison of two life stages, metacercariae and adults. In the same study, however, *gapdh* exhibited poor expression stability^2^. Furthermore, a *gapdh* ortholog was among the least stable candidate genes for gene expression studies in adult schistosomes cultured *in vitro*^4^. In our study with *F*. *hepatica*, *gapdh* showed the lowest and *ß-actin* only a moderate expression stability among eight candidate genes tested. The transcript levels of *gapdh* appeared to be significantly upregulated during maturation of the fluke. This is of particular relevance as *gapdh* is currently a standard gene used for normalisation of gene expression in *F*. *hepatica* studies^9,20,21^, but based on our results, *gapdh* may not be the most suitable reference gene for inter-stage comparison of gene expression by qRT-PCR. This implies that without additional validation, the selection of reference genes for gene expression studies in one species should not be based on results obtained in a related species because there is no guarantee for comparable expression profiles.

We aimed at identifying stably expressed genes among a selection of eight candidate reference genes for two different experimental settings often used in *Fasciola* research: the comparison of life stages relevant for the mammalian host, and the *in vitro* culture of juvenile flukes. The four algorithms resulted in a slightly divergent ranking of the genes, which was expected from previous studies on other organisms, and which can be explained by the different type of input data and data processing used by the algorithms^4,37^. Therefore, a global ranking based on the results of all algorithms was performed. In the three intra-mammalian stages, the glutamyl-prolyl-tRNA synthetase Fh*eprs* and tubulin-specific chaperone D Fh*tbcd* were most stably expressed. In cultured parasites, this applied to Fh*tbcd* and the proteasome subunit beta type-7 Fh*psmb7*.

The tubulin-specific chaperone TBCD is one of four proteins involved in the pathway leading to correctly folded beta-tubulin from folding intermediates. Being involved in the regulation of microtubule polymerisation and depolymerisation^38,39^, it is required for crucial cellular processes such as proper assembly of the mitotic spindle and correct progression of mitosis. The glutamyl-prolyl-tRNA synthetase EPRS belongs to the family of aminoacyl-tRNA synthetases, which charge tRNAs with their corresponding amino acids. Accordingly, EPRS catalyses the aminoacylation of proline and glutamic acid tRNA species^40^. The proteasome subunit beta type-7 PSMB7 is part of the 20S and 26S proteasome complexes and thus involved in the proteolytic degradation of most intracellular proteins. The proteasome plays a key role in the maintenance of protein homeostasis by removing unneeded proteins, and damaged or misfolded proteins that could impair cellular functions^41^. Because microtubule function, tRNA synthesis, and proteolytic degradation are essential processes for all cells, and presumably independent of any developmental stage, it was not surprising that *tbcd*, *eprs* and *psmb7* turned out as the most stably expressed genes in our study.

The stability ranking obtained for *in vitro*-cultured liver flukes is in part similar to the ranking of a related study in *S*. *mansoni*. Here the ortholog for proteasome subunit beta type-7, Sm*psmb7*, ranked also second best during *in vitro* culture of adult worms, and Sm*gapdh* was among the least stably expressed genes^4^. In contrast, the most stable gene in adult schistosomes, *letm1*, was among the least stable genes investigated in *Fasciola*. Heterogenous rankings were also obtained for *tbcd* and *ppp1cb*: both genes were revealed to be stably expressed in the different life stages of *S*. *mansoni* by a meta-analysis study^18^, while in *F*. *hepatica*, only *tbcd* was. Taken together, the most stably expressed reference genes among the tested candidates in *F*. *hepatica* are housekeeping genes belonging to the family of tRNA synthetases, proteasome subunits, and the microtubule machinery.

For a first application of the newly identified reference genes in qRT-PCR experiments, we focused on kinase genes since they are discussed as potential druggable targets in various helminth species^10,26,27^. Surprisingly, kinases as drug targets have been largely neglected so far in *Fasciola* research. In the past, selected kinases have been in focus mainly as vaccine candidate (phosphoglycerate kinase) or as marker gene for discriminating hybrids of *Fasciola spp*. (phosphoenolpyruvate carboxykinase)^42,43^. Phosphofructokinase seems to be the only kinase of *F*. *hepatica* studied as potential chemotherapeutic target, in work by Mansour dating back as far as 1962^44,45^, but was not further followed because of suboptimal *in vivo* efficacy of an phosphofructokinase inhibitor^46^. To move kinases more into the spotlight of *Fasciola* anthelmintics research, we identified five kinase genes in *F*. *hepatica* and quantified their expression during development at the transcriptional level, *in vivo* and *in vitro*. For orthologs of all these kinases, promising anthelmintic effects have been obtained *in vitro* by knockdown of kinase gene expression or kinase inhibitor treatment in other parasitic flatworms including *S*. *mansoni*^12–15,26,28–30^. Our analyses showed that these kinases were expressed in all intra-mammalian stages of *F*. *hepatica*. Furthermore, interesting expression patterns were detected throughout development. The potential polo-like kinase 1 ortholog Fh*plk1* was found to be highly expressed in adults but low in NEJs or immature flukes. This might suggest a role of Fh*plk1* particularly for the mature stage. A similar expression pattern was observed in *S*. *mansoni*, where Sm*plk1* expression was mainly found in germinal cells of adult worms^15^. Accordingly, inhibition or RNAi of Sm*plk1* affected egg production and gonad morphology^13,15^. Opposite to Fh*plk1*, orthologs of the two Abl kinases and two serine/threonine-protein kinases showed a peak of expression in NEJ. In other organisms, these kinases are amongst others involved in cytoskeleton remodeling in response to extracellular stimuli such as growth factors, and in the regulation of cell metabolism^31–34^. Thus, these kinases might play important roles during early growth and development of flukes, which still has to be substantiated in functional studies in the future.

That kinases were found to be expressed in all intra-mammalian stages appears as a prerequisite for any novel target in *F*. *hepatica*, because new compounds should preferably be able to hit all developmental stages in the final host, as does the current gold standard triclabendazole^17^. Whether the significantly different mRNA expression levels between parasite stages found for most kinases will lead to a difference in susceptibility to target inhibition should be part of future studies. A first target gene validation can be achieved by knockdown using RNAi. For such an *in vitro* culture experiment, it should be taken into account that according to our findings, kinase transcript levels significantly change during 4 weeks of culture. Furthermore, for all studied kinases, inhibitor treatment *in vitro* has revealed anthelmintic effects on other parasitic flatworms^12,14,15,26,28–30^. Thus, it is certainly worth testing several of the known kinase inhibitors, such as imatinib and BI 2536, against the different stages of *F*. *hepatica* in near future. To this end, first results obtained by us indicate that imatinib has also the potential of killing *Fasciola in vitro* (Haeberlein, unpublished results).

To conclude, for future expression analyses by qRT-PCR in *F*. *hepatica* we propose using the glutamyl-prolyl-tRNA synthetase Fh*eprs* and tubulin-specific chaperone D Fh*tbcd* as reference genes for studies dealing with the different intra-mammalian fluke stages. Beyond that, we suggest using Fh*tbcd* and the proteasome subunit beta type-7 Fh*psmb7* for studies on *in vitro*-cultured juvenile flukes, such as for RNAi experiments. Especially for inter-stage comparisons, these new reference genes have the potential to replace the traditional housekeeping gene *gapdh* which is used in many *Fasciola* studies to date^9,20,21^, but turned out to be differentially expressed during fluke development in our analysis. We also like to motivate, as a good laboratory practice, to newly validate the suitability of reference genes for studies that use different experimental setups than ours, such as extended drug treatment studies. Using the newly defined reference genes from our study, we quantified expression of kinase orthologs in all relevant intra-mammalian life stages important for drug targeting, which revealed distinct expression patterns throughout development pointing to interesting biological functions. Together with the previously identified broad anthelminthic activity of some kinase inhibitors, this motivates for validation experiments on kinases as potential targets in *F*. *hepatica*.

## Material and Methods

### Ethics statement

Animal experiments were performed in accordance with the German Animal Welfare act in addition to national and international guidelines for animal welfare and were approved by the ethics commission of the Institutional Animal Care and Use Committee (IACUC) of the German Lower Saxony State Office for Consumer Protection and Food Safety (*Niedersaechsisches Landesamt für Verbraucherschutz und Lebensmittelsicherheit*) under reference number 33.8-42502-05-118A336.

### Parasites

Metacercariae from an Italian strain of *F*. *hepatica* were purchased from Ridgeway Research (UK). Excystment was done as previously described with some modifications^9^. Briefly, the outer layer of the metacercariae was physically removed using a scalpel followed by 3–5 min exposure to 10% bleach (v/v). Metacercariae were then incubated in excystment solution (0.6% w/v sodium bicarbonate, 0.45% w/v sodium chloride, 0.4% w/v sodium tauroglycocholate, 0.025 M HCl, 0.4% w/v L-cysteine) for at least 1–2 h at 37 °C and 5% CO_2_ until NEJs started to hatch. NEJs were collected in complete RPMI medium (containing 1% ABAM-solution (10,000 units penicillin, 10 mg streptomycin and 25 mg amphotericin B per ml)) and snap-frozen in liquid nitrogen at 24 h after excystment. Immature and adult worms were harvested from livers of sheep experimentally infected with 250 metacercariae at week 4 and 12 post-infection, respectively. Worms were kept for 1 h in 0.9% NaCl (w/v) to allow clearance of gut contents. All parasite stages were snap-frozen in liquid nitrogen. Samples were stored at −80 °C until further usage.

### *In vitro* culture

In order to grow juvenile *F*. *hepatica in vitro*, NEJs were incubated on day 1 post excystment in complete RPMI1640 medium supplemented with 50% chicken serum at 37 °C and 5% CO_2_^8^. Medium was changed regularly (2–3 times per week). Juveniles were incubated in density of 10 juveniles per ml. At week 4 of culture, juveniles were harvested, snap-frozen in liquid nitrogen and stored at −80 °C until further usage. To study stability of reference gene expression after anthelmintic exposure, adult *F*. *hepatica* were cultured for 2 days in complete RPMI1640 with 5% chicken serum and supplemented with 10 µM and 20 µM triclabendazole (dissolved in DMSO), or supplemented with DMSO as present in the highest drug concentration as a negative control. Medium and compounds were refreshed after 24 h and worms snap-frozen in liquid nitrogen and stored at −80 °C until RNA extraction.

### RNA isolation and cDNA synthesis

Total RNA from all life stages was extracted using the Monarch total RNA Miniprep kit (New England BioLabs) following the manufacturer’s protocol. In brief, NEJs, *in vitro*-grown juveniles, and immature worms were incubated in 300 µl of 1x RNA/DNA protection buffer. Adult worms were chopped in pieces and incubated in 600 µl of the reagent. All samples were subjected to mechanical homogenisation using pestles. Sample sizes ranged between 30–40 NEJs per replicate, 5–10 *in vitro*-grown juvenile worms, and 1 each for immature and adult worms. RNA quality and quantity were checked by electropherogram analysis using the BioAnalyzer 2100 and an Agilent RNA 6000 Pico or Nano Chip according to the manufacturer’s instructions (Agilent Technologies, USA). Synthesis of cDNA was performed using the QuantiTect Reverse Transcription Kit (QIAGEN, Germany) comprising a genomic DNA wipeout step and 11 ng of total RNA per reaction. cDNAs were diluted 1:10 before being used as template in qRT-PCR.

### Quantitative real-time PCR

All primers used for qRT-PCR experiments were designed for a melting temperature of 60 °C and an amplicon size of 140–200 bp (Supplementary Table S2), using the Primer3Plus software tool^47^. When possible, primer pairs were located on different exons of a gene to distinguish amplification of contaminating genomic DNA by size. Prior to qRT-PCR, all primer pairs were tested under standard PCR conditions using the FirePol taq polymerase (Solis BioDyne, Estonia). PCR products were checked for specificity and occurrence of primer dimers on a 2% agarose gel. Only primer pairs yielding in one specific product with no primer dimers were further used for qRT-PCR. Appropriate PCR products were gel extracted (GeneJET gel extraction kit; Thermo Scientific, USA) and used to prepare a standard-curve with 1:10 dilution steps to test primer efficiencies^48^. Only primers with an efficiency of 90–100% were used for subsequent analyses. Primers were commercially synthesised by Integrated DNA Technologies IDT (USA).

The 2x PerfeCTa SYBR Green SuperMix (Quantabio, USA) was used in qRT-PCRs for the detection of synthesised DNA double strands in a final volume of 10 µl and 400 nM of each primer. Analysis was performed on a Rotorgene Q cycler (QIAGEN, Germany) with the following conditions: initial denaturation step at 95 °C for 3 min, 45 cycles at 95 °C for 10 sec, 60 °C for 15 sec, and 72 °C for 20 sec. Melting point analyses were performed for each primer pair to verify primer specificity and to exclude the generation of primer dimers or unspecific side-products. All qRT-PCRs were performed in three to four biological replicates with three technical replicates for each sample. Amplified PCR products were calculated by absolute quantification against a standard curve^49^. The expression of genes of interest was determined by relative quantification against the geometric mean of two selected reference genes. Relative expression levels were calculated by expressing the data as n-fold difference by the formula: relative expression = 2^−delta Ct^ × f, with f = 1000 as an arbitrary factor.

### Evaluation of expression stability of reference genes

Four different software algorithms were used to determine the transcription stability of selected candidate reference genes: NormFinder, geNorm, BestKeeper, and the comparative ΔCT method^50–53^. Two separate sets of analyses were performed. On the one hand, all samples from NEJs, immature, and adult worms were analysed to obtain the most stably transcribed genes for studies dealing with different life stages of the parasite. On the other hand, all samples from NEJs and *in vitro*-grown juvenile worms were used to reveal the best reference genes for gene expression studies in *in vitro*-culture experiments.

The algorithm NormFinder determines intra- and inter-group variations across the different samples to calculate a stability value (M). Low variations give a low stability value, which indicates stable transcription of a gene. As input data, the calculated concentrations of qPCR amplification were used^50^. The geNorm algorithm calculates pairwise variations of each reference gene when compared with the other genes based on relative Ct values. The stability value (M) is based on the average of these pairwise variations. Again, a stable transcription is reflected by a low M value. BestKeeper analysis was performed on raw Ct values. This algorithm assumes that stable reference genes should display similar transcription patterns, i.e. are highly correlated to each other. This is reflected by a high coefficient of correlation (r), whereby the most stably transcribed genes exhibit values closest to 1. The comparative ΔCT method compares the difference in Ct values of reference genes in pairs. Ranking is based on the variability of averaged standard deviations^53^.

### *In silico* analyses

Eight candidate reference genes and five kinase genes of *F*. *hepatica* were identified by BLASTp search of the known human orthologs against the genome of *F*. *hepatica* (Centre for Genomic Research, University of Liverpool, BioProject ID PRJEB25283) using the public domain tool WormBase ParaSite, version WBPS13 (https://parasite.wormbase.org)^54^. Gene names, biological function, and accession numbers of *H*. *sapiens* and *F*. *hepatica* are listed in Tables 1 and 2. The BLASTp cutoff for the identification of potential orthologs was 5E-72. The identity of the potential *F*. *hepatica* orthologs was confirmed by analysis of conserved protein domains using SMART (http://smart.embl-heidelberg.de/)^55^, and by multiple alignment of amino acid sequences against the sequences of several model species or related species (*H*. *sapiens*, *M*. *musculus*, *C*. *elegans*, *D*. *melanogaster*, *S*. *mansoni*) using CLUSTALW. The accession numbers for all species used for multiple alignment are listed in Supplementary Table S1.

### Statistical analysis

Statistically significant differences between samples were determined by t-test. Error bars represent the standard error of the mean (SEM). *p*-values < 0.05 were considered significant.

## Supplementary information

Supplementary

## Data Availability

All data generated or analysed during this study are included in this published article and its Supplementary Information Files.
