# Supplementary material for: Reference gene analysis and its use for kinase expression profiling in Fasciola hepatica
Source: Sci Rep. 2019 Nov 1;9:15867. doi: 10.1038/s41598-019-52416-x (PMC6825121; doi:10.1038/s41598-019-52416-x)
Supplement: Supplementary file 1 — Supplementary [file 41598_2019_52416_MOESM1_ESM.pdf]

Supplementary Information

Reference gene analysis and its use for kinase expression profiling in *Fasciola hepatica*

Hicham Houhou, Oliver Puckelwaldt, Christina Strube, Simone Haeberlein

Supplementary Table S1: Accession numbers of reference genes and kinase genes used for the multiple alignments in Supplementary Figures S8 and S9.

Gene	*F. hepatica*	*H. sapiens*	*M. musculus*	*D. melanogaster*	*C. elegans*	*S. mansoni*
tbcd	maker-scaffold10x_815_pilon-snap-gene-1.87	NP_005984.3	NP_084154.1	NP_608648.1	NP_492270.1	Smp_153590
eprs	maker-scaffold10x_14_pilon-snap-gene-0.109	NP_004437.2	NP_084011.1	NP_524471.2	NP_492711.1	Smp_138930
letm1	maker-scaffold10x_721_pilon-snap-gene-0.10	NP_036450.1	NP_062668.1	NP_611922.1	NP_506382.1	Smp_310830.1
actb	augustus_masked-scaffold10x_269_pilon-processed-gene-0.18	NP_001092.1	NP_780706.1	NP_001014725.1	NP_505818.1	Smp_183710
snrpa1	maker-scaffold10x_234_pilon-snap-gene-0.20	NP_003081.2	NP_067311.4	NP_610315.1	NP_494763.1	Smp_141330
ppp1cb	maker-scaffold10x_238_pilon-snap-gene-0.95	NP_002700.1	NP_001357876.1	NP_001262919.1	NP_001022616.1	Smp_028990
psmb7	maker-scaffold10x_1452_pilon-augustus-gene-0.11	NP_002790.1	NP_035317.1	NP_524076.2	NP_493271.1	Smp_073410
gapdh	maker-scaffold10x_2706_pilon-snap-gene-0.15	NP_001276674.1	NP_032110.1	NP_001259584.1	NP_508534.3	Smp_056970.2
abl1	maker-scaffold10x_1995_pilon-snap-gene-0.46	NP_005148.2	NP_001269975.1	NP_001261964.1	NP_509777.2	Smp_246700.1
abl2	maker-scaffold10x_873_pilon-snap-gene-0.69	NP_001129473.1	NP_001129576.1	NP_476849.1	NP_493502.1	Smp_128790.1
akt1	maker-scaffold10x_205_pilon-augustus-gene-0.40	NP_001014431.1	NP_001318036.1	NP_001287353.1	NP_001023646.1	Smp_243630
pkc	maker-scaffold10x_608_pilon-snap-gene-0.5	NP_002731.4	NP_032883.2	NP_001036542.1	NP_495011.1	Smp_096310
plk1	maker-scaffold10x_784_pilon-snap-gene-0.36	NP_005021.2	NP_035251.3	NP_001014592.1	NP_491036.1	Smp_009600

Supplementary Table S2: Primer sequences and product sizes in qRT-PCR

Gene	Annotation	Forward primer (5'-3')	Reverse primer (5'-3')	PCR product size (bp)
tbcd	Tubulin-specific chaperone D	CAGCAGCCGCATTTCAGGA	AGCCAAATGGTCAATCATCGC	176
eprs	Glutamyl-prolyl-tRNA synthetase	TACACCACAACCATCGAGGC	GTGGTCAATCCCCACGAGTT	167
letm1	Leucine zipper and EF-hand containing transmembrane protein 1	AGATGGATAAGTTAGCCGAGG	ACCTTTTGGAGACGCCGTAG	184
actb	Actin, cytoplasmic 1	CATGTTTGAGACCTTCAACGCT	AGATCACGCCCAGCAAGGT	189
snrpa1	U2 small nuclear ribonucleoprotei n A'	GAGAACATGGGTAGTACATTGG	CCAGATCCTCTGCTATGCG	148
ppp1cb	Protein phosphatase 1 catalytic subunit beta	GCAGTTGGTTACTTTGTTTTCGG	CAGGCCATACCGCATTGAG	161
psmb7	Proteasome subunit beta type 7	TCACCAAGGATTCCACGGAG	GATCTGGCAGATCCCGAATC	171
gapdh	Glyceraldehyde-3-phosphate dehydrogenase	GCCAATGTTCGTGTTCGGAG	TCAACGACCTTCTGAGTGGC	183
abl1	ABL proto-oncogene 1,non-receptor tyrosine kinase	CTGCCTGTACATACTGTGCC	GTGCGGTGAGTTCATGGTTC	154
abl2	ABL proto-oncogene 2,non-receptor tyrosine kinase	CGTTAGCGAATCCCAATCTAG	GGTTCTCTGTGTAGTGACCG	179
akt1	Rac-alpha serine/threonine-protein kinase	TCTCGGTGGTGGTCCTAGT	TATGTACTTCGTGTCCGTAGG	142
pkc	Protein kinase C	TAAAGAGGGCATCACAGCGG	GCCAACATTTTCCCACGGAG	172
plk1	Polo-like kinase 1	ATGGCTAGTAAGGACGCTGC	TGCTTTCGGAACCACTTTTCC	177

Supplementary Table S3: **Expression stability of candidate reference genes based on three different life stages of *F. hepatica*: newly excysted juveniles (NEJs), immature worms, and adults.**

Ranking was based on four different algorithms: NormFinder, BestKeeper, geNorm, and comparative ΔCT method. Candidate reference genes selected for further analyses are written in bold.

	**NormFinder**		**BestKeeper**		**geNorm**		**ΔCT method**	
**Rank**	**Ref. gene**	**Stability value M**	**Ref. gene**	**Coeff. of correlation r**	**Ref. gene**	**Stability value M**	**Ref. gene**	
1	**Fh*eprs***	0.118	Fh*actb*	0.972	**Fh*tbcd*, *Fheprs***	0.298	**Fh*eprs***	0.562
2	Fh*psmb7*	0.194	**Fh*tbcd***	0.971			**Fh*tbcd***	0.600
3	**Fh*tbcd***	0.209	**Fh*eprs***	0.970	Fh*psmb7*	0.360	Fh*psmb7*	0.618
4	Fh*snrpa1*	0.306	Fh*psmb7*	0.936	Fh*snrpa1*	0.415	Fh*snrpa1*	0.661
5	Fh*actb*	0.361	Fh*gapdh*	0.922	Fh*letm1*	0.513	Fh*actb*	0.800
6	Fh*letm1*	0.366	Fh*snrpa1*	0.810	Fh*ppp1cb*	0.597	Fh*letm1*	0.819
7	Fh*ppp1cb*	0.540	Fh*letm1*	0.610	Fh*actb*	0.672	Fh*ppp1cb*	0.966
8	Fh*gapdh*	0.592	Fh*ppp1cb*	0.305	Fh*gapdh*	0.751	Fh*gapdh*	0.989

Supplementary Table S4: **Expression stability of candidate reference genes during *in vitro* growth of *F. hepatica* from newly excysted juveniles (NEJs) to 4 week old juvenile worms.**

Ranking was based on analysis using NormFinder, BestKeeper, geNorm and comparative ΔCT method. Candidate reference genes selected for further analyses are written in bold.

	**NormFinder**		**BestKeeper**		**geNorm**		**ΔCT method**	
**Rank**	**Ref. gene**	**Stability value M**	**Ref. gene**	**Coeff. of correlation r**	**Ref. gene**	**Stability value M**	**Ref. gene**	
1	**Fh*psmb7***	0.177	**Fh*psmb7***	0.971	**Fh*tbcd*,** Fh*eprs*	0.170	**Fh*tbcd***	0.564
2	**Fh*tbcd***	0.196	**Fh*tbcd***	0.945			**Fh*psmb7***	0.577
3	Fh*eprs*	0.242	Fh*snrpa1*	0.935	**Fh*psmb7***	0.269	Fh*eprs*	0.600
4	Fh*snrpa1*	0.263	Fh*eprs*	0.883	Fh*actb*	0.317	Fh*snrpa1*	0.661
5	Fh*actb*	0.340	Fh*ppp1cb*	0.817	Fh*snrpa1*	0.424	Fh*actb*	0.686
6	Fh*letm1*	0.469	Fh*actb*	0.730	Fh*gapdh*	0.532	Fh*letm1*	0.902
7	Fh*ppp1cb*	0.596	Fh*letm1*	0.670	Fh*letm1*	0.651	Fh*gapdh*	0.988
8	Fh*gapdh*	0.620	Fh*gapdh*	0.201	Fh*ppp1cb*	0.756	Fh*ppp1cb*	1.072

Tubulin-specific chaperone D (FhTbcd):

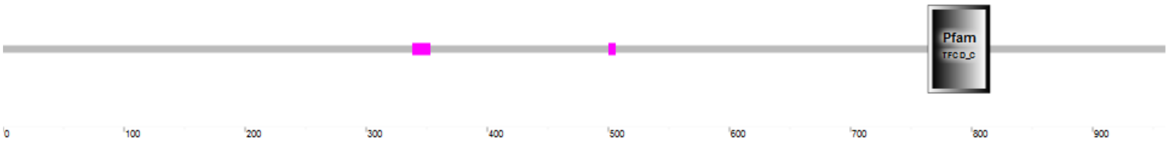

Glutamyl-prolyl-tRNA synthetase (FhEprs):

ProRS-C_1, Prolyl-tRNA synthetase, C-terminal

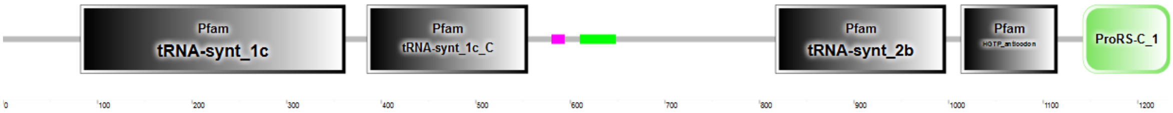

Actin, cytoplasmic 1 (FhActb):

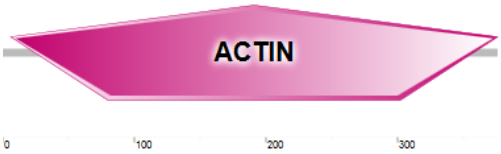

U2 small nuclear ribonucleoprotein A (FhSnrpa1):

LRR, Leucine-rich repeat

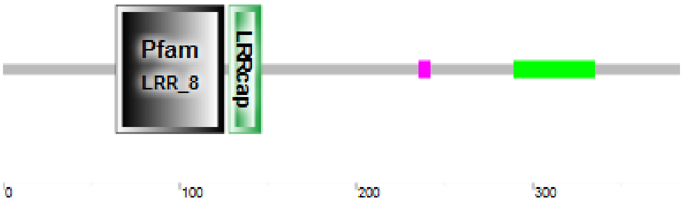

Leucine zipper and EF-hand containing transmembrane protein 1 (FhLetm1):

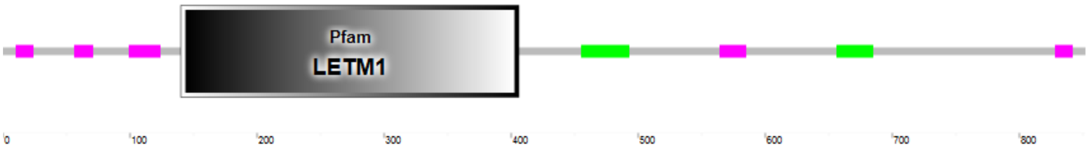

Glyceraldehyde-3-phosphate dehydrogenase (FhGapdh):

Gp_dh_N, Glyceraldehyde 3-phosphate dehydrogenase, NAD binding domain

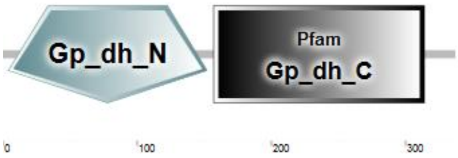

Protein phosphatase 1 catalytic subunit beta (FhPpp1cb):

PP2Ac, Protein phosphatase 2A homologues, catalytic domain

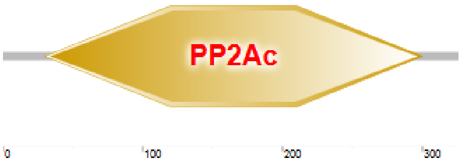

Proteasome subunit beta type 7 (FhPsmb7):

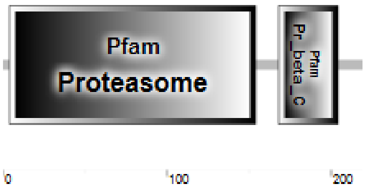

Supplementary Figure S1: **Results from SMART analysis of amino acid sequences from eight reference gene candidates of *F. hepatica*.**

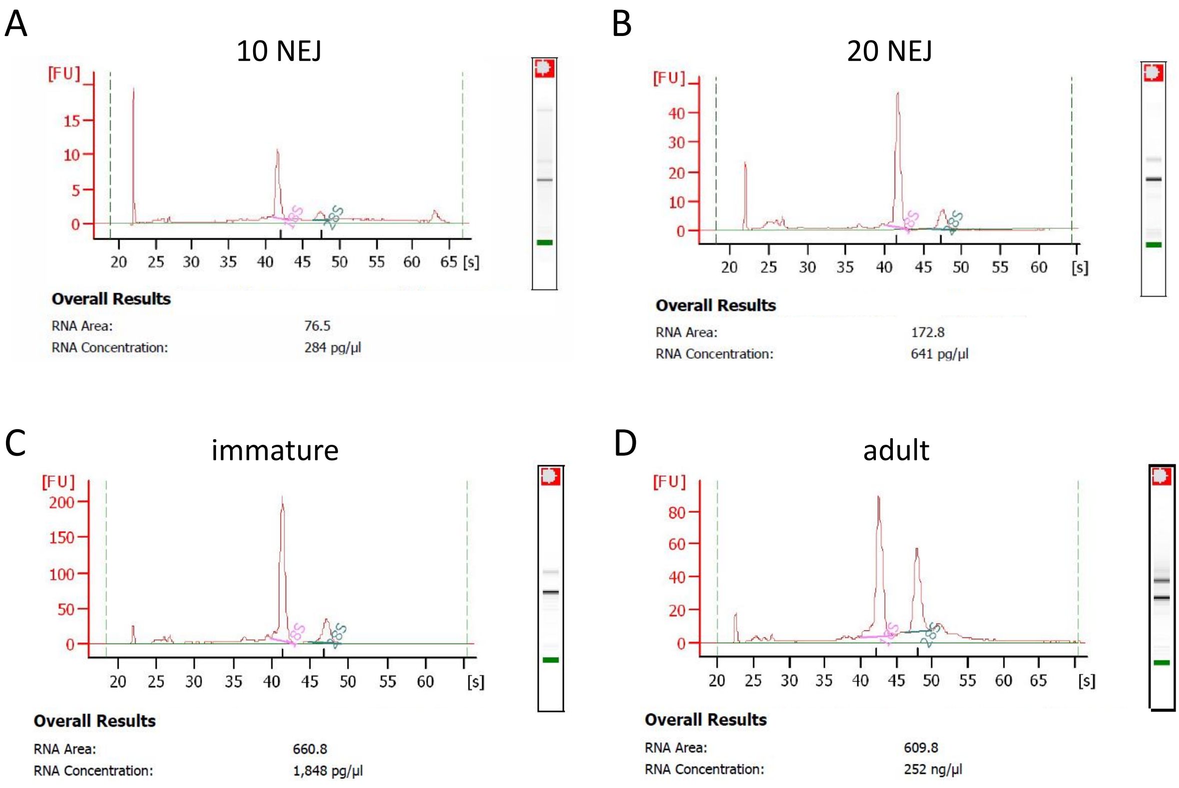

Supplementary Figure S2: **RNA quantity and quality of liver fluke samples from BioAnalyzer analysis.** Electropherograms of RNA extracted from 10 or 20 NEJs (A, B), 4-week old immature (C) or 12-week old adult flukes (D). Agilent RNA 6000 Nano Chips (adult) or Pico Chips (others) were used.

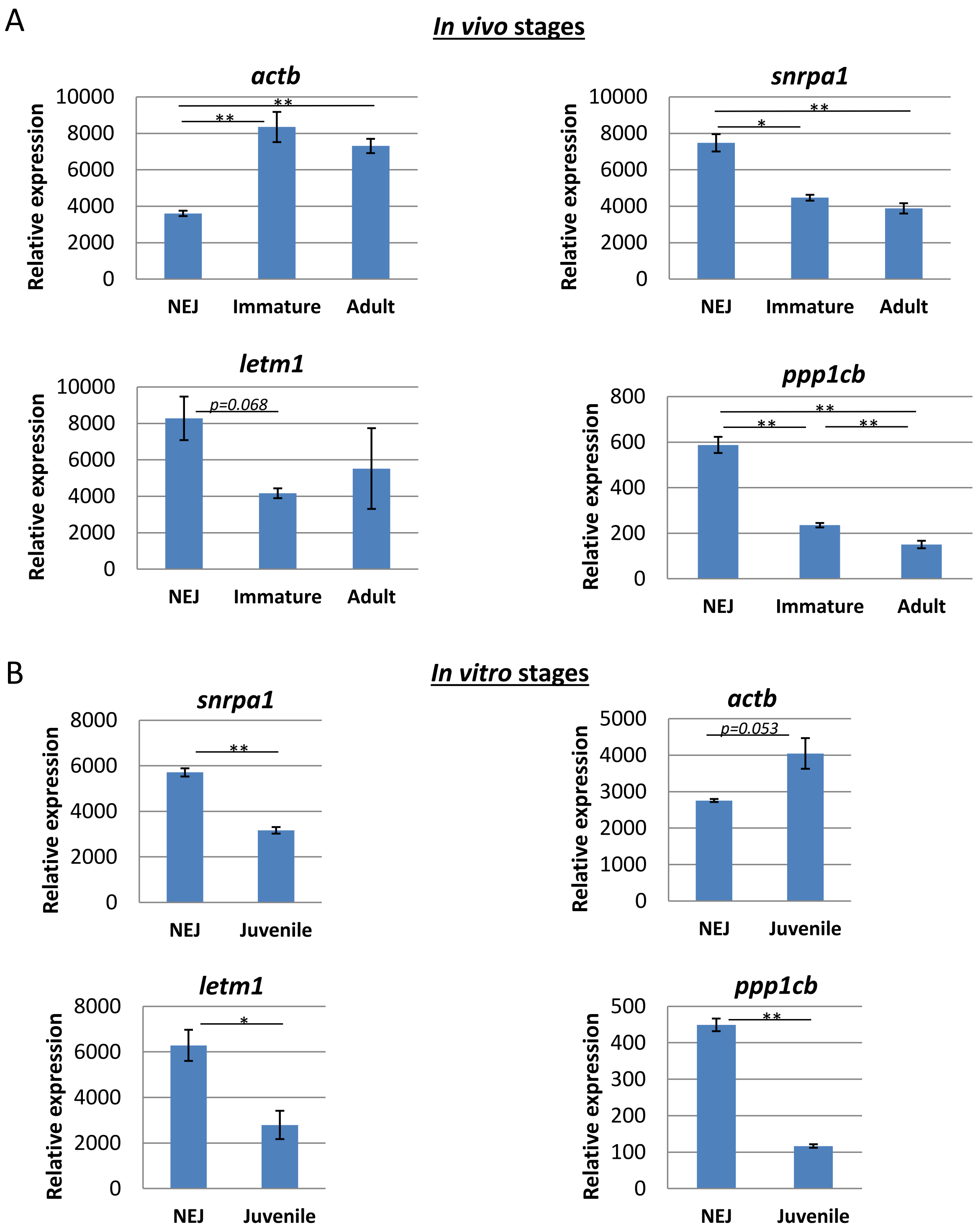

Supplementary Figure S3: **Relative expression levels of reference gene candidates in different intra-mammalian stages of *F. hepatica*.** Data were normalised against the geometric mean of the two most stably expressed genes previously identified (*tbcd* and *eprs* for A, *tbcd* and *psmb7* for B). Average values of 3-4 biological replicates with SEM are shown. Significant differences are indicated with * p<0.05, ** p<0.01 (t-test).

ABL proto-oncogene 1, non-receptor tyrosine kinase *(*FhAbl1*)***:**

SH2, Src homology 2 domain; SH3, Src homology 3 domain; TyrKc, Tyrosine kinase, catalytic domain

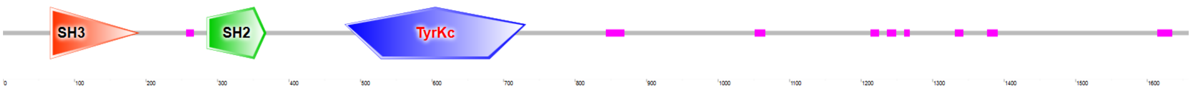

ABL proto-oncogene 2, non-receptor tyrosine kinase (FhAbl2)**:**

SH2, Src homology 2 domain; SH3, Src homology 3 domain; TyrKc, Tyrosine kinase, catalytic domain

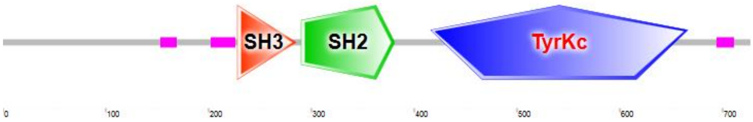

Rac-alpha serine/threonine-protein kinase (FhAkt1)**:**

S_Tkc, Serine/Threonine protein kinases, catalytic domain; S_TK_X, Extension to Ser/Thr-type protein kinases

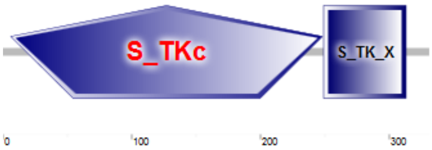

Protein kinase C (FhPkc)**:**

PB1, Phox and Bem1p domain; C1, Protein kinase C conserved region 1 (C1) domain; S_Tkc, Serine/Threonine protein kinases, catalytic domain; S_TK_X, Extension to Ser/Thr-type protein kinases

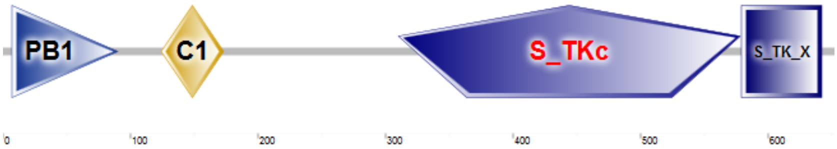

Polo-like kinase 1 (FhPlk1)**:**

S_Tkc, Serine/Threonine protein kinases, catalytic domain;

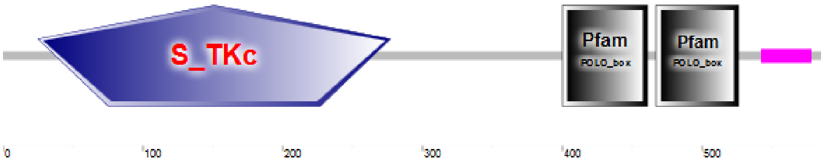

Supplementary Figure S4: **Results from SMART analysis of amino acid sequences from five kinases of *F. hepatica*.**

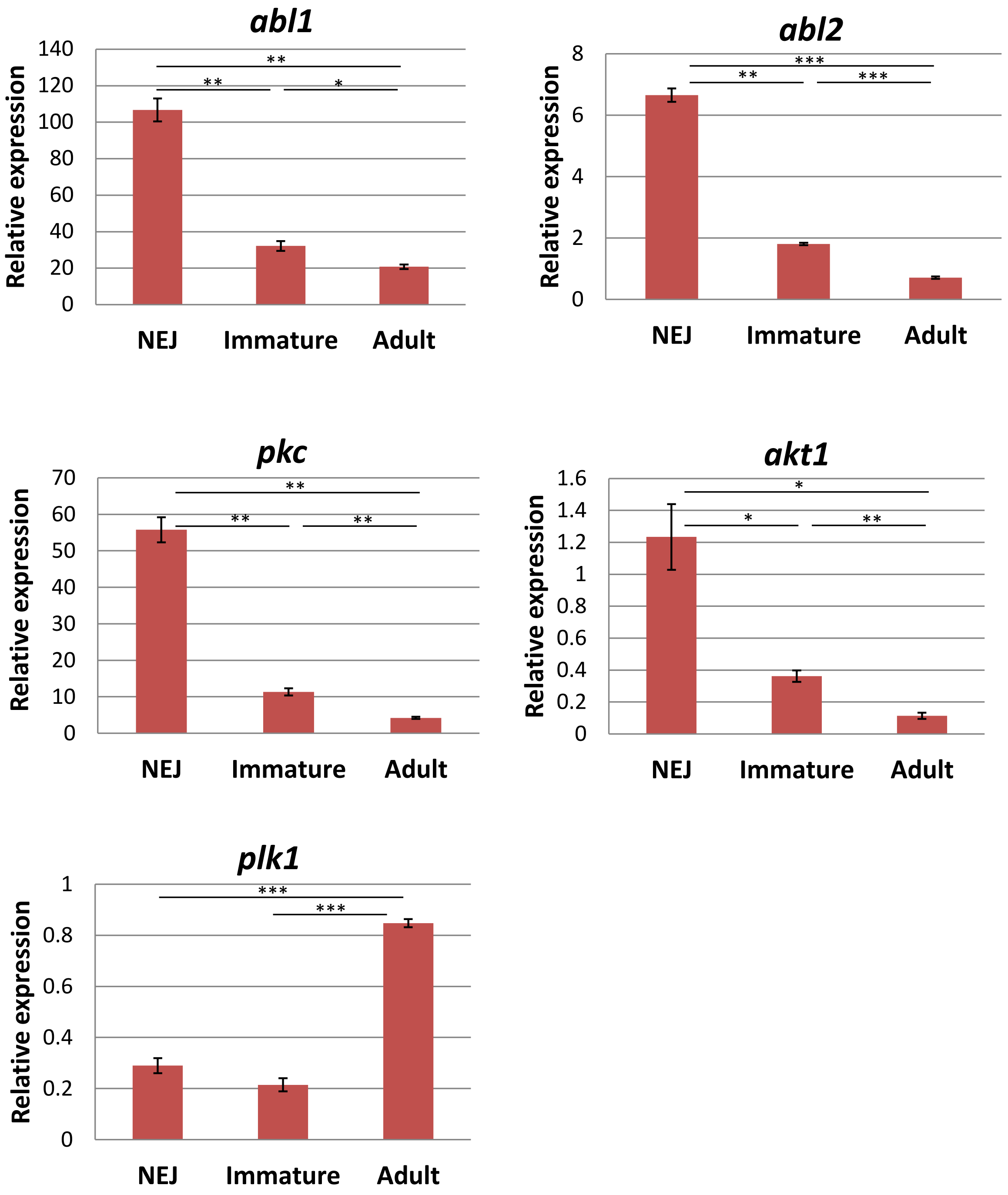

Supplementary Figure S5: **Relative expression levels of kinases in three different intra-mammalian life stages of *F. hepatica* normalised against *gapdh*.** Expression data from NEJs, 4 week-old immature, and 12 week-old adult worms were normalised against the geometric mean of the least stably expressed reference gene candidate, *gapdh*. Average values of 3-4 biological replicates with SEM are shown. Significant differences are indicated with * $p<0.05$, ** $p<0.01$, *** $p<0.001$ (t-test).

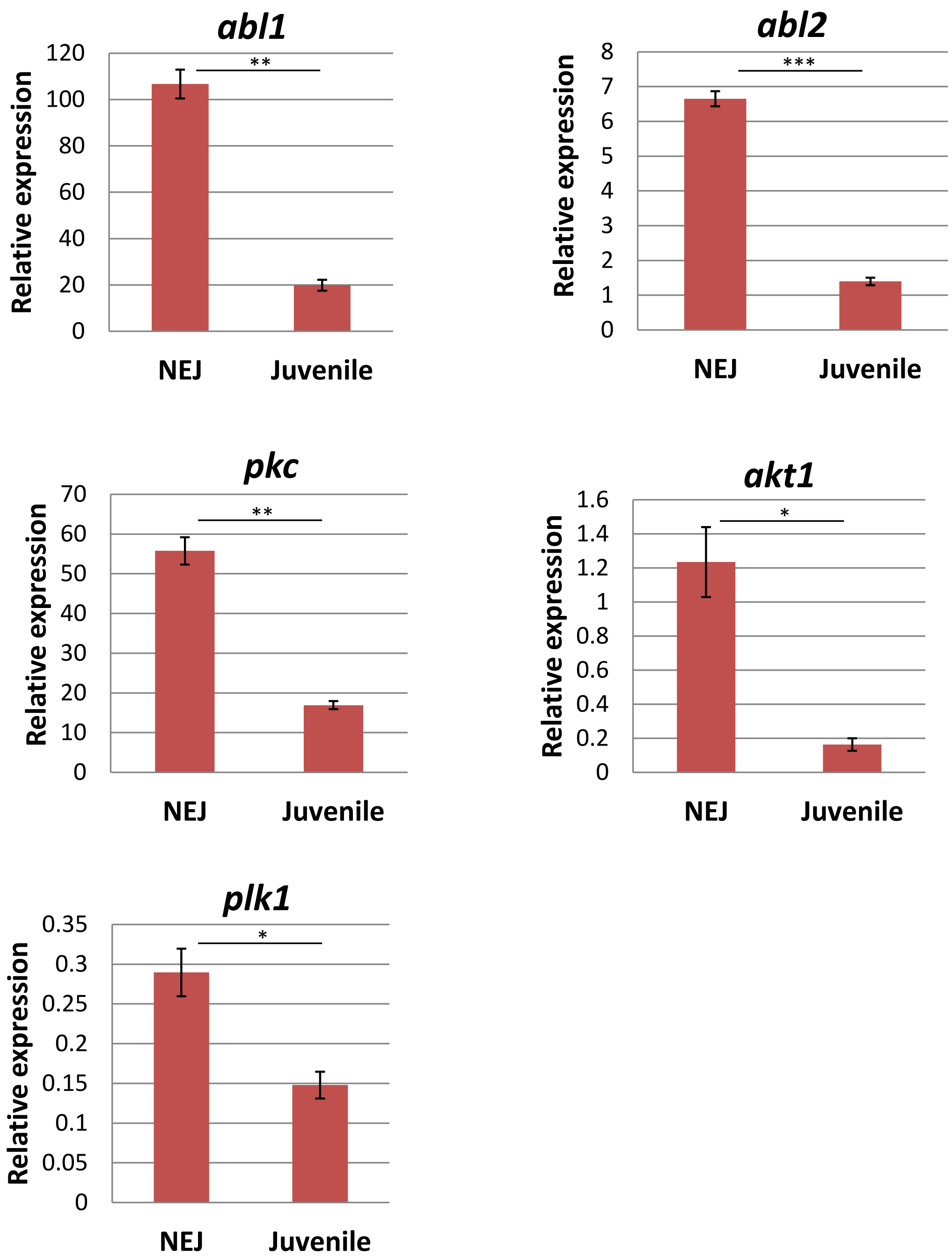

Supplementary Figure S6: **Relative expression levels of kinases during *in vitro* culture of juvenile *F. hepatica* normalised against *gapdh*.** Expression data from NEJs and juvenile worms grown for 4 weeks in serum-rich medium were normalised against the geometric mean of the least stably expressed reference gene candidate, *gapdh*. Average values of 3-4 biological replicates with SEM are shown. Significant differences are indicated with * $p<0.05$, ** $p<0.01$, *** $p<0.001$ (t-test).

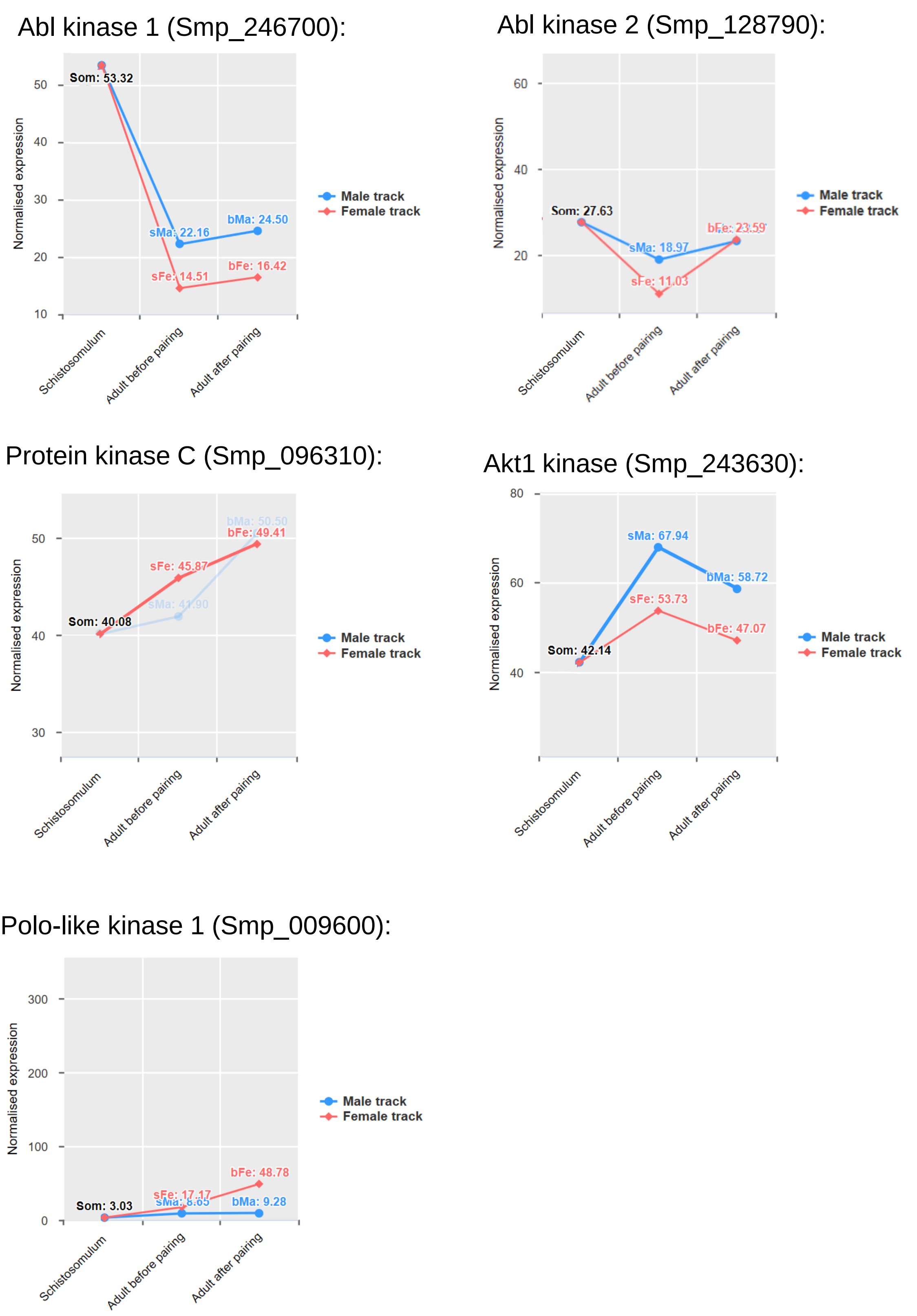

Supplementary Figure S7: Relative expression of kinase orthologs in *Schistosoma mansoni* schistosomula and adult stages. Data were extracted from the meta-analysis by Lu et al. 2017 available at https://meta.schisto.xyz/.

Supplementary Figure S8: Multiple alignment of amino acid sequences of orthologs for reference gene candidates. Accession numbers of gene orthologs for *C. elegans* (Ce), *F. hepatica* (Fh), *S. mansoni* (Sm), *D. melanogaster* (Dm), *H. sapiens* (Hs) and *M. musculus* (Mm) are listed in Supplementary Table S1. Conserved protein domains are highlighted. * indicates identical amino acids.

Tubulin-specific chaperone D (Tbcd):

```
Ce      MKKTKYPTLVRISRFLFTDLVSMKIRFQRAWLRNVFADKTTRLTRKCSSSMLHLREFLQF 60
Fh      ------------------------------------------------------------ 0
Sm      ------------------------------------------------------------ 0
Dm      ------------------------------------------------------------ 0
Hs      ------------------------------------------------------------ 0
Mm      ------------------------------------------------------------ 0


Ce      LGFLLKYLSMDEDAPVDESNEGIIGCLPSAVNIHHIEEIKELVDQLPIIFKLENECDETA 120
Fh      ---------MD-SNRDLLPEEEQFLIFN---NFKHYDEVIKLISTITDATK-----LQTT 42
Sm      ------------------------------------------------------------ 0
Dm      ----------M-SNSVEECKDEDL-PANTLEHFTELQQVLEMIDNIKSIAA-----NTF- 42
Hs      --MALSDEPAA-GGPEEEAEDETLAFGAALEAFGESAETRALLGRLREVHG-----GGAE 52
Mm      --MVLSNEPAA-SAAEEEVEDDALVRASALEAFGESAETRALLRSLPAVHR-----ERAS 52


Ce      AEVNYLRYSRLLHLYQEQPRLLDKWIPEIVANLVDLVTLIGIDVSKPRAMTPLSRESLKY 180
Fh      EEHLEEEFIKIFGFYQEQPHLLDPYLPKMISDTLEVVK-------TTKTGSRAFHFAFRV 95
Sm      ------------------------------------------------------------ 0
Dm      -EREFEQYAQVLSRYQEQPHLLDPHLEELLGKLLHKIR-------KPDLDTGELHAAFKY 94
Hs      REVALERFRVIMDKYQEQPHLLDPHLEWMMNLLLDIVQ-------DQTSPASLVHLAFKF 105
Mm      REVAEERFRVIMDKYQEQPHLLDPHLEWMMNSLLDLVQ-------DETSLPDLVHLAFKF 105


Ce      LSDLCIVRGSKTIVRLLPHQVHLLDPLLQTLEYYETSQLSDHNQRNVLLMWLWIVVKNPF 240
Fh      LYLMVKTRGYKSIIRLMPHTVDDIEPTL------------TWETRYVLLLWLSILIMVPF 143
Sm      ---MTKTRGYKAIIRLMPHTVDDIEPTLSLLMEQDINDSKNWETRYVLILWLSILVMVPF 57
Dm      LYIICKVRTYKVLVKFMPHELSDLEFVLDLLGQQNPKEFEQWETRYILLLWMSILVLNPF 154
Hs      LYIITKVRGYKTFLRLFPHEVADVEPVLDLVTIQNPKDHEAWETRYMLLLWLSVTCLIPF 165
Mm      LYIITKVRGYKVFLRLFPHEVANVQPVLDMFTGQNPKDHETWETRYMLLLWLSVTCLIPF 165
           :  .*  * :::::** :  ::  *              : * :*::*: :    **

Ce      DLRRFDPTG-----------------DPDNVITRIMNVALHYMKWDWNSSQASAALVIAH 283
Fh      GLNCLDSE------------------DRAPIVNRILDQSKRYLSLDGRTQ-EAASFLLAR 184
Sm      NLESLDSF------------------GKKPIIERVIDLAKLYLLQDERTQ-EAAAFLLAH 98
Dm      HMSRLDAYDTSTSAPTTNCSPVNHVQSKNTKMDRIFELIQLYVSSNDTCS-SMAAFLAAK 213
Hs      DFSRLDGNLLTQP-----------GQARMSIMDRILQIAESYLIVSDKAR-DAAAVLVSR 213
Mm      DFSRLDGNLSTQT-----------GETRVPTMDRILQIAESYLVVSDKAR-DAAAVLVSK 213
         :  :*                         : *:::    *:  .       *:.: ::

Ce      CLSRTDGIPKV-LSFLSRLLDSIKT----HHENKKLLLADLILLLAILKHVDRRVLTGHI 338
Fh      LVTRPDVVQAHLTPVLDWCLEQIKNADCTTGKGQKLLCGVLRSLANICKVGRRQELLAHA 244
Sm      TVTRPDALHAQLPSIISSAIKNLSFADVINVQDQKQVCGTLRSIANICKLGSRTELLPYA 158
Dm      YFIRSDIKDLYLERFLDWIMEQHQADTLN------VKFGQLAAVAAILKHGKREDLLPYA 267
Hs      FITRPDVKQSKMAEFLDWSLCNLARSSFQTMQGVITMDGTLQALAQIFKHGKREDCLPYA 273
Mm      FITRPDVKQRKMASFLDWSLCTLAHSSFQTIEGVITMDGMLQALAQIFKHGKREDCLPYA 273
         . * *        .:.  :                  . *  :  * *   *     :

Ce      GTIHEQLSFLYPIDEKKGGLICKCLVKVVQRIGLIALKPRTCSWSYNRGKRLLEGMLDDN 398
Fh      PRLLES-ILHMSIAAAKGNWIYRFETKLLQRIGLLFCPPRSFSWQYQRGLRSLADNLAPR 303
Sm      SDLLSA-VLQLPGDSSKGILLCRLETKVLQRIGLLFCPPINTTWQYQRGSRSLQENLESL 217
Dm      DKLLQW-ITSCQYKDDNDFLKYKNYVKIIQRIGLVHLKPRIASWRYKRGTRSLATNLNQT 326
Hs      ATVLRC-LDGCRLPESNQTLLRKLGVKLVQRLGLTFLKPKVAAWRYQRGCRSLAANLQLL 332
Mm      NTVLQC-LDGCRLPESSHTSLRKLGVKLVQRLGLTFLKPKVATWRYQRGCRSLAANLKLC 332
          :             .     :  .*::**:**    *   :* *:** * *   *
```

```
Ce      EEYSDEPSFSNKVNS----NQS----CNNEIDKENQWNDGDELENSEIVEFALMHVLEAL 450
Fh      LRELQNTTDGNTNQA-SNFSTSLERTVTHGLTESTDDDDELDLEHADEVAEVIDRLINSL 362
Sm      LMNHDKNSSVSAKVTDHSSYTSFQNNISSDISGDCSLTMKHEFPNTDEVAEVIDKLISAL 277
Dm      TAAGGEPV-----------------V----LEQSL--EEGEEIVVPDAIEEVIEELLQAL 363
Hs      TQGQSEQK-----------------P----L--ILTEDDDEDDDVPEGVERVIEQLLVGL 369
Mm      APGKSDQK-----------------L----LSDSLTSDGDEDYDVPEGVETVIEQLLVGL 371
             .                        :          :    : :  .: .:: .*

Ce      SHSDTAVRWSAAKGVGRITVRLPNFDLATQVVGSIISSHFGEVAEYSSWHSHGACLALAE 510
Fh      RNQYTVVRWSAAKGLGRMCGRLSR-SMVNDVLSAILL-LCTRLEPFTAW--HGACLALAE 418
Sm      RSQFTGVRWSAAKGIGRICSRLSS-SMVNDVLSAVLS-LCTKLEPYTAW--HGACLALAE 333
Dm      RSGGNDIRWSAAKGLGRVTNRLPK-ELADEVIGSVID-ILNPLEPHEAW--HGACLALAE 419
Hs      KDKDTVVRWSAAKGIGRMAGRLPR-ALADDVVGSVLD-CFSFQETDKAW--HGGCLALAE 425
Mm      KDKDTVVRWSAAKGIGRMAGRLPR-ELADDVVGSVLD-CFSFQETDKAW--HGGCLALAE 427
            . :*******:**:  **    :. :*:.:::           :*  **.******

Ce      LAHRGVLLPSLLEDIVPALELSLVFEDVMGRHQNGNQVRDAACYAVWALSRTYEPSMMAP 570
Fh      LGRRSLLLPSKLPEVMPVILRALFYDERSGDHSYGSNVRDAACYVCWAFARAYQAADLAP 478
Sm      LGRRNLLLPSKLPEVIPVVLRALFYDERSGDHNYGSNVRDAGCYVCWAFARAYHPKDFLD 393
Dm      LAKRGLLLPHRLEELVPLLMQALFYDEMKGYMSVGQHIRDSACYMCWAFARAYNPDDVKP 479
Hs      LGRRGLLLPSRLVDVVAVILKALTYDEKRGACSVGTNVRDAACYVCWAFARAYEPQELKP 485
Mm      LGRRGLLLPSRLSEVVTVILKALTYDEKRGACSVGANVRDAACYVCWAFARAYEPQELTP 487
        *.:*.:***  * :::  :  :* :::  *  . * ::**:.**  **::*:*.   .

Ce      YLQRLASALLCGALFDRQVNLRRAASAALQEMVGRQKNVSHGIPLIQSVDYFAVTNRQKC 630
Fh      YVTQVAQSLVLVSLFDREVNVRRAAAAAFQENVGRQGQFPHGIEILTACDYFAVRNLKNC 538
Sm      YIVPIASSLVLVSLFDREVSVRRAASAAFQENVGR--QFPHGIEILTTCDYFTVGNRAHC 451
Dm      FVHKISSGLLTVAVFDREVNCRRAASAAFQESVGRLGNFPFGIEISTTTDFYSVGIRQNS 539
Hs      FVTAISSALVIAAVFDRDINCRRAASAAFQENVGRQGTFPHGIDILTTADYFAVGNRSNC 545
Mm      FVTAISSALVIAAVFDRNVNCRRAASAAFQENVGRQGTFPHGIDILTTADYFAVGNISNC 547
        ::  ::..*:  ::***::. ****:**:** ***   . .** :  : *:::*    :.

Ce      YEHLCVPVAEYSTYSAIILRHLITKKVVHWDEKIREQAAISLEKISEIRLENVSDDYYME 690
Fh      YLELSAFVAQFPEYTQAMIDHLATKLLGHWDVVIRFLSARALNVLCRFSPE--------- 589
Sm      YLQLSIFVAKFKEYAKPMIDHLVNVRLGHWDDSIRYLAACALGKLYIADPDYMMEIVLPQ 511
Dm      YLNISDYIAQFEVYREPLINHLVQHKVSHWDSAIRELTAKALHKLSLWEPEYMAAVVLPQ 599
Hs      FLVISVFIAGFPEYTQPMIDHLVTMKISHWDGVIRELAARALHNLAQQAPEFSATQVFPR 605
Mm      FLIISVFIAGFQEYTKPMIDHLVSMKINHWDGAIRELSAKALHNLTPQVPEYIAMHVFPA 607
        :  :.  :* :  *   :: **    : ***  **  :* :*  :     :

Ce      ILDDFLKASCETRISPFLRHGYLLASGHLIKGLTSRGMDF------SSKQTEIAWIPHIL 744
Fh      -------------------------------------------DAIGPELLVQIKEIV 604
Sm      IINGS------INSTLHNQQGCIYGTGELVCSS----------SSCVINEENLLKIKEIV 555
Dm      LLAKT------DTIDINCRHGCVLAMGEITLTLRKLEEKSD-PQVVYLSNQRVAELNELI 652
Hs      LLSMT------LSPDLHMRHGSILACAEVAYALYKLAAQENRPVTDHLDEQAVQGLKQIH 659
Mm      LLLMT------QSPDLHTRHGAILACAEVTYALYKLATQSNRLVTDYLDEKAVQSLKQIH 661
                                                            :  : .:

Ce      WPFCD---MTTQPGALIRRTLCKFIQLVSASKKVLLLEKDKSEWLDVLLQLIT------- 794
Fh      PTLSERNQFRGLSGELLRKASCHLIEKSSRTRLPLHGDPVIETWRLLLDDCVA------- 657
Sm      PALKSANKFRGLSGELIRKATAHFIQKCAMAKLPFHDDPIIEVWREFLDDCVG------- 608
Dm      ITFLDKNFYRGMSGDLMKSCTSSYIKNCSLAKLQATPE-CLVSWQKVIDSCLI------- 704
Hs      QQLYDRQLYRGLGGQLMRQAVCVLIEKLSLSKMPFRGDTVIDGWQWLINDTLRHLHLISS 719
Mm      QQLCDRHLYRGLGGELMRQAVCILIEKLSLSRMPFKGDATVEGWQWLINDTLRSLHLVSS 721
          : .        * *::   .  *:  : ::     :     *  .: . :
```

```
Ce      DPREIIRSLAKTAVGEFVMTYLMNDEELIQKV-K----TRVIAAMTKCSDESERIGMGMI 849
Fh      HKEVEIQKTAVSAYTQLLAAYLYDKDKHLQIAYRDKMFGHLTQQLE-ANSETKQSGFLQV 716
Sm      HKNPEVQKATVNAYPHFLSAYLYDRNGELQLGYKDLLYRNFLLQLN-TNSESKLSGYLQI 667
Dm      TKSNAIRDGAVEAFGELCTTYYCSDSRHGE---NEAIINTYLTGADNDLEEHIRMGYIAA 761
Hs      HSRQQMKDAAVSALAALCSEYYMKEPGEADPAIQEELITQYLAEL-RNPEEMTRCGFSLA 778
Mm      HSRQQIKEVAVSALTALCSEYYVKEPGEAGSSIAKELIPQYLAEL-QSPEEMARCGFSSA 780
             ::. :  *   :   *  .                         .*    *

Ce      CESLNSEAVDY---EMFESLCNTILTP-------------TSSDAKWALARQQTVFALNR 893
Fh      LADAPADLFTGHVQKTLELVTAACRISTK---------------TRSWGDARASALKSVLG 762
Sm      IGAAPNSLYCGHVADLLDTVTSACRSTSK---------------TKFWVDSRGSALKALVE 713
Dm      LGVLPSFMIRCHLQAILDSLVKHSLTPLQAVLVGEMGDRENIQAYRWSEARTQSVLALTK 821
Hs      LGALPGFLLKGRLQQVLTGLRAVTHTSPE---------------DVSFAESRRDGLKAIAR 824
Mm      LGALPGFLLRGHLQQVLSGLRRVTCISPN---------------DVSFAEARRDGLKAISR 826
                        :  :                          :  :* . : ::

Ce      ISVNSSTET--------FNRIGQKCFETLYKAMTDYTTSANGDIGRFVREASMRAMSTIL 945
Fh      ------------------------------------------------------------ 762
Sm      IIKNLGAHHSE-----LNATILKSTCYILLQSLSDYTMDSRGDVGSLVREVGMKCLDSYI 768
Dm      LVKTVGYG--GGIDSFAEPKNFNKVIECLLRALQEYTLDNRGDIGAWVREAAMSSLYEIV 879
Hs      ICQTVGVKA-GAPDEAVCGENVSQIYCALLGCMDDYTTDSRGDVGTWVRKAAMTSLMDLT 883
Mm      ICQTVGVNTRGPPDEVICKENISEVYAALLGCMSDYTTDSRGDVGAWVREAAMTSLMDLM 886

                                                    TFCD_C
Ce      --VDAKTEPPFLDEHVIKS-AKYMVQQSAERISRTRECACACLKSLVKC-EITGRCLPHI 1001
Fh      ----------------VEEIVVSIVQQSVEKIDRTRAAAGQAFSGILYH-EPPIRYIPHV 805
Sm      EFLVNNQYSELITSDMIEEVMTSIAQQAVEKIDRTRGVAGQVFAHLLHH-DPPIEHISHF 827
Dm      ----TTCPPDLLAPEQVHEIVVGFMQQAVEKIDRTRGLGGRLCCQLIHH-QPRIPYIREH 934
Hs      L-LLARSQPELIEAHTCERIMCCVAQQASEKIDRFRAHAASVFLTLLHFDSPPIPHVPHR 942
Mm      L-LLARTEPVLIEAHICERVMCCVAQQASEKIDRFRAHAARVFLTLLHFDSPPIPHVPHR 945
                         .     . **: *:*.* *  .      ::   .     : .

                                           TFCD_C
Ce      DLLMNIYSE----PMDFISDRTVFQLKPLLDLGSEYYEQLILGIVVSAGGLAEGTQKTAK 1057
Fh      KKVKEIFS---------------------------------------------------- 813
Sm      EELKQIFPKSDCDDMIWNSANSTFHRFTKLLDFPEYRYRLILGLIVSVGGLTELTIRCST 887
Dm      SKLLEIFPA-DADSVLWLFADHTFPLFCELLSLPDYSKRVLLGLSASIGQLTESLIKYAS 993
Hs      GELEKLFPRSDVASVNWSAPSQAFPRITQLLGLPTYRYHVLLGLVVSLGGLTESTIRHST 1002
Mm      QELESLFPRSDVATVNWNAPSQAFPLITQLLGLPTYRYHVLLGLAVSVGGLTESTVRHST 1005
          : .::

                                           TFCD_C
Ce      QLLLDHQREICENKPRFDHFLSTCADLFQRARKVNRIGNSFMQILPQIFGNLGIYEQCPE 1117
Fh      -----------------------------------NVIVPLFRFVDFLLNDPVIAGAVN- 837
Sm      SALSAYFLDHESDQLFIVEVLKIVGQILQSFRQEERIVVPLFKFLDFLLNDPIVNSTID- 946
Dm      SALFHFLRS---NPETVPRLCSEVVQIFEEHLLNERVTYPLLSFLDILIGSGTVESVLHD 1050
Hs      QSLFEYMKGIQSDPQALGSFSGTLLQIFEDNLLNERVSVPLLKTLDHVLTHGCFDIFTTE 1062
Mm      QSLFEYMKGIQKDAQVLQSFSETLLKVFEDNLLNDRVSVSLLKMLDQLLANGCFDIFTAE 1065
                                           .:   ::  :  ::    .

                   TFCD_C
Ce      TSESII-EMVDTMKTIAVRSSMMSRQRLSIDSLGELLNCGKKSTVYRSALTMILDTLNSQ 1176
Fh      ------------------------------GESFGGMLQFEG--SARKRSASLMMILMAHK 866
Sm      PNSSILLQLTESVWNETKLTKDVQRIKAAIDVFGGMLQFTG--SVRKRSLSLMMIILGSR 1004
Dm      EANPFAEDIFRLLNLEVKGYKKLYKTATSISAFCQLLQVPR--L-SKRILSKLSVFLGLQ 1107
Hs      EDHPFAVKLLALCKKEIKNSKDIQKLLSGIAVFCEMVQFPG--DVRRQALLQLCLLLCHR 1120
Mm      ENHPFCVKLLTLCKEEIKKSKDIQKLRSSIAVLCGMVQFNG--DVRKKILLQLFLLLGHP 1123
                                        :  :::         :     :   :

Ce      QPVLRKSAAERLYEHLC-------CAEESDDEVLEVLATTNWQDENDNVLKQAVA----- 1224
Fh      YPVIRKSAATKLYECLVMFDL---VEPEVMDQVTTLLTETIWESELDEIRPIRNTICEML 923
Sm      YPIIRKATATELYEGLLVYEL---CPSELLDQVSSILTETIWEGDIEAVRPIRNQLCELF 1061
Dm      HVHVRKTAATKLYEALALHGDVTEVPEENMDEILTLLSETDWTQPLVEVRPLRNQLCQLM 1167
Hs      FPLIRKTTASQVYETLLTYSDV--VGADVLDEVVTVLSDTAWDAELAVVREQRNRLCDLL 1178
Mm      FPVIRKSTASQVYEMVLTYSDL--VDAEVLDEVMSVLSDTAWDAELPVVREQRNRLCDLL 1181
           :**::* .:** :           :  *::  :*: * *      :
```

```
Ce      GISEKLIF----------------------------- 1232
Fh      GVPVPRIINPKVTPDSNTPTDQTRSVISVNGDAGDRG 960
Sm      QVPVPRATSKTQNHTSPA------------------- 1079
Dm      DIKPPVSGAAAAAALQQASADK--------------- 1189
Hs      GVPRPQLVPQPGAC----------------------- 1192
Mm      GVPRPQLVPKPIPGS---------------------- 1196
         :
```

Glutamyl-prolyl-tRNA synthetase (Eprs):

```
Ce      MMITRELVLKANREQPPYASILALAASGFSLEKSVQFSEKQQLALNLDG-EL--LSNDVE 57
Fh      -----------------------------MMESAIEWIYNN-LKKNSVDI---------- 20
Sm      ------------------------------MCVCLEAFKES-LKYHLPGL---------- 19
Dm      ----MSIKLKANLNNPPISGLATAHLINGTVPVEIVWSKEE-TSLQFPDNRLLVCHSNND 55
Hs      ---MATLSLTVNSGDPPLGALLAVEHVKDDVSISVEEGKEN--ILHV--SENVIFTDVNS 53
Mm      ---MAALCLTVNAGNPPLEALLAVEHVKGDVSISVEEGKEN--LLRV--SETVAFTDVNS 53
                                      :   :    :.    .

Ce      IARIIAQSTDAADSLLGSSIIDFAVVDDLTNFIADAVTKND-YS-----LLLGKDFPTKI 111
Fh      -----PS-EVLLSIIPKTNGTPSIHVLNWVRFVPAYLMNSKKRSWAMEQLEKSLAHATFL 74
Sm      -----NE-DSLKLALKGIAGEDDLQTYNWVRFATTYLQPQCDFVWAGKEIDKALVNSTYI 73
Dm      VLRALAR-AAPDYKLYGETAIERTQIDHWLSFSLTCE---DDISWALSFLDKSIAPVTYL 111
Hs      ILRYLAR-VATTAGLYGSNLMEHTEIDHWLEFSATKLSSCDSFTSTINELNHCLSLRTYL 112
Mm      ILRYLAR-IATTSGLYGTNLMEHTEIDHWLEFSATKLSSCDRLTSAINELNHCLSLRTYL 112
                      :            .   *                 :       * :

Ce      LDNSLTVADFAIFSVAHNNPQLKAKFSGI-----I----------DKVLKEPTLAAAHNF 156
Fh      VG---------------------------------------------------------- 76
Sm      NGSEFSLSDLAIWTFLEINPDWHKLNESYKTDGSVKSFVNLKQYYDRLITLPSVRKL--K 131
Dm      VANKLTIADFALFNEMHSRY---EFLAAKGIP------QHVQRWYDLITAQPLIQKV--L 160
Hs      VGNSLSLADLCVWATLKGNAAWQEQLKQKKAP------VHVKRWFGFLEAQQAFQSV--G 164
Mm      VGNSLTLADLCVWATLKGSAAWQEHLKQNKTL------VHVKRWFGFLEAQQAFRSV--G 164


Ce      VGLYKS-------------AAAPTATASTGKEKKKDEGKFVELPGAEKGKVVVRFPPEAS 203
Fh      ------------------------------------------------------------ 76
Sm      TEIYESRLQPRGVSVNSEPPSNTIGKTHASDMLFEKGGKFGELPGARVGEVVVRFPPEAS 191
Dm      QSLPEDAKVKR------S-PQSSKEQTPAKTGERKQEGKFVDLPGAEMGKVVVRFPPEAS 213
Hs      TKWDVS-----------------TTKARVAPEKKQDVGKFVELPGAEMGKVTVRFPPEAS 207
Mm      TKWDVS-----------------GNRATVAPDKKQDVGKFVELPGAEMGKVTVRFPPEAS 207

                                tRNA-synt_1c
Ce      GYLHIGHAKAALLNQYYQQAFEGQLIMRFDDTNPAKENAHFEHVIKEDLSMLNIVPDRWT 263
Fh      ---------DDFSVADLADTFKGRLILRFDDTNPSKEKANFEESILCDLPRIGVKWDVRS 127
Sm      GYLHIGHAKAALLNQHYRDIFKGRLILRFDDTNPDKEKECFEKSILSDLPRIGVTWDAIS 251
Dm      GYLHIGHAKAALLNQYYALAFQGTLIMRFDDTNPAKETVEFENVILGDLEQLQIKPDVFT 273
Hs      GYLHIGHAKAALLNQHYQVNFKGKLIMRFDDTNPEKEKEDFEKVILEDVAMLHIKPDQFT 267
Mm      GYLHIGHAKAALLNQHYQVNFKGKLIMRFDDTNPEKEKEDFEKVILEDVAMLHIKPDQFT 267
                   :        *:* **:******* **.  **. *  *:  : :  *  :
                                tRNA-synt_1c
Ce      HSSDHFEMLLTMCEKLLKEGKAFVDDTDTETMRNEREQRQDSRNRSNTPEKNLQLWEEMK 323
Fh      HTSDHFDLLINLCEQMLREGKAYVDNTDTETMRTERENRKPSACRENTTQQNLAWWEEMK 187
Sm      STSDHFDEMLKLCEQLIKEGKAYVDNTDTETIRVQREARQMSACRDNSIEQNLSWWGEMK 311
Dm      HTSNYFDLMLDYCVRLIKESKAYVDDTPPEQMKLEREQRVESANRSNSVEKNLSLWEEMV 333
Hs      YTSDHFETIMKYAEKLIQEGKAYVDDTPAEQMKAEREQRIDSKHRKNPIEKNLQMWEEMK 327
Mm      YTSDHFETIMKYAEKLIQEGKAYVDDTPAEQMKAEREQRTESKHRKNSVEKNLQMWEEMK 327
         :*::*: ::  . ::::*.**:**:*  * :: :** *  *  *.*  ::**  * **
                               tRNA-synt_1c
```

```
                                                  tRNA-synt_1c
Ce    KGSPKGLTCCVRMKIDMKSNNGAMRDPTIYRCKPEEHVRTGLKYKVYPTYDFTCPIVDSV 383
Fh    KGTEFGLKCCVRAKIDMNSNNGAMRDPTIYRCKPESHVRTGTKYNVYPIYDFACPVVDSV 247
Sm    KGTEQGLKCCVRAKIDMSSNNGALRDPTIYRCKIEPHVRTGSKYKVYPLYDFACPVVDSI 371
Dm    KGSEKGQKYCVRAKIDMSSPNGCMRDPTIYRCKNEPHPRTGTKYKVYPTYDFACPIVDAI 393
Hs    KGSQFGQSCCLRAKIDMSSNNGCMRDPTLYRCKIQPHPRTGNKYNVYPTYDFACPIVDSI 387
Mm    KGSQFGQSCCLRAKIDMSSNNGCMRDPTLYRCKIQPHPRTGNKYNVYPTYDFACPIVDSI 387
      **:  *  . *:* ****.* **.:****:****  :  * ***  **:***  ***:**:**::
                                                  tRNA-synt_1c
Ce    EGVTHALRTTEYHDRDDQYYFICDALGLRRPHIWEYARLNMTNTVMSKRKLTWFVDEGHV 443
Fh    EGVTHALRTSEYNDRNDQYAWFCQVLGLRCPMVIDYSRLALQNTILSKRKLTWFVEEGLV 307
Sm    EGVTHALRTSEYNDRNEQYAWICKSLGIRCPIVIDYSRLALQNTVLSKRKLAWFVEEGIV 431
Dm    ENVTHTLRTTEYHDRDDQFYWFIDALKLRKPYIWSYSRLNMTNTVLSKRKLTWFVDSGLV 453
Hs    EGVTHALRTTEYHDRDEQFYWIIEALGIRKPYIWEYSRLNLNNTVLSKRKLTWFVNEGLV 447
Mm    EGVTHALRTTEYHDRDEQFYWIIEALGIRKPYIWEYSRLNLNNTVLSKRKLTWFVNEGLV 447
      *.***:***:**:**::*: :: .  *  :*  *  :  .*:**  :  **::*****:***:.*  *
                                                  tRNA-synt_1c
Ce    EGWDDPRLPTVRGVMRRGLTVEGLKQFIVAQGGSRSVVMMEWDKIWAFNKKVIDPVAPRY 503
Fh    DGWDDPRMPTVSGILRRGMTAEGLRQFILAQGSSRSSAQMEWDKIWAFNKKVIDPIAPRF 367
Sm    DGWDDPRMPTVSGILRHGMTAEGLRQFILAQGSSRSSALMEWDKIWSFNKKIIEPVAPRT 491
Dm    DGWDDPRFPTVRGIIRRGMTVEGLKEFIIAQGSSKSVVFMNWDKIWAFNKKVIDPIAPRY 513
Hs    DGWDDPRFPTVRGVLRRGMTVEGLKQFIAAQGSSRSVVNMEWDKIWAFNKKVIDPVAPRY 507
Mm    DGWDDPRFPTVRGVLRRGMTVEGLKQFIAAQGSSRSVVNMEWDKIWAFNKKVIDPVAPRY 507
      :******:***  *::*:*:*.***::**  ***.*:*  .  *:*****:****:*:*:***
                                                  tRNA-synt_1c_C
Ce    TALDSTSPL-----------VSIELTDSISDDTSNVSLHPKNAEIGSKDVHKGKKLLLEQ 552
Fh    TGLLLDPSVPQPSPSTPIGLVPVRIHGQSEIQEKSVAVHPKNESLGHREIMVSPLVYVEH 427
Sm    TALLLDSSYMG-HNGTPPGLVRVHIHGQTGYTEKKVQIHPKNESLGYRSILLGPEVFVEQ 550
Dm    TALEKEKRVIVNVAG-------------AKVERIQVSVHPKDESLGKKTVLLGPRIYIDY 560
Hs    VALLKKEVIPVNVPE-------------AQEEMKEVAKHPKNPEVGLKPVWYSPKVFIEG 554
Mm    VALLKKEVVPVNVLD-------------AQEEMKEVARHPKNPDVGLKPVWYSPKVFIEG 554
      ..*                                  .*   ***:  .:*  :  :   .   :  ::
                                                  tRNA-synt_1c_C
Ce    VDAAALKEGEIVTFVNWGNIKIGKIEKKG-AVITKISATLQLDNTDYKKTTKVTWLGDVK 611
Fh    ADATCFKEGENVTFINWGNCRIVKIHRSE-AYITAVDGELNLQDTDYKKTLKITWLPELE 486
Sm    ADALCFKKGENVTFINWGNLRIMDIHRKG-QLIVSIDACLNLEDTDYKKTLKITWLTDPK 609
Dm    VDAEALKEGENATFINWGNILIRKVNKDASGNITSVDAALNLENKDFKKTLKLTWLAVED 620
Hs    ADAETFSEGEMVTFINWGNLNITKIHKNADGKIISLDAKLNLENKDYKKTTKVTWLAETT 614
Mm    ADAETFSEGEMVTFINWGNINITKIHKNADGKITSLDAKLNLENKDYKKTTKITWLAEST 614
      .**   :.:**  .**:****   *  .:.:.      *    :..  *:*::.*:***  *:***
                                                  tRNA-synt_1c_C
Ce    AEAGKTIPVVTADYDHIISKAIIGKDEDWKQFINFDSVHYTKMVGEPAIKNVKKGDIIQI 671
Fh    GKSPALCPVTCLIYDHLLTKGILGKDEDFKPYVNRNSKVEQCLLGDPDLRNLKKGEIIQL 546
Sm    DSHLPLIPVSCLTYDNLLNKAILGKDDDFKLYVNKCSKKEQCLLGDPDLRHVKKGDIIQI 669
Dm    D-PSAYPPTFCVYFDNIISKAVLGKDEDFKQFIGHKTRDEVPMLGDPELKKCKKGDIIQL 679
Hs    H-AL-PIPVICVTYEHLITKPVLGKDEDFKQYVNKNSKHEELMLGDPCLKDLKKGDIIQL 672
Mm    H-AL-SIPAVCVTYEHLITKPVLGKDEDFKQYINKDSKHEELMLGDPCLKDLKKGDIIQL 672
                *.     :::::.*  ::***:*:*  ::.   :      ::*:*  ::.  ***:***:
      tRNA-synt_1c_C
Ce    QRKGFYIVDQPYNPKSELSGVETPLLLIAIPDGHTGKEAEKPPKGA-PVS------AAAS 724
Fh    QRRGYYIVDVPYEAKCKATGFESPCVLIQIPEGTMSKTTTDTAGST-TSQAKK---GGSV 602
Sm    QRRGFFICDAPYESACLATGHESPCVLINIPDGSSKDDNAAI--NS-KVESSK---SGSV 723
Dm    QRRGFFKVDVAYAPPSGYTNVPSPIVLFSIPDGHTKDVPTSGLKVNAPDAKATKKASSPV 739
Hs    QRRGFFICDQPYEPVSPYSCKEAPCVLIYIPDGHTKEMPTSGSKEKTKVEATKNETSAPF 732
Mm    QRRGFFICDQPYEPVSPYSCREAPCILIYIPDGHTKEMPTSGSKEKTKVEISKKETSSAP 732
      **:*::  *  *    .  :   :*  :*:  **:*   .                      ..

Ce    SGGSDALQLYNSIEQQGGIVRDLKTKDAKSQATKDAIAKLLDLKKKYKELTGSDHKPGAP 784
Fh    KTS--------------------------------------------------------- 605
Sm    KTN--------------------------------------------------------- 726
Dm    SSSGQASELDSQISQQGDLVRDLKSKKAAKDQIDVAVKKLLALKADYKSATGKDWKPGQT 799
Hs    KER--------------------------------------------------------- 735
Mm    KER--------------------------------------------------------- 735
```

```
        .

Ce      PSAPAPTTPS-SSESNSALNIYNLIEAQGLLVRELKGKDAKSQATKDAIAKLLELKKQYK 843
Fh      -----------------------AKQPKAEMTEEEAAK--------ALERQ------RKK 628
Sm      -----------------------KTVKIENLTPEEAAK--------AAEQQ------RRK 749
Dm      SASSAPVPAASSSSANDAVSVNASIVKQGDLVRDLKGKKASKPEIDAAVKTLLELKAQYK 859
Hs      ---PTPSLNNNCTTSEDSLVLYNRVAVQGDVVRELKAKKAPKEDVDAAVKQLLSLKAEYK 792
Mm      ---PAPAVSSTCATAEDSSVLYSRVAVQGDVVRELKAKKAPKEDIDAAVKQLLTLKAEYK 792
                                      :. :  .*                   . *

Ce      EVSGSDHKPGVPPAASVASAPAPAPAAGSTNALDIYHQIESQGALVRELKGKDAKSQATK 903
Fh      EEKKEARKEGRAKAKQAK------------------------------------------ 646
Sm      EEKKEARKEGKLKAKQQQ------------------------------------------ 767
Dm      TLTGQDWKPGTVPTTAAPSASAAPSVGVNDSVAQILSQITAQGDKVRELKSAKADKATVD 919
Hs      EKTGQEYKPGNPPAEIGQNISSNSSASILE-SKSLYDEVAAQGEVVRKLKAEKSPKAKIN 851
Mm      EKTGQEYKPGNPSAAAVQTVSTKSSSNTVE-STSLYNKVAAQGEVVRKLKAEKAPKAKVT 851
          . .  * *   :

Ce      DAIAKLLALKKQYKEVTGSDHKPGVVPVSAPA------------PTPLAGGADIAGQIEA 951
Fh      ETTECSEM------QSTS--------------------MKTQPEPASS----NEQPQNKK 676
Sm      SSENKLRT------TVVG--------------------HETINKPTSD----KLMYKQSN 797
Dm      AAVKTLLSLKADYKAATGSDWKPGTTAPAPAAAPVKVKQEKNPDPASVLTVNTLLNKIAQ 979
Hs      EAVECLLSLKAQYKEKTGKEYIPGQPPLSQSSDSSPT-RNSEPAGLETPEAKVLFDKVAS 910
Mm      EAVECLLSLKAEYKEKTGKDYVPGQPPASQNSHSNPV-SNAQPAGAEKPEAKVLFDRVAC 910
         :              ..                                      :

Ce      QGVLVRDLKNKDSKSQETKDAIAKLLELKKNYKEVTGSDYKPGPAPAAAPAKVTVPAPSV 1011
Fh      QEQ--------------------------------------------------------- 679
Sm      QSD--------------------------------------------------------- 800
Dm      QGDKIRQLKSAKSEKSLVEAEVKLLLALKTDYKSLTGQEWKPGTVAPAP---TTVNVIDL 1036
Hs      QGEVVR------------------------------------------------------ 916
Mm      QGEVVR------------------------------------------------------ 916
        *

Ce      SGGDTL------SKQIDDQALLVRELKMKDAKSQETKDAIAKLLQLKKQYKDATGSDYKP 1065
Fh      ----------------------------------CVKPTVEA-------------VSRPP 692
Sm      ----------------------------------SVKCE-----------------QLK- 808
Dm      TGGDSGSDVGSVLSKIQAQGDKIRKLKSEKAAKNVIDPEVKTLLALKGEYKTLSGKDWTP 1096
Hs      ------------------------KLKTEKAPKDQVDIAVQELLQLKAQYKSLIGVEYKP 952
Mm      ------------------------KLKAEKASKDQVDSAVQELLQLKAQYKSLTGIEYKP 952
                                            .                   .

Ce      APAQLAASAAA--APAPAPAFDEAALLKEIEEQGAVVRDAKSKDPKSQDSADAINKLLAL 1123
Fh      IVA-------ESCSAKSI----------------------K------------------- 704
Sm      -----------PVSPKVI----------------------V------------------- 816
Dm      DAKSEPAVVKKEASPVSMASPAKDELTQEINAQGEKVRAAKGNKAAKEVIDAEVAKLLAL 1156
Hs      VSATGAEDKDKKK--------------KEKENKSE-----KQNKPQKQ------------ 981
Mm      VSATGAEDKDKKK--------------KEKENKSE-----KQNKPQKQ------------ 981

Ce      KANFKKATGKDFPAPSNAQSKKGKKK---------------------------------- 1149
Fh      --------------------------------------IPKSDQVELSKKEGAKKQTRLA 726
Sm      --------------------------------------KNEKQKESLLKMSGTKKQSKLA 838
Dm      KAKYKEVTGTDFPVAGRGGGGGGGSAKKAPKEAQPKPAKPVKKEPAADASGAVKKQTRLG 1216
Hs      ----NDGQRKD-PSKNQGGGLSS---------------------SGAGEGQGPKKQTRLG 1015
Mm      ----NDGQGKD-SSKSQGSGLSS---------------------GGAGEGQGPKKQTRLG 1015

Ce      ------------------------------------------------------------ 1149
Fh      LEATKESEFSEWYSQLITKAELLEYYDISGCYIIRPWAYSLWQTIQRFLDERLHAMGVEN 786
Sm      IEASKETDFSDWYSELIIKSELLDYYDISGCYILRPWAYHMWQSIQCFMDEKLKVMGIEN 898
Dm      LEATKEDNLPDWYSQVITKGEMIEYYDVSGCYILRQWSFAIWKAIKTWFDAEITRMGVKE 1276
```

```
Hs   LEAKKEENLADWYSQVITKSEMIEYHDISGCYILRPWAYAIWEAIKDFFDAEIKKLGVEN 1075
Mm   LEAKKEENLAEWYSQVITKSEMIEYYDVSGCYILRPWSYSIWESIKDFFDAEIKKLGVEN 1075

                                                tRNA-synt_2b
Ce   ------------------------------------------------------------ 1149
Fh   AYFPMFVSKGALEREKNHVSDFAPEVAWVTKSGDSDLAEPVAVRPTSETIMYPAFAKWIQ 846
Sm   AYFPMFVSKSALEREKNHVTDFAPEVAWVTKSGDTDLAEPIAIRPTSETIMYPIFAKWIQ 958
Dm   CYFPIFVSKAVLEKEKTHIADFAPEVAWVTKSGDSDLAEPIAVRPTSETVMYPAYAKWVQ 1336
Hs   CYFPMFVSQSALEKEKTHVADFAPEVAWVTRSGKTELAEPIAIRPTSETVMYPAYAKWVQ 1135
Mm   CYFPIFVSQAALEKEKNHIEDFAPEVAWVTRSGKTELAEPIAIRPTSETVMYPAYAKWVQ 1135

                              tRNA-synt_2b
Ce   ------------------------------------------------------------ 1149
Fh   SHRDLPLRLNQWSNVVRWEFKHPQPFLRTREFLWQEGHTAYAEKPDAEAEVLAILDLYAQ 906
Sm   SHRDLPLRINQWSNVVRWEFKHPQPFLRTREFLWQEGHTAFAEKADAEAEVRVILDLYAE 1018
Dm   SYRDLPIRLNQWNNVVRWEFKQPTPFLRTREFLWQEGHTAFADKEEAAKEVLDILDLYAL 1396
Hs   SHRDLPIKLNQWCNVVRWEFKHPQPFLRTREFLWQEGHSAFATMEEAAEEVLQILDLYAQ 1195
Mm   SHRDLPVRLNQWCNVVRWEFKHPQPFLRTREFLWQEGHSAFATFEEAADEVLQILELYAR 1195

                              tRNA-synt_2b
Ce   ------------------------------------------------------------ 1149
Fh   VYQDLLAVPVIKGRKTEREKFAGADYTTTIEAYISGTGRAIQGATSHHLGQNFSRMFEVT 966
Sm   VYEYLLAVPVVKGRKTEREKFAGADYTTTVEIYIDGNGRAIQGATSHHLGQNFSRMFDVT 1078
Dm   VYTHLLAIPVVKGRKTEKEKFAGGDYTTTVEAFISASGRAIQGATSHHLGQNFSKMFEIV 1456
Hs   VYEELLAIPVVKGRKTEKEKFAGGDYTTTIEAFISASGRAIQGGTSHHLGQNFSKMFEIV 1255
Mm   VYEELLAIPVVRGRKTEKEKFAGGDYTTTIEAFISASGRAIQGATSHHLGQNFSKMCEIV 1255

            tRNA-synt_2b                           HGTP_anticodon
Ce   ------------------------------------------------------------ 1149
Fh   YDHPVT-GKPAFVYQNSWGLTTRTLGVLVMVHGDSKGLVLPPRIAPHQIVVVPCGITNKS 1025
Sm   YDHPVT-GKPAYVYQNSWGLTTRTLGVLIMVHSDDKGLVLPPRVAPYQIVIVPCGITAKT 1137
Dm   YEDPETQ-QKKYVYQNSWGITTRTIGVMIMVHADNQGLVLPPHVACIQAIVVPCGITVNT 1515
Hs   FEDPKIPGEKQFAYQNSWGLTTRTIGVMTMVHGDNMGLVLPPRVACVQVVIIPCGITNAL 1315
Mm   FEDPKTPGEKQFAYQCSWGLTTRTIGVMVMVHGDNMGLVLPPRVASVQVVVIPCGITNAL 1315

                              HGTP_anticodon
Ce   ------------------------------------------------------------ 1149
Fh   SQEDRENLLSYALLVTKTLKKDSSQFRVHCDDRTHVSPGWKFNHWEMKGVPVRLEVGPQE 1085
Sm   TVQERETLLSAAHSVFELLNKSNKQFRVHCDDRDNVSPGWKFNHWELKGVPIRLEIGPQE 1197
Dm   KDDERAQLLDACKALEKRLV--GGGVRCEGDYRDNYSPGWKFNHWELKGVPLRLEVGPKD 1573
Hs   SEEDKEALIAKCNDYRRRLL--SVNIRVRADLRDNYSPGWKFNHWELKGVPIRLEVGPRD 1373
Mm   SEEDREALMAKCNEYRRRLL--GANIRVRVDLRDNYSPGWKFNHWELKGVPVRLEVGPRD 1373

            HGTP_anticodon
Ce   ------------------------------------------------------------ 1149
Fh   MAKQSTCLVLRHNGIKLTVPLADICARMPQILEDIHNDLFRKATGELAAHVVQVGSLDQL 1145
Sm   VADRKVCLVLRHTGERVNVPMDSLINELPRILDDIHNAMFNKATKSLASHVMLVNDMNEL 1257
Dm   LKAQQLVAVRRDTVEKITIPLADVEKKIPALLETIHESMLNKAQEDMTSHTKKVTNWTDF 1633
Hs   MKSCQFVAVRRDTGEKLTVAENEAETKLQAILEDIQVTLFTRASEDLKTHMVVANTMEDF 1433
Mm   MKSCQFVAVRRDTGEKLTIAEKEAEAKLEKVLEDIQLNLFTRASEDLKTHMVVSNTLEDF 1433

                              ProRS-C_1
Ce   ------------------------------------------------------------ 1149
Fh   CSALDAKSLALAPFCGDSDCEEIIRRESARNVVVEPGAPSMGAKSLCIPFSCPFNDKLVC 1205
Sm   CTALDQKSLALAPFCCDRDCEEVIKHESARNVIVEPGAPAMGAKSLCIPFACDFNPTLVC 1317
Dm   CGFLEQKNILLAPFCGEISCEDKIKADSARGEEAEPGAPAMGAKSLCIPFDQPAP----- 1688
Hs   QKILDSGKIVQIPFCGEIDCEDWIKKTTARDQDLEPGAPSMGAKSLCIPFKPLCE----- 1488
Mm   QKVLDAGKVAQIPFCGEIDCEDWIKKMTARDQDVEPGAPSMGAKSLCIPFNPLCE----- 1488

            ProRS-C_1
Ce   ------------------------------ 1149
Fh   GSPATGTKCFNQPHCSRVATAYTLFGRSY 1234
Sm   GSPAPDTPCFNRQYCSRKAISYTLFGRSY 1346
Dm   --IAASDKCIN-PSCTNKPKFYTLFGRSY 1714
```

```
Hs        --LQPGAKCVC-G--KNPAKYYTLFGRSY 1512
Mm        --LQPGAMCVC-G--KNPAKFYTLFGRSY 1512
```

Actin, cytoplasmic 1 (Actb):

```
                                        ACTIN
Mm        MVDDELTALVVDNGSGMCKAGFGGDDAPRAVFPSMVGRPRHQGVMVGMGQKDCYVGDEAQ 60
Hs        -MDDDIAALVVDNGSGMCKAGFAGDDAPRAVFPSIVGRPRHQGVMVGMGQKDSYVGDEAQ 59
Dm        MCDEEVAALVVDNGSGMCKAGFAGDDAPRAVFPSIVGRPRHQGVMVGMGQKDSYVGDEAQ 60
Ce        MCDDDVAALVVDNGSGMCKAGFAGDDAPRAVFPSIVGRPRHQGVMVGMGQKDSYVGDEAQ 60
Fh        MGDEDVAALVIDNGSGMCKAGFAGDDAPRAVFPSIVGRPRHQGVMVGMGQKDSYVGDEAQ 60
Sm        MGDEDVAALVIDNGSGMCKAGFAGDDAPRAVFPSIVGRPRHQGVMVGMGQKDSYVGDEAQ 60
            *::::***:***********.***********:*****************.*******
                                        ACTIN
Mm        SKRGILTLKYPIEHGVVTNWDDMEKIWYHTFYNELRVAPDEHPILLTEAPLNPKINREKM 120
Hs        SKRGILTLKYPIEHGIVTNWDDMEKIWHHTFYNELRVAPEEHPVLLTEAPLNPKANREKM 119
Dm        SKRGILTLKYPIEHGIVTNWDDMEKIWHHTFYNELRVAPEEHPVLLTEAPLNPKANREKM 120
Ce        SKRGILTLKYPIEHGIVTNWDDMEKIWHHTFYNELRVAPEEHPVLLTEAPLNPKANREKM 120
Fh        SKRGILTLKYPIEHGIVTNWDDMEKIWHHTFYNELRVAPEEHPVLLTEAPLNPKANREKM 120
Sm        SKRGILTLKYPIEHGIVTNWDDMEKIWHHTFYNELRVAPEEHPVLLTEAPLNPKANREKM 120
          ***************:***********:***********:***:********** *****
                                        ACTIN
Mm        TQIMFEAFNTPAMYVAIQAVLSLYASGRTTGIVMDSGDGVTHTVPIYEGYALPHAILRLD 180
Hs        TQIMFETFNTPAMYVAIQAVLSLYASGRTTGIVMDSGDGVTHTVPIYEGYALPHAILRLD 179
Dm        TQIMFETFNTPAMYVAIQAVLSLYASGRTTGIVLDSGDGVSHTVPIYEGYALPHAILRLD 180
Ce        TQIMFETFNTPAMYVAIQAVLSLYASGRTTGIVLDSGDGVTHTVPIYEGYALPHAILRLD 180
Fh        TQIMFETFNAPAMYVAIQAVLSLYASGRTTGIVLDSGDGVTHTVPIYEGYALPHAILRLD 180
Sm        TQIMFETFNVPAMYVAIQAVLSLYASGRTTGIVLDSGDGVTHTVPIYEGYALPHAILRLD 180
          ******:**.***********************:******:*******************
                                        ACTIN
Mm        LAGRDLTDYLMKILTERGYNFTTTAEREIVRDVKEKLCYVALDFEQEMVTAAASSSLERS 240
Hs        LAGRDLTDYLMKILTERGYSFTTTAEREIVRDIKEKLCYVALDFEQEMATAASSSSLEKS 239
Dm        LAGRDLTDYLMKILTERGYSFTTTAEREIVRDIKEKLCYVALDFEQEMATAASSSSLEKS 240
Ce        LAGRDLTDYLMKILTERGYSFTTTAEREIVRDIKEKLCYVALDFEQEMATAASSSSLEKS 240
Fh        LAGRDLTDHLMKILTERGYSFTTTAEREIVRDIKEKLCYVALDFEQEMGTASASSSLEKS 240
Sm        LAGRDLTDYMMKILTERGYSFTTTAEREIVRDIKEKLCYVALDFEQEMGTAASSSALEKS 240
          ********::*********.************:*************** **::**:**:*
                                        ACTIN
Mm        YELPDGQVITIGNERFRCPEAIFQPSFLGIESRGIHETTFNSIMKCDVDIRKDLFANTVL 300
Hs        YELPDGQVITIGNERFRCPEALFQPSFLGMESCGIHETTFNSIMKCDVDIRKDLYANTVL 299
Dm        YELPDGQVITIGNERFRCPEALFQPSFLGMEACGIHETTYNSIMKCDVDIRKDLYANTVL 300
Ce        YELPDGQVITVGNERFRCPEALFQPSFLGMESAGIHETSYNSIMKCDIDIRKDLYANTVL 300
Fh        YELPDGQVITIGNERFRCPEALFQPSFLGMESAGIHETTYNSIMKCDVDIRKDLYANTVL 300
Sm        YELPDGQVITIGNERFRCPEALFQPSFLGMEASGIHETTYNSIMKCDVDIRKDLYSNTVL 300
          **********:**********:*******:*: *****::*******:******::****
                                        ACTIN
Mm        SGGSTMYPGIADRMQKEIVTLAPSTMKIKIIAPPERKYSVWIGGSILASLSTFQQMWISK 360
Hs        SGGTTMYPGIADRMQKEITALAPSTMKIKIIAPPERKYSVWIGGSILASLSTFQQMWISK 359
Dm        SGGTTMYPGIADRMQKEITALAPSTMKIKIIAPPERKYSVWIGGSILASLSTFQQMWISK 360
Ce        SGGTTMYPGIADRMQKEITALAPSTMKIKIIAPPERKYSVWIGGSILASLSTFQQMWISK 360
Fh        SGGSTMYPGIADRMQKEITALAPSTMKIKIVAPPERKYSVWIGGSILASLSTFQQMWISK 360
Sm        SGGSTMYPGIADRMQKEISALAPSTMKIKIVAPPERKYSVWIGGSILASLSTFQQMWISK 360
          ***:************** :**********:*****************************
                ACTIN
Mm        QEYDEAGPPIVHRKCF        376
Hs        QEYDESGPSIVHRKCF        375
Dm        QEYDESGPSIVHRKCF        376
Ce        QEYDESGPSIVHRKCF        376
Fh        QEYDESGPGIVHRKCF        376
Sm        QEYDESGPSIVHRKCF        376
          *****:** *******
```

U2 small nuclear ribonucleoprotein A (Snrpa1):

```
Ce      MVRLTTELFAERPQFVNSVNMREINLRGQKIPVIENMGVTRDQFDVIDLTDNDIRKLDNF 60
Fh      MVRLTAELIENAPQFTNAIQDRELDLHGYKFPAIENMGSTLDQFDTIDLSGNEIRKLDGF 60
Sm      MVRITSEIVENAPQFTNAIKDRELSLRSYKFPAIENMGCTLDQFDTIDLSDNEIRKLDGF 60
Hs      MVKLTAELIEQAAQYTNAVRDRELDLRGYKIPVIENLGATLDQFDAIDFSDNEIRKLDGF 60
Mm      MVKLTAELIEQAAQYTNAVRDRELDLRGYKIPVIENLGATLDQFDAIDFSDNEIRKLDGF 60
Dm      MVKLTPELINQSMQYINPCRERELDLRGYKIPQIENLGATLDQFDTIDLSDNDLRKLDNL 60
        **::* *:. :  *: *  . **:.*:. *:* ***:* * ****.**::.*::****.:
                                      LRR_8
Ce      PTFSRLNTLYLHNNRINYIAPDIATKLPNLKTLALTNNNICELGDIEPLAECKKLEYVTF 120
Fh      PLLKRLKALILNNNKIVRIAEDLGQQLPYLNTLILTSNSFTELRELDPLATCDKLTFLTL 120
Sm      PMLKRLKSLILTNNKIARIAEDLGQHLPNLLTLILTSNYLSDLKDLDPLSSCDKLNFLSL 120
Hs      PLLRRLKTLLVNNNRICRIGEGLDQALPCLTELILTNNSLVELGDLDPLASLKSLTYLSI 120
Mm      PLLRRLKTLLVNNNRICRIGEGLDQALPCLTELILTNNSLVELGDLDPLASLKSLTYLSI 120
Dm      PHLPRLKCLLLNNNRILRISEGLEEAVPNLGSIILTGNNLQELSDLEPLVGFTKLETICL 120
        * : **: * : **:*  *. .:   :* *  : **.* : :* :::**    .*  : :
         LRR_8          LRRcap
Ce      IGNPITHKDNYRMYMIYKLPTVRVIDFNRVRLTEREAAKKMFKGKSGKKARDAIQK---- 176
Fh      THCPVTMRANYRLYVISLLPSLRFLDYKRVTNSERKLANSMFKRIPAMASSASGVKGAPG 180
Sm      LHCPVTMRANYRLYVISRVASLRFLDYRRVTQAERKLARSMFKRLPALSNSVNNLKAPYQ 180
Hs      LRNPVTNKKHYRLYVIYKVPQVRVLDFQKVKLKERQEAEKMFKGKRGAQLAK-------- 172
Mm      LRNPVTNKKHYRLYVIYKVPQVRVLDFQKVKLKERQEAEKMFKGKRGAQLAK-------- 172
Dm      LINPVSTKPNYREYMAYKFPQLRLLDFRKIKQKDRQAAQEFFRTKQGKDVLK-------- 172
           *:: : :** *:   .  :*.:*:.::   :*: *..:*:   .

Ce      ---------------SVHTEDPSEIEPN-------------------------------- 189
Fh      LRNGIRSQNPAGGTTTVKTFIPGAPLYTNAGVDKEN------------------TA-SID 221
Sm      KANSNRIENSIPAAGVVKTFIPGAPINSNNLLPPGTEPTRSSNEQIHHGEGKENNAAALN 240
Hs      -D----------IARRSKTFNPGAGLPT-------------------------------- 189
Mm      -D----------IARRSKTFNPGAGLPT-------------------------------- 189
Dm      -E----------ISRKSKMSAAAAIAAEA-------------------GNG--------- 193
                         :    .

Ce      -------------------------------ENSSGGGARLTDEDREKIKEAIKNAKSLS 218
Fh      ----------ARGSTETKDISSMPPPPPAAAVQMGNKRPGGSAQDLYAIQEAIKRARTMD 271
Sm      TSVETDQTESTKSDTLVVSSADTPMPPPSTPVTTGNKRPVASSQDLFAIQEAIKRARTMD 300
Hs      ---------------------------------DK-KKGGPSPGDVEAIKNAIANASTLA 215
Mm      ---------------------------------DK-KKGGPSAGDVEAIKNAIANASTLA 215
Dm      ------------------------------KGRGSEGGRLANPQDMQRIREAIKRASSLA 223
                                                 .  *   *::** .* ::

Ce      EVNYLQSILASGKVPEK--GWNRQMDQNGADGEAMES----------------------- 253
Fh      EVDRLHQLLSSGQFAGFAIQWQQQLRLQQQQNQQQRQQQQEQQEQEQQRKAEQEQETSAM 331
Sm      EVERLHQLLSSGQFAGFAAQWQKQLRQQQQQKSSQ------------------------- 335
Hs      EVERLKGLLQSGQIPGRERRSGPT------------D-----------------DGEEEM 246
Mm      EVERLKGLLQSGQIPGRERRSGPS------------D-----------------EGEEEI 246
Dm      EVERLSQILQSGQLPDKFQHEMEAVAQNGAGH---NG----------------SGAVAM 263
        **: *  :* **:.

Ce      ---------------------------------------------------- 253
Fh      DEEQTEQVQQPETMVQQNELLQHEESTNLEMATQDNIVDQQIHSMEVTQPVE 383
Sm      ---------------------------------------------------- 335
Hs      EEDTVTNGS------------------------------------------- 255
Mm      EDDTVTNGS------------------------------------------- 255
Dm      EY-------------------------------------------------- 265
```

Leucine zipper and EF-hand containing transmembrane protein 1 (Letm1):

```
Fh      ML---------------------VILRNVARPHRV--------RVLCPRRRER--AAGS 28
Sm      ------------------------------------------------------------ 0
Ce      ------------------------------------------------------------ 0
Dm      MNALLRHKGRNLRTSHLAQNVYKRFLKSNCCACSSVNVTDEPAKEDELPRRSAS-TSVLE 59
Hs      MA---------------------SILLRSCRGRAPARLPPP--PRYTVPRGSPGDPAHLS 37
Mm      MA---------------------SILLRSCRGRGPARLAP---PRAASPRGSLRDRACLS 36


Fh      SFQNAGHYIHNRAVFG---PS-----VFRTQSTSQWNLSLRSLH-LNSSLWSG----TET 75
Sm      ------------------------------------------------------------ 0
Ce      ---------------------MSLRYASNAYHIGRYAQRLFATGQRNRVFHQYIASTSLQ 39
Dm      LSRSLGTYRRF-------QPH-ANY----GYDYSGYGFRHLH---TSRTLL--------E 96
Hs      CASTLGLRNCLNVPFGCCTPIHPVYTSSRGDHLGCWALRPECLRIVSRAPW--------T 89
Mm      CTRTLGLTSRESVLSRCCTPAHPVYLCFKGEPLSCWTQRPECQGTAARTTW--------T 88


Fh      TNSLRVGDSR-----P-----EKYHPSLFLVDNPPPETSS-KKPPAKS---------ESS 115
Sm      ------------------------------------------------------------ 0
Ce      TTSLRYAST----------------------------DRSKVEYTLKMLREDVQKQDEE- 70
Dm      TSSSKIDATVKKLKNQQKEKVEEIMKEVANGQAAAVRASSAATATASSEKGQNASATAGS 156
Hs      STSVGFVAV-----GPQCLPVRGWH------SSRPVRDDSVVEKSLKSLKDKNKKLEEGG 138
Mm      PASARLVVT-----GPQYLPVRGWH------SSSPLGEDSVIEKSLKSLKDKNKKLEEGG 137

                                                Letm1
Fh      SSALSETPEDPEKNESNTEQKKLSMWQKVKKEVAHYYHGFRLLGLEVKIASGICIRLLCG 175
Sm      ------------------------------------------------------------ 0
Ce      ----AQKVLSLMKSDKDVATVKPPLKDRIIHELKHYYHGFRLLALETRVSAKYLWTVLRG 126
Dm      TSATASTTSLAKTADKSVAKPKKPLRTRIWDELVHYYHGFRLLFIDVAICSKLLWRVLNG 216
Hs      PV----------YSPPAEVVVKKSLGQRVLDELKHYYHGFRLLWIDTKIAARMLWRILNG 188
Mm      PV----------YSPPAQVVVRKSLGQKVLDELRHYYHGFRLLWIDTKIAARMLWRILNG 187

                                   Letm1
Fh      NSLTRRERKQLVRTVADIIRLVPFAVFIIVPFMEFLLPFYLKFFPFMLPSTFKYKSTEAE 235
Sm      ----------------------------------------------MLPSTFKDKSTEAN 14
Ce      ATLSRRERQQLVRTVSDLFRLVPFSFFIIVPFMELALPIFIKLFPGMLPSTFQESSKEEE 186
Dm      KTLTRRENKQLQRTTSDLFRLIPFSVFIIVPFMELLLPLFIKFFPGMLPSTFQTSTDRQE 276
Hs      HSLTRRERRQFLRICADLFRLVPFLVFVVVPFMEFLLPVAVKLFPNMLPSTFETQSLKEE 248
Mm      HTLTRRERRQFLRICADLFRLVPFLVFVVVPFMEFLLPVVVKLFPNMLPSTFETQSIKEE 247
                                                      ******:  .:  .  :
                                   Letm1
Fh      KIRQRVKAKLEMTRFLQETLHHTTGAVSEGEK-SPSFEQFQGFLKKVQESGQFLSDEEIT 294
Sm      AIQQRLKAKLELTRFLQETLMQTAGALKSSSD-APTVAEFQEFIKKVQKSGEQAHAKDIT 73
Ce      KWRKQVKLRVEMAKFLQDTIEEIGLERKTRNKESTRSLEFALFIKKVRNEGGYVSNEELL 246
Dm      KLRQSLSVRLEVAKFLQQTLDQMPVQHKEHSSE--EAKQFEAFFTKIRNPTEPVSNDEII 334
Hs      RLKKELRVKLELAKFLQDTIEEMALKNKAAKGS--ATKDFSVFFQKIRETGERPSNEEIM 306
Mm      RLKKELRVKLELAKFLQDTIEEMALKNKAAKGN--ATKDFSAFFQKIRETGERPSNEEIM 305
          :: :  ::*:::***:*:  .       .   .       :*   *:  *:::         .::
                                    Letm1
Fh      RFSKLFEDQITLDSLELNQLKMLCRLLSLPTMGPSNLLRFQLQMRVRQLKAEDRLLA-KE 353
Sm      RFSKLFEDQVTLDSLDNKQLRMLCRLLSLPTIGPSHLLRFQIWIRVRQLKAEDKLIA-NE 132
Ce      KFSKLFEDEITLDNLSMGQLRSLCRLMSINSLGSPEILRFQLNMKIRELKADDKQIAAEG 306
Dm      KFAKRFDDEITLDSLSREQLAALCRVLELNTIGTTTLLRFQLRLKLRSLATDDRVIA-RE 393
Hs      RFSKLFEDELTLDNLTRPQLVALCKLLELQSIGTNNFLRFQLTMRLRSIKADDKLIA-EE 365
Mm      RFSKLFEDELTLDNLTRPQLVALCKLLELQSIGTNNFLRFQLTMRLRSIKADDKLIS-EE 364
        :*:* *:*::***.*   **   **:::.: ::*   :****: :::*.: ::*: ::  .
                              Letm1
Fh      GVNTIPAWELQTLCQDRGMRSVGLTEERLRNQLAQWLNLHLEKNVPVTLLLFSRALHVSK 413
Sm      GVDQIPPWELQSLCQERGMRSVGLPKEKLQSQLSEWLDLHLEKNVPITLLLFSRALHVTQ 192
Ce      GVDALSSIDLQSACRARGMRAIGVSEERLKEQLVQWLELSLNDKVPPALLLLSRTLYLPE 366
Dm      GVDSLDLLELQQACKARGMRAYGLTEERLRFQLKEWIDLSLNEQVPPTLLLLSRTMLISD 453
Hs      GVDSLNVKELQAACRARGMRALGVTEDRLRGQLKQWLDLHLHQEIPTSLLILSRAMYLPD 425
Mm      GVDSLTVKELQAACRARGMRALGVTEDRLKGQLKQWLDLHLHHEIPTSLLILSRAMYLPD 424
        **: :    :**   *: ****: *: :::*: ** :*::* *..::* :**::**:: :  .
```

```
Fh      ASTAELPLKEAIAQLPTAASEEATARVLESALPTELDPRTKIELLRKEQANIKAARIQRK 473
Sm      ALVDQNPLQQAIAQLPPSASSEAVARVLETTPHDELDPVTKIKVLREEQDSIKAERVQRK 252
Ce      EVSFPDRLKAIVQNLPDGLAETTKQKLTEM-EGGQIDHKARIELIRSIESAIANEKKDEE 425
Dm      DSITTDKLKETIRVLPDAVGAHTRHAIGES-EG-KVDNKTKIEIIKEEERKIREEREEER 511
Hs      TLSPADQLKSTLQTLPEIVAKEAQVKVAEV-EGEQVDNKAKLEATLQEEAAIQQEHREK- 483
Mm      TLSPADQLKSTLQTLPEIVAKEAQVKVAEV-EGEKVDNKAKLEATLQEEAAIQQEHLEE- 482
               *:  :  **   .  :   : *     ::*  ::::   . :  *   : :.

Fh      QELAEQKKAAASELDAKSAREQKLAPAAEDLVDKAPVLKGLPKEELENVKEAPSVSVVST 533
Sm      QELIEKQKSEKGKTGL-------------LEGDQAPLLKGLKNEELDKVTPVLTDSAAFM 299
Ce      KKKKAAEEALKSKEEAEKAKVQE------------K---EK----------LVDVAAQAI 460
Dm      EETIAKRSAIKEEIP-----------------------------------AP-----YV 530
Hs      --ELQKRSEVAKDFEPERV-VAA------------P---QRP-----GTEPQPEMPDTVL 520
Mm      ---LKRASEAVKDIQPEVAEATL------------P---GRP-----GPEPQPPVDDVIL 519
               .    .

Fh      TEEP-LALTIEGKK-SQTAVAAQATPTESTSSKELQLKE-ALKKVLAAKVTKEEEPEITV 590
Sm      SENTEIQPEIRKHVQNIPPIHAVQTAFDGKSKDDSHIDSLSMKPEKPKTSDTEEVTEISA 359
Ce      VH--------ELKKDIYDAVASTSSTTEA--------KEPAAKPSEAKEEKK-EDITVDK 503
Dm      FA--------EKLSG-------SQDLLDH--------KEQSSVSETDKGISS-TDVQLLS 566
Hs      QS--------ETLKD-------TAPVLEG---------------LKEEEITK-EEIDILS 549
Mm      PS--------EVLTD-------TAPVLEG---------------LKGEEITK-EEIDILS 548
                  .                :                       .     :

Fh      VDLAQIESAIAESSGALHEEAMEGLKEEVAETAT---KHTASLVSADRTVDKRTTKAAQR 647
Sm      GDLAEIHSAIAESTPHLDSETIDGLKEQVAKTAKLQQKRQAELLASEEVTDKRKSKAALH 419
Ce      KDLSHIEEIIVGGPIKEAKHDILGLREKVLEHKEDLMEINS------LDGAFAETKIAKR 557
Dm      EALKTLS---SDKQLVVEKETIKELKEELADYKEDVEELREVRQV--VKEPVRESRAAKL 621
Hs      DACSKLQE--QKKSLTKEKEELELLKEDVQDYSEDLQEIKKELSKTGEEKYVEESKASKR 607
Mm      DACSKLQE--QKKSLTKEKEELELLKEDVQDYSEDLQEIKKELSKTGEEKYIEESAASKR 606
             :            .. :  *:*.: .      :                :  :

Fh      LASRVGRMIGEMDTMMDKLAEEKQQLLKNIELHEVHVKHSTEPTEKSEILDAIKADHERV 707
Sm      LESRVENLIKEMDTMVDKLNDKRTQLLKDIEVCESHINKSTESKERSKIMDQIKADHDRM 479
Ce      LRHKLNSMIEDVDSMVDKLEDEKRNIREMLI--------------DPTVENSVDLKKERE 603
Dm      LYNRVNKMISQLDNVLNDLEARQHQIKQAES--------------SDYAASSPTVEPQQM 667
Hs      LTKRVQQMIGQIDGLISQLEMDQQAGKLAPA--------------NGMP------TGENV 647
Mm      LSKRVQQMIGQIDGLITQLETTQQDGKLGPS--------------QSTP------TGESV 646
        *  ::  :* ::* :: .*   :                                  :

Fh      VDINDLLLALRRLQKVP-----DDTR---WQKILDVLDEDHDGKIEMQHVLSVIELLGAE 759
Sm      VDINDLLVSLKRIRNIP-----DDTR---WEKILKVLDEDRDGKIELNHVLSVIELLGSE 531
Ce      VRIQDVIDSLAKLKESKGEQADDGEQRERIEALLKTIDEDSDGIVDKQLVLEVIELLEKH 663
Dm      VHIDELVATIRRMKEAS-----DEERFKVVGDLLVKLDADKDGVISVNEITKAVQSIDRE 722
Hs      ISVAELINAMKQVKHIP-----ESK----LTSLAAALDENKDGKVNIDDLVKVIELVDKE 698
Mm      ISITELISAMKQIKHIP-----EHK----LISLTSALDDNKDGNINIDDLVKVIDLVNKE 697
        : : ::: :: :::.       :         :   :* : ** :. : : ..:: :  .

Fh      -NVKLSSKEIKRVLEMVDNE----HLAEIVQSQEIEDEARKT-AAVDNTTTE-------- 805
Sm      -NVKLSSKEIARVLEMIDNE----HLAEEIPTEDLKADKNRLKSSVDHS----------- 575
Ce      TDVHVSAAQMASMIGTLKK------EDEVAGLTE---EIRKVQSGNYQMPILPTGPSSAD 714
Dm      -ATNIDKKQLEEFTELLSKLASRRRHEEIVHIDDLMNNIKVLKETSDEAR-LKHIEAVLE 780
Hs      -DVHISTSQVAEI----------------------------------------------- 710
Mm      -DVQISTTQVAEI----------------------------------------------- 709
          .::.  ::  .

Fh      -----------------------------------------------------QVQS----- 809
Sm      -----------------------------------------------------QVTS----- 579
Ce      AFNQ-PQGVYTASK---------------------QVPARSEQEGVEQKQQVTSTL--- 748
Dm      KFDADKDGVVTVNDIRKVLESIGRDNIKLSDKAIEELISLLDKEQVLQAEQKIEKAIAKS 840
Hs      ------------------------------------VATLEKEEKVEEKEKAKEKA--- 730
```

```
Mm      -------------------------------------VATLEKEEKIEEKEKAKEKA--- 729
                                                    :  .

Fh      ------------------------------------------------------------ 809
Sm      ------------------------------------------------------------ 579
Ce      ------------------------------------------------------------ 748
Dm      MKEAEKLKSEVDKADKDLSKLVNDIHDSAKEIQDIANEMRDKEETVPDKAKELKAEPAFK 900
Hs      EKEVAEVKS--------------------------------------------------- 739
Mm      EKEAAEVKN--------------------------------------------------- 738

Fh      ------------------ESLSSPGSSTVITDATTTGGISASSGAPKSSDSTISNQTDRK 851
Sm      ------------------K----------------------------------------- 580
Ce      ------QQNSSK---------TSSSSTTKSTSD--SKSA--------------------- 770
Dm      DTAKTLKDNAKDLDDLAKDPKSDPKSPTKASTG--SGPAGLSGGGPSSGSSGIATGSTTE 958
Hs      ------------------------------------------------------------ 739
Mm      ------------------------------------------------------------ 738

Fh      R------------------------------------------------------      852
Sm      -------------------------------------------------------      580
Ce      -------------------------------------------------------      770
Dm      SALREAAERQMEKILPSTDIGLPPTIQTPSQPPTSKKATATASTLSTTITAKKLL      1013
Hs      -------------------------------------------------------      739
Mm      -------------------------------------------------------      738
```

Glyceraldehyde-3-phosphate dehydrogenase (Gapdh):

```
                                  Gp_dh_N
Fh      MGKARVGINGFGRIGRLVLRAAVEKGIVDVVAVNDPFIDLDYMRYMLKYDSTHGRFPYDI 60
Sm      MSRAKVGINGFGRIGRLVLRAAFLKNTVDVVSVNDPFIDLEYMVYMIKRDSTHGTFPGEV 60
Ce      MTKPSVGINGFGRIGRLVLRAAVEKDSVNVVAVNDPFISIDYMVYLFQYDSTHGRFKGTV 60
Dm      --MSKIGINGFGRIGRLVLRAAIDKGA-NVVAVNDPFIDVNYMVYLFKFDSTHGRFKGTV 57
Hs      MGKVKVGVNGFGRIGRLVTRAAFNSGKVDIVAINDPFIDLNYMVYMFQYDSTHGKFHGTV 60
Mm      --MVKVGVNGFGRIGRLVTRAAICSGKVEIVAINDPFIDLNYMVYMFQYDSTHGKFNGTV 58
           :*:********** ***. ..  ::*::*****.::** *::: ***** *    :
                                Gp_dh_N
Fh      TIDQNKLIVRN-----NPISVHNERDPTQIPWGASQAEYVVESTGVFTTIDKAKAHLAG- 114
Sm      STENGKLKVNG-----KLISVHCERDPANIPWDKDGAEYVVESTGVFTTIDKAQAHIKNN 115
Ce      AHEGDYLLVAKEGKSQHKIKVYNSRDPAEIQWGASGADYVVESTGVFTTIEKANAHLKG- 119
Dm      AAEGGFLVVNG-----QKITVFSERDPANINWASAGAEYIVESTGVFTTIDKASTHLKG- 111
Hs      KAENGKLVING-----NPITIFQERDPSKIKWGDAGAEYVVESTGVFTTMEKAGAHLQG- 114
Mm      KAENGKLVING-----KPITIFQERDPTNIKWGEAGAEYVVESTGVFTTMEKAGAHLKG- 112
          :  . * :        : *.:. .***::* *     *:*:*********::**  :*: .
                       Gp_dh_N                             Gp_dh_C
Fh      GAKKVIISAPSADAPMFVFGVNHEKYSKD-MSVVSNASCTTNCLAPLAKVINDKFGIVEG 173
Sm      RAKKVIISAPSADAPMFVVGVNENSYEKS-MSVVSNASCTTNCLAPLAKVIHDKFEIVEG 174
Ce      GAKKVIISAPSADAPMFVVGVNHEKYDHANDHIISNASCTTNCLAPLAKVINDNFGIIEG 179
Dm      GAKKVIISAPSADAPMFVCGVNLDAYKPD-MKVVSNASCTTNCLAPLAKVINDNFEIVEG 170
Hs      GAKRVIISAPSADAPMFVMGVNHEKYDNS-LKIISNASCTTNCLAPLAKVIHDNFGIVEG 173
Mm      GAKRVIISAPSADAPMFVMGVNHEKYDNS-LKIVSNASCTTNCLAPLAKVIHDNFGIVEG 171
         **:************** ***  :  *.      ::*****************:*:*  *:**
                                Gp_dh_C
Fh      LMTTVHSYTATQKVVDGPSSKAWRDGRGAGQNIIPASTGAAKAVGKVIPELNGKLTGMAF 233
Sm      LMTTVHSFTATQKVVDGPSSKLWRDGRGAMQNIIPASTGAAKAVGKVIPALNGKLTGMAF 234
Ce      LMTTVHAVTATQKTVDGPSGKLWRDGRGAGQNIIPASTGAAKAVGKVIPELNGKLTGMAF 239
Dm      LMTTVHATTATQKTVDGPSGKLWRDGRGAAQNIIPASTGAAKAVGKVIPALNGKLTGMAF 230
Hs      LMTTVHAITATQKTVDGPSGKLWRDGRGALQNIIPASTGAAKAVGKVIPELNGKLTGMAF 233
Mm      LMTTVHAITATQKTVDGPSGKLWRDGRGAAQNIIPASTGAAKAVGKVIPELNGKLTGMAF 231
        ******: *****.*****.* ******* ****************** **********
                                Gp_dh_C
```

```
Fh      RVPTADVSVVDLTCRLNKAAKYDEIKAAVKAAADGPMKGILDYTEELLVSSDMLGTHCSS 293
Sm      RVPTPDVSVVDLTCRLGKGASYEEIKAAVKAAASGPLKGILEYTEDEVVSSDFVGSTSSS 294
Ce      RVPTPDVSVVDLTARLEKPASLDDIKKVIKAAADGPMKGILAYTEDQVVSTDFVSDTNSS 299
Dm      RVPTPNVSVVDLTVRLGKGASYDEIKAKVQEAANGPLKGILGYTDEEVVSTDFLSDTHSS 290
Hs      RVPTANVSVVDLTCRLEKPAKYDDIKKVVKQASEGPLKGILGYTEHQVVSSDFNSDTHSS 293
Mm      RVPTPNVSVVDLTCRLEKPAKYDDIKKVVKQASEGPLKGILGYTEDQVVSCDFNSNSHSS 291
        **** :******* ** * *. ::**  :: *:.**:**** **:. :** *: .   **

                 Gp_dh_C
Fh      VFDAQAGISLNDNFVKLISWYDNEFGYSCRVVDLINHMFRVDHS  337
Sm      IFDAKAGISLNNNFVKLVSWYDNEFGYSCRVVDLITHMHKVDHA  338
Ce      IFDAGASISLNPHFVKLVSWYDNEFGYSNRVVDLISYIATKA--  341
Dm      VFDAKAGISLNDKFVKLISWYDNEFGYSNRVIDLIKYMQSKD--  332
Hs      TFDAGAGIALNDHFVKLISWYDNEFGYSNRVVDLMAHMASKE--  335
Mm      TFDAGAGIALNDNFVKLISWYDNEYGYSNRVVDLMAYMASKE--  333
         *** *.*:** :****:******:*** **:**: ::
```

Protein phosphatase 1 catalytic subunit beta (Ppp1cb):

```
                                                  PP2Ac
Sm      --MTSEVDVDELISRLLEVRSSRPGTTVNMKEEEVRYLCVTSRQIFLSQPILLELQAPLK 58
Hs      -MADGELNVDSLITRLLEVRGCRPGKIVQMTEAEVRGLCIKSREIFLSQPILLELEAPLK 59
Ce      -MDVEKLNLDNIISRLLEVRGSKPGKNVQLTESEIKGLCQKSREIFLSQPILLELEAPLK 59
Dm      --MSDIMNIDSIISRLLEVRGARPGKNVQLSESEIRSLCLKSREIFLSQPILLELEAPLK 58
Fh      MSDIDKLNVDSVIARLLEVRGSRPGKNVQLTEAEIRALALKSREIFLRQPILLELEAPLK 60
Mm      MADIDKLNIDSIIQRLLEVRGSKPGKNVQLQENEIRGLCLKSREIFLSQPILLELEAPLK 60
              :::*.:* ******..:**. *:: * *:: *. .**:*** *******:****

                                 PP2Ac
Sm      ICGDIHGQYTDLLRLFEYGGFPPESNYLFLGDYVDRGKQSLETICLLLAYKIKHPENFFL 118
Hs      ICGDIHGQYTDLLRLFEYGGFPPEANYLFLGDYVDRGKQSLETICLLLAYKIKYPENFFL 119
Ce      ICGDVHGQYYDLLRLFEYGGFPPESNYLFLGDYVDRGKQSLETICLLLAYKIKYPENFFL 119
Dm      ICGDIHGQYYDLLRLFEYGGFPPESNYLFLGDYVDRGKQSLETICLLLAYKIKYAENFFL 118
Fh      ICGDIHGQYYDLLRLFEYGSFPPEANYLFLGDYVDRGKQSLETICLLLAYKIKYPENFFL 120
Mm      ICGDIHGQYYDLLRLFEYGGFPPESNYLFLGDYVDRGKQSLETICLLLAYKIKYPENFFL 120
        ****:**** *********.****:****************************: *****

                                 PP2Ac
Sm      LRGNHECAAINRIYGFYDECKRRFSVRLWKTFTDCFNCLPIAAIVDSKIFCCHGGLSPDL 178
Hs      LRGNHECASINRIYGFYDECKRRFNIKLWKTFTDCFNCLPIAAIVDEKIFCCHGGLSPDL 179
Ce      LRGNHECASINRIYGFYDECKRRYNIKLWKTFTDCFNCLPVAAIIDEKIFCCHGGLSPDL 179
Dm      LRGNHECASINRIYGFYDECKRRYTIKLWKTFTDCFNCLPVAAIVDEKIFCCHGGLSPDL 178
Fh      LRGNHECASINRIYGFYDECKRRYNIKLWKTFTDCFNCLPIVAIVDEKIFCCHGGLSPDL 180
Mm      LRGNHECASINRIYGFYDECKRRYNIKLWKTFTDCFNCLPIAAIVDEKIFCCHGGLSPDL 180
        ********:**************:.::*************:.**:*.*************

                                 PP2Ac
Sm      QNMDQIRRIMRPSDIPDTGLLCDILWSDPDKDVNGWAENDRGVSFTFGPDVVTKFLNRHD 238
Hs      QSMEQIRRIMRPTDVPDTGLLCDLLWSDPDKDVQGWGENDRGVSFTFGADVVSKFLNRHD 239
Ce      QSMEQIRRIMRPTDVPDQGLLCDLLWSDPDKDVTGWGENDRGVSFTFGPEVVAKFLHKHD 239
Dm      SSMEQIRRIMRPTDVPDQGLLCDLLWSDPDKDTMGWGENDRGVSFTFGAEVVGKFLQKHE 238
Fh      HSMEQIRRIMRPTDVPEQGLLCDLLWSDPDKDITGWGENDRGVSFTFGADVVTKFLHNHD 240
Mm      QSMEQIRRIMRPTDVPDQGLLCDLLWSDPDKDVLGWGENDRGVSFTFGAEVVAKFLHKHD 240
         .*:********:*:*:*: *****:********  **.*********** :** ***:.*:

                                PP2Ac
Sm      MDLICRAHQVVEDGFEFFCRRQLVTLFSAPNYCGEFDNAGGMMSVDENLTCSFQILKPSE 298
Hs      LDLICRAHQVVEDGYEFFAKRQLVTLFSAPNYCGEFDNAGGMMSVDETLMCSFQILKPSE 299
Ce      LDLICRAHQVVEDGYEFFAKRQLVTLFSAPNYCGEFDNAGSMMTVDETLMCSFQILKPAD 299
Dm      FDLICRAHQVVEDGYEFFAKRQLVTLFSAPNYCGEFDNAGAMMSVDDTLMCSFQILKPAD 298
Fh      LDLICRAHQVVEDGYEFFAKRQLVTLFSAPNYCGEFDNAGAMMSVDESLTCSFQILKPAD 300
Mm      LDLICRAHQVVEDGYEFFAKRQLVTLFSAPNYCGEFDNAGAMMSVDETLMCSFQILKPAE 300
        :*************:***.:*********************.**:**:.* ********::

Sm      KKA-KYQYQGVNTTGNQARLPARIP---------------------         322
Hs      KK-AKYQYGGLNSG--RPVTPPRTANPPKKR---------------         327
Ce      KKKYPYGAGGVGSN--RPVTPPRNAPAAQPKKGAKK----------         333
Dm      KRRFVYPNFGS-SG--RPLTPPRGANNKNKKK--------------         327
```

```
Fh      KKKFNLGSLN--AV--WPVTPPRNTKLKDK----------------       326
Mm      KKKPN-------AT--RPVTPPRVGSGLNPSIQKASNYRNNTVLYE       337
        *:          :       * *
```

Proteasome subunit beta type 7 (Psmb7):

```
Ce      MATADVKSTVPHMDMDRGGAFDFSNCIRNQAM----CKMGGKAPKLTSTGTTIVAVAFKG 56
Fh      ------------------------------------------------------------ 0
Sm      --------MSSLVVDRRSGGFSFENCYRNKLI----EQKGFELPKAVKTGTTICGVVFKN 48
Dm      -------MDLDNARDLPRAGFNFDNCKRNATL----LNRGFKPPTTTKTGTTIVGIIYKD 49
Hs      -------MAAVSVYAPPVGGFSFDNCRRNAVLEADFAKRGYKLPKVRKTGTTIAGVVYKD 53
Mm      -------MAAVSVFQPPVGGFSFDNCRRNAVLEADFAKKGFKLPKARKTGTTIAGVVYKD 53

                                          Proteasome
Ce      GLVMGADSRATAGNIIADKHCEKVHKLTESIYACGAGTAADLDQVTKMLSGNLRLLELNT 116
Fh      -------------------------MTLSFCSCCGAGTAADTEMTTKMISSKVELHRLNT 35
Sm      GVVLGADTRATEGTVVAEKNCSKIHRISENIYCCGAGTAADTQMVTLMISSQVELHSLNT 108
Dm      GVILGADTRATEGPIVSDKNCAKIHYLAKNIYCCGAGTAADTEMTTDLISSQLELHRLQT 109
Hs      GIVLGADTRATEGMVVADKNCSKIHFISPNIYCCGAGTAADTDMTTQLISSNLELHSLST 113
Mm      GIVLGADTRATEGMVVADKNCSKIHFISPNIYCCGAGTAADTDMTTQLISSNLELHSLTT 113
                                     .******** : .* ::*.::.*  * *
                              Proteasome
Ce      GRKARVITALRQAKQHLFN-----------YQGYIGAYLLIGGVDPTGPHLYMCSANGTT 165
Fh      GRQPRVILPLRLLKDYLFGYRVSLFRVLEIFQGYVGAALILGGVDYQGPHLYSVSPHGCS 95
Sm      GRTPRVIAPLRLLKRYLYQ-----------YQGYVGAALVLGGVDSTGPHLYSIAPHGST 157
Dm      DREVRVVAANTMLKQMLFR-----------YQGHISAALVLGGVDKTGPHIYSIHPHGSS 158
Hs      GRLPRVVTANRMLKQMLFR-----------YQGYIGAALVLGGVDVTGPHLYSIYPHGST 162
Mm      GRLPRVVTANRMLKQMLFR-----------YQGYIGAALVLGGVDVTGPHLYSIYPHGST 162
        .*  **:       *  *:           :**::.* *::****  ***:*    :* :
                          Proteasome
Ce      MAFPFTAQGSGSYAAITILERDFKVDMTKDEAEKLVQRALEAGMHGDNASGNSLNLVIIE 225
Fh      DKLPYITMGSGCLAALSVLESRFRFDLSRTEAMQLVRDAIAAGILNDLGSGSNVDLCVIT 155
Sm      DKLPYITMGSGSLACMSVLESRFKFDMEQDEAVKLVRDGIAAGIFNDMGSGSNVDICIIT 217
Dm      DKLPYATMGSGSLAAMTVFESRWKPDLSEEEGKKLVRDAIASGVFNDLGSGSNIDLCVIR 218
Hs      DKLPYVTMGSGSLAAMAVFEDKFRPDMEEEEAKNLVSEAIAAGIFNDLGSGSNIDLCVIS 222
Mm      DKLPYVTMGSGSLAAMAVFEDKFRPDMEEEEAKKLVSEAIAAGIFNDLGSGSNIDLCVIS 222
          :*: : ***. *.::::*  :: *: . *. :**  .: :*: .* .**..::: :*
                              Pr_beta_C
Ce      PSETVFKGPIVPEFCKRPEPNDLVYKFQAGATKVLKHKTYK--YDVVES----------- 272
Fh      KDSTEYIRPHDVAN--KKGQRASKYNLPPGSTAVLSQKIQPVEYDVVTTRVIRDLPDPKT 213
Sm      KDGTTYIRSYDEAN--VKGKRAEKYNPPEGTTSVLHKSVHHVEFDVVTTRVVRDIPAHSV 275
Dm      KGSVEYLRNYELAN--KKGKRQLDYRFKTGTSTVLHTNIKDLLVTERVQAV--------- 267
Hs      KNKLDFLRPYTVPN--KKGTRLGRYRCEKGTTAVLTEKITPLEIEVLEETV--------- 271
Mm      KSKLDFLRPFSVPN--KKGTRLGRYRCEKGTTAVLTEKVTPLEIEVLEETV--------- 271
         .   :                  .   *.    *:: **   .

Ce      --MDITH 277
Fh      EAMDTS- 219
Sm      ETMDLS- 281
Dm      -PMEIS- 272
Hs      QTMDTS- 277
Mm      QTMDTS- 277
          *: :
```

Supplementary Figure S9: Multiple alignment of amino acid sequences of orthologs for kinase genes. Accession numbers of gene orthologs for *C. elegans* (Ce), *F. hepatica* (Fh), *S. mansoni* (Sm), *D. melanogaster* (Dm), *H. sapiens* (Hs) and *M. musculus* (Mm) are listed in Supplementary Table S1. Conserved protein domains are highlighted. * indicates identical amino acids.

ABL proto-oncogene 1, non-receptor tyrosine kinase *(*Abl1*)*:

```
Fh      ------------------------------------------------------------ 0
Sm      ------------------------------------------------------------ 0
Ce      ------------------------------------------------------------ 0
Dm      MGAQQGKDRGAHSGGGGSGAPVSCIGLSSSPVASVSPHCISSSSGVSSAPLGGGSTLRGS 60
Hs      ------------------------------------------------------------ 0
Mm      ------------------------------------------------------------ 0


Fh      --------------MG---GNSG---------------------KLATSK---------- 12
Sm      ------------------------------------------------------------ 0
Ce      -----------------------MGHSHSTGKEIN-------DNELFTCEDPVFDQPVAS 30
Dm      RIKSSSSGVASGSGSGGGGGGSGSGLSQRSGGHKDARCNPTVGLNIFTEHNEALLQS--R 118
Hs      --------------------------------------MLEICLK--------LVGC--K 12
Mm      ----------------------------------------MISFD--------LLS---- 8


Fh      -----------------------NGHLQSPRLSSN-----SQNVEGTRTGN-SSYQ--ER 41
Sm      -----------------------MSRVDTSDLSKN-----PKPADLN------KVD--DK 24
Ce      PKSEISSKLAEEIERSKSPLILEMFRPTFDTFRPPNSDSST--FR-----GSQSRE--DL 81
Dm      PLPHIPAGS------TAASLL-----ADAAELQQHQQDSGGLGLQGSSLGGGHSSTTSVF 167
Hs      SKK----GL------SSSSSC-----YLEEALQRP---------------VASDFEPQGL 42
Mm      --D----EL------HLKLLV-----LDVEALQRP---------------VASDFEPQGL 36
                                       :                     .
                                                    SH3
Fh      GSNSSYPLDINCQ--SASETAVHTDKELMVVLYDFTESMRSQISIKRGELVRLLGYSPAG 99
Sm      LSDIRSYTKMDVQ--SSSDSLVLGRGGIMVVLYDFSESMSSQISIKRGELVRLLSYSPAG 82
Ce      VACSSMNSVNNVHDMNTVSSSSSSSAPLFVALYDFHGVGEEQLSLRKGDQVRILGYNKNN 141
Dm      ESAHRWTSKEN----LLAPGPEEDDPQLFVALYDFQAGGENQLSLKKGEQVRILSYNKSG 223
Hs      SEAARWNSKEN----LLA-GPSENDPNLFVALYDFVASGDNTLSITKGEKLRVLGYNHNG 97
Mm      SEAARWNSKEN----LLA-GPSENDPNLFVALYDFVASGDNTLSITKGEKLRVLGYNHNG 91
                  :                ::*.****     . :*: :*: :*:*.*.  .
                                          SH3
Fh      DWSEVETSGLAHPATHPMMETELTNTSTQPSQTQIKSDLTTQRDSVGPGGVGGGSTMSTG 159
Sm      DWSEVEASLVLPPLPKPGPGSSTSGSESHPSQCKGGAT---VKH-------SHGPTMETG 132
Ce      EWCEARLYSTRK------------------------------------------------ 153
Dm      EWCEAHS----------------------------------------------------- 230
Hs      EWCEAQT----------------------------------------------------- 104
Mm      EWCEAQT----------------------------------------------------- 98
        :*.*..
                        SH3
Fh      SGHSINNSAFGNAPVRYRRGWVPTSYLAPANVLSDPNQRLTGHFLPHEVIFPSVGSTSSM 219
Sm      N--SV-NTSCNYLFNNYRRGWVPTSYLATANVFQSSAPSKQ--SYPQQLI-GCEGS--EK 184
Ce      -------NDASNQRRLGEIGWVPSNFIAPYNS---------------------------- 178
Dm      --------------DSGNVGWVPSNYVTPLNS---------------------------- 248
Hs      --------------KNG-QGWVPSNYITPVNS---------------------------- 121
Mm      --------------KNG-QGWVPSNYITPVNS---------------------------- 115
                           ****:.:::  *

Fh      ERCNVTGMDSKVNLVSGPSPMLVMRPGQMGSRSHSSAQATQQQFQPQTNMVDSSPLGLQG 279
Sm      PNCSVNAP-----CLSNS--QSIIANDNMQSR----VQNVSNKGPVHHVMMDSSPLSLLE 233
Ce      ------------------------------------------------------------ 178
Dm      ------------------------------------------------------------ 248
```

```
Hs      ------------------------------------------------------------ 121
Mm      ------------------------------------------------------------ 115

                                  SH2
Fh      PSLVLYPWYHGAVSRHAGEQLLRSGITGSFLVRASESAPGQLSVTVRHLGRVYHYRISQD 339
Sm      PSLLPYSWYHGAVSRQAGEHLLRSGITGSFLVRASESAPGQLSVTVRHLGRVYHYRISQD 293
Ce      --LDKYTWYHGKISRSDSEAILGSGITGSFLVRESETSIGQYTISVRHDGRVFHYRINVD 236
Dm      --LEKHSWYHGPISRNAAEYLLSSGINGSFLVRESESSPGQRSISLRYEGRVYHYRISED 306
Hs      --LEKHSWYHGPVSRNAAEYLLSSGINGSFLVRESESSPGQRSISLRYEGRVYHYRINTA 179
Mm      --LEKHSWYHGPVSRNAAEYLLSSGINGSFLVRESESSPGQRSISLRYEGRVYHYRINTA 173
          *  : **** :**   .* :* ***.****** **:: ** ::::*: ***:****.
                  SH2
Fh      VRGLFYITNVHRFPTVVQLIDHHSRS--ADGLVCPLLYPVPKLQYPTSHMTSVAYSSVPT 397
Sm      SRGLFYITEAHRFPTVVQLIEHHSRS--ADGLVCPLLYSVPKPQFLNQPMQQSCYSSVPQ 351
Ce      NTEKMFITQEVKFRTLGELVHHHSVH--ADGLICLLMYPASKKDKGRGL----------- 283
Dm      PDGKVFVTQEAKFNTLAELVHHHSVPHEGHGLITPLLYPAPKQNKP-TV----------- 354
Hs      SDGKLYVSSESRFNTLAELVHHHSTV--ADGLITTLHYPAPKRNKP-TV----------- 225
Mm      SDGKLYVSSESRFNTLAELVHHHSTV--ADGLITTLHYPAPKRNKP-TI----------- 219
            .:::.  :* *: :*:.***    ..**:   *  *  .  *  :

Fh      HAIPSHPHTHLIGGAAQTILPPVPTHPSADSST---TGPDQASFHPRPHNTAQQGMEPFL 454
Sm      TLPSSR-----IPGPNQFEVNPIVHQSNAEISNFPLTHINPVSHVPRPFHPSH--INNSE 404
Ce      ------------------------------------------------------------ 283
Dm      ------------------------------------------------------------ 354
Hs      ------------------------------------------------------------ 225
Mm      ------------------------------------------------------------ 219

                                                TyrKc
Fh      GLSRTDEPFRKQSWDGWEIDRNEILMRQKLGCGQYGDVYEAIWKRLNTVVAVKTLKQDVN 514
Sm      RLSACSDSIGSMEFDGWEIDRSEIIMRQKLGCGQYGDVYEAVWKRFNSVVAVKTLKQDVN 464
Ce      ------FSLSPNAPDEWELDRSEIIMHNKLGGGQYGDVYEGYWKRHDCTIAVKALKEDA- 336
Dm      ------FPLSPE-PDEWEICRTDIMMKHKLGGGQYGEVYEAVWKRYGNTVAVKTLKEDT- 406
Hs      ------YGVSPN-YDKWEMERTDITMKHKLGGGQYGEVYEGVWKKYSLTVAVKTLKEDT- 277
Mm      ------YGVSPN-YDKWEMERTDITMKHKLGGGQYGEVYEGVWKKYSLTVAVKTLKEDT- 271
                .     * **: *.:* *::*** ****:***. **:  .  .:***:**:*.
                                   TyrKc
Fh      LNVNDFLKEASIMKKLRHRNLVQLLGVCTREPPLYLITEYMSNGNLLNYLRTRSPGELTP 574
Sm      LNVNDFLKEAAIMKKLRNRNLVQLLGVCTREPPLYLITEYMPNGNLLNYLRTRSPGELTP 524
Ce      MPLHEFLAEAAIMKDLHHKNLVRLLGVCTHEAPFYIITEFMCNGNLLEYLRRTDKSLLPP 396
Dm      MALKDFLEEAAIMKEMKHPNLVQLIGVCTREPPFYIITEFMSHGNLLDFLRSAGRETLDA 466
Hs      MEVEEFLKEAAVMKEIKHPNLVQLLGVCTREPPFYIITEFMTYGNLLDYLRECNRQEVNA 337
Mm      MEVEEFLKEAAVMKEIKHPNLVQLLGVCTREPPFYIITEFMTYGNLLDYLRECNRQEVSA 331
        : :.:** **::**.::: ***:*:****:* *:*:***:*  ****::**   .   :
                                   TyrKc
Fh      PVLLYMAVQIASGMSYLEANNFIHRDLAARNCLVGERHLIKVADFGLARYMQRQDTYTAR 634
Sm      LTLLYMAVQIASGMAYLEANNFIHRDLAARNCLVGDQHLIKVADFGLARYMQRQDTYTAR 584
Ce      IILVQMASQIASGMSYLEARHFIHRDLAARNCLVSEHNIVKIADFGLARFM-KEDTYTAH 455
Dm      VALLYMATQIASGMSYLESRNYIHRDLAARNCLVGDNKLVKVADFGLARLM-RDDTYTAH 525
Hs      VVLLYMATQISSAMEYLEKKNFIHRDLAARNCLVGENHLVKVADFGLSRLM-TGDTYTAH 396
Mm      VVLLYMATQISSAMEYLEKKNFIHRDLAARNCLVGENHLVKVADFGLSRLM-TGDTYTAH 390
          *: ** **:*.* ***  .::************.:.:::*:*****:* *   *****:
                                   TyrKc
Fh      NGAKFPIKWTAPEGLAYYVFSSKSDVWAFGVVLWELATYGLSPYPGVELHDVYHILEKGY 694
Sm      NGAKFPIKWTAPEGLSYYLFSSKSDVWAFGVVLWELATYGLSPYPGVELHDVYHLLEKGY 644
Ce      AGAKFPIKWTAPEGLAFNTFSSKSDVWAFGVLLWEIATYGMAPYPGVELSNVYGLLENGF 515
Dm      AGAKFPIKWTAPEGLAYNKFSTKSDVWAFGVLLWEIATYGMSPYPAIDLTDVYHKLDKGY 585
Hs      AGAKFPIKWTAPESLAYNKFSIKSDVWAFGVLLWEIATYGMSPYPGIDLSQVYELLEKDY 456
Mm      AGAKFPIKWTAPESLAYNKFSIKSDVWAFGVLLWEIATYGMSPYPGIDLSQVYELLEKDY 450
         ************.*::  ** *********:***:****::***.::* :**  *::.:
                   TyrKc
Fh      RMERPHGCPEAVYSIMLRCWAWEASLRPSFTEVHAELEQMYTTMNIEAEVARELEKRHAA 754
Sm      RMERPHGCPEAVYSIMLRCWSWDPNLRPSFSEIHAELEQMYTTMNIEAEVALELGKQPAN 704
Ce      RMDGPQGCPPSVYRLMLQCWNWSPSDRPRFRDIHFNLENLISSNSLNDEVQKQLKKNNDK 575
```

```
Dm      RMERPPGCPPEVYDLMRQCWQWDATDRPTFKSIHHALEHMFQESSITEAVEKQLNANATS 645
Hs      RMERPEGCPEKVYELMRACWQWNPSDRPSFAEIHQAFETMFQESSISDEVEKELGKQGVR 516
Mm      RMERPEGCPEKVYELMRACWQWNPSDRPSFAEIHQAFETMFQESSISDEVEKELGKRGTR 510
        **: * ***  ** :*  ** *. . ** * .:*  :* :    .:   *  :*  .

Fh      FTAASSNNQPTGEPSEPVRDLGTGDL---------------------------------- 780
Sm      FIPQQQQQPLVSS--NRFTEIQPHDLQGN------SVNQFNMVFKESLPSSVIPTEIVNS 756
Ce      KLESDKRRSNVRERSD-------SKSRHSSHHD--RDRDRESLHSRN------------- 613
Dm      ASSSAPSTSGVATGGGATTTTAASGCASSSSATASLSLTPQMV-KKGLPGG--------- 695
Hs      GAVSTLLQAPE-------LPTKTRTSRRAAEHR-DTTDVPEMPHSKGQGES--------- 559
Mm      GGAGSMLQAPE-------LPTKTRTCRRAAEQK-DAPDTPELLHTKGLGES--------- 553


Fh      ---------------FVDVRCSGSMSAHGVIEPLNPNTISNSIWLR--ATDSVHHSSQAA 823
Sm      DHIISRHNNAIHSTSNIDFTCNAT-------ESSRPNPVSSYVIMNSVANHSIHHHVENN 809
Ce      --------------------------------------------------------SNPE 617
Dm      -------------------------------QALTPN-----------AHHNDPHQQQAS 713
Hs      -------------------------------DPLDHE----------------PA-VSPL 571
Mm      -------------------------------DALDSE----------------PA-VSPL 565
                                                                 .

Fh      HP---SGL------------------------PNRDVHEGPVRPDVDGG-----DEIQAD 851
Sm      IASLNNNIHNCMKKEKTIFIDQN--------RLSKDKHSVLPYPSSSGGGVGGSDRFDLI 861
Ce      IPNRSFIRTDDSVS---FFNPST---TSKVTSFRAQGPPFPPPPQQNTKP----KLLKSV 667
Dm      TPMSETGSTSTKLS---TFSSQGKGNVQMRRTTNKQGKQAPAPPKRTS-------LLSSS 763
Hs      LPRKERGPPEGGLNEDERLLPKDKKTNLFSALIKKKKKTAPTPPKRSS-------SFREM 624
Mm      LPRKERGPPDGSLNEDERLLPRDRKTNLFSALIKKKKKMAPTPPKRSS-------SFREM 618
                                           .       *.           :

Fh      -D-----GDEDDEEGEF-DESDEEDAQQQWH-EHSNAIQQ------PSDHMARSPEPASS 897
Sm      -TDNLAAVSLDNNNDDI-DNSDDDDENKRPASSNNHAVQYESNSETNPDHMLL---PTSS 916
Ce      LNSNARHASEEFERNE-QDDVVPLAEKN-VR-KAVTRLGG---------T---MP----K 708
Dm      RDSTYRE---EDPANARCNFIDDLSTNG-LA-RDINSLTQRYDSETDPAA---DPDTDAT 815
Hs      DGQPERRGAGEEEGRDISNGALAF--------------TP--LDTADPAK---SPKPSN- 664
Mm      DGQPDRRGASEDDSRELCNGPPAL--------------T---SDAAEPTK---SPKASN- 657
                  :       :

Fh      SNVVGALSRYHVSDPTVVPNALEGVSPAHIMDRLCGQIRAMSLAPR---DGSHLCRTSKS 954
Sm      ---LGQ--------IKFLPNSV--------ISSCSPQMTSIHESSQNGFSNLIQCRTPFS 957
Ce      GQRIDAYL--------------------------------------DSMRRVD------- 723
Dm      GDSLEQSLSQV--IAAPVTNKMQH---------------SLHSGGGGGGIGPR------- 851
Hs      GAGV--------------PN---G---------------ALRESGGSGFRSPH------- 685
Mm      GAGV--------------PN---G---------------AFREPGNSGFRSPH------- 678
           :

Fh      GRWDDNTS-QVAMFASQSPVPITQGQRYYTPPPNVGGLPSAQPHRDLVI----------- 1002
Sm      SNYLTQTQYSPASLSDNGFLPFGEKLCYFIPMSN---NPSYQPNSHITSSNITQN---CG 1011
Ce      ---------------------------------------SWKESTDA--------DNEGA 736
Dm      ---------------------------------------SSQQH---------------- 856
Hs      ---------------------------------------LWKKSSTLTSSRLATGEEEGG 706
Mm      ---------------------------------------MWKKSSTLTGSRLAAAEEESG 699
                                                 :

Fh      ----------------GDVPNSSASNSKHRPMAATSSQSMNSAGYARE-KSDRSTSARPG 1045
Sm      ISNLSQFSNNNPASIVSKIPNLNVANST---------KVVNDRSAIRTNRSIHRNVTSGC 1062
Ce      GS--SSLSRTVSNDSLD---------------TLPLPDSMNSSTYVKMH------PASGE 773
Dm      ----SSFKRPTGTPVMG-----NRGLETRQSKRSQLHSQAPGPGPPSTQ------PHHGN 901
Hs      GSSSKRFLRSCSASCVP---HGAKDTEWR---SVTLPRDLQSTGRQFDS------STFGG 754
Mm      MSSSKRFLRSCSASCMP---HGARDTEWR---SVTLPRDLPSAGKQFDS------STFGG 747
                                                 .

Fh      RSRVCRTRDGTTRSRDTDTPDESGVGESIISNDSPADS---GGGVIGSA-PVNIMSTDRV 1101
Sm      NTQTSSQQNGTTRSQDTDTPDESGVGESIISNESPAESRACGGGIMSNAAGVGVIATMNS 1122
```

```
Ce      NVFLRQIRS--KLKKRSETPELDHIDSDTADETTKSEKSPF--GSLNKSSIKYPIK---- 825
Dm      NGVVTSAHP---I-T---------VGALDVM-NVKQVVNRY--GTLPKGARIGAYL---- 941
Hs      HKSEKPALP---R-KRAGENRSDQVTRGTVT-PPPRLVKKN------EEAADEVFK---- 799
Mm      HKSEKPALP---R-KRTSESRSEQVAKSTAM-PPPRLVKKN------EEAAEEGFK---- 792
        .                       :                      .


Fh      LSLSASCKTA---DTPSVLNTF--AQAKTSHPANL-----VSQSSVP--AGLPSHEVFQP 1149
Sm      ILSSAPSQVDWHFNVPFEVSMPIDDHSQHFHHAQI-----ITPTSNS--V-IHHPHSYPY 1174
Ce      -------------NAPE----FSENHSR-VSP---------------------------- 839
Dm      ----------------D----SLEDSSE-AAPA-LP----ATAPSLPPANGHA------- 968
Hs      ----------------D----IMESSPG-SSPPNLTPKPLRRQVTVAPASGLPHKEE--- 835
Mm      ----------------D----T-ESSPG-SSPPSLTPKLLRRQVTASPSSGLSHKEE--- 827

Fh      LCTAVGTAASSMSTV-LTQGTTRTVSQP-ALRLDAEAMDQFATLPPQDRIGRYLESLN-- 1205
Sm      LFQTTNTSTTRITTTTINTTTTHISSASSTQFNVNPLMEQFSTIPPHDRIGSYLKSLG-- 1232
Ce      ----------------------------------------VPVPPSRNAS------VSV 852
Dm      --------------------T------P----PAARLNPKASPIPPQQMIRSNSSGGVTM 998
Hs      ----------------AGKGS------A------LGTPAAAEPVTPTSKAGSGAPGGTSK 867
Mm      ----------------ATKGS------A----SGMGTPATAEPAPPSNKVG------LSK 855
                                                     *

Fh      ------E-------IGSE-LPSTTSTTTSAQAQTRFYLNHGAVEQQQ---LLLQQRQQQP 1248
Sm      ------E-------LDTG-CGRHHRCNE----PPTFTYHQSQPNELHLPTDILDVN-DKT 1273
Ce      RPDSKAEDSSDETTKDVGMWGPKHAVTRKIEIVKND--------S------YPNV--EGE 896
Dm      QNNAAASLNK--------L--QRHRTTTEGTMMTFSSFRAGGSSSSP-KRSASGV--ASG 1045
Hs      GPA---EESR--------V--RRHKH----------------SSESP-GRDK-------G 890
Mm      ASS---EEMR--------V--RRHKH----------------SSESP-GRDK-------G 878
              .                                     .

Fh      LSTNSLFFLQYPPPPPPPL---PVHTVPISLSPPYVPQDNEA----LFTA-N--PMTASY 1298
Sm      -FNHPSNVLHYPPPPPPVPP-PPHSSIMECCNPPSLPPTSTTNNASIQLSSD--VTSTGE 1329
Ce      -LKAKIRNLRHVPKE--------ESNT------------------SSQED----LPL--- 922
Dm      -VQPALANLEFPPPPLDLPPPPEEFE----GGPPPPPPAPESAVQAIQQHLHAQLPNNGN 1100
Hs      ----KLSRLKPA------PPPPPAASAGKAGGKPSQSPSQEAAGEAV------------- 927
Mm      ----RLAKLKPA------PPPPPACTG--KAGKPAQSPSQEAGEAGG------------- 913
                *.

Fh      LSE----------TQSASNL----RP-QNHE-----------------------LTARLE 1320
Sm      ISK----------SNISQPISPVLRS-KQYF-----------------------HSKAIT 1355
Ce      ---DATDNTN-----DSIIVIPRDEKAKVRQLVTQKVSPLQHHRPFSLQCPNNSTSSAIS 974
Dm      ISNGNGTNNNDSSHNDVSNIAPSVEEASSRFG-----VSLRKREPSTDSCSSL------- 1148
Hs      ----LGAKTKATSLVDAVNSDA-----------------------------AKP------- 948
Mm      ----PTKTKCTSLAMDAVNTDP-----------------------------TKA------- 934


Fh      DSSH------G-------YL---------------------------------------- 1327
Sm      DSET------G---TNMDQP---------------------------------------- 1366
Ce      HSEHADSSETSSLSGVYEERMKPELPRKRSN------------------GDTKVVPVTWI 1016
Dm      ----------GSPPEDLKEKLITEI-------KAAGKDTAPASHLANGSGIAVVDPVSLL 1191
Hs      ----------SQPGEGLKKPVLPATPKPQS-AKPSGTPISPAPVP--------------- 982
Mm      ----------GPPGEGLRKPVPPSVPKPQSTAKPPGTPTSPVSTP--------------- 969
                  .

Fh      ------------------------------------------------------TRCN---- 1331
Sm      ------------------------------------------------------TNCFSSNN 1374
Ce      INGE-------------------KEPNGMAR---------------------------T 1029
Dm      VTELAESMNLPKPPPQQQQKLTNGNSTGSGFKAQLKKVEPKKMSAPMPKAEPANTIIDFK 1251
```

```
Hs      -------STLPSASS----ALAGDQPSSTAF----------------------IPLISTR 1009
Mm      -------STAPAPSP----LAGDQQPSSAAF----------------------IPLISTR 996

Fh      ------------NGINVGNTG------TGNCSSAKE-----SGSKYFRCSSNQPRQRRPL 1368
Sm      QNLSQLKASSEQNNIAVDESSLNHNNSNSNNSDIKDLH---SNTSYS--KISQPRRRIKV 1429
Ce      KSLRDITSKFEQLGTASTIESKI---EEAVPYREHAL-EKKGTSKRFSMLEGSNELKHVV 1085
Dm      AHLRRVDKEKEPATPAPAP-ATVAVANNANCNTTGTLNRKEDGSKKFSQAMQKTEIKIDV 1310
Hs      VSLRKTRQPPERIASGAITKGVVLDSTEALCLAIS---RNSE------------------ 1048
Mm      VSLRKTRQPPERIASGTITKGVVLDSTEALCLAIS---RNSE------------------ 1035
                            .       .

Fh      KN---------GQDSDGEGRRRESL-ERSGEESNTEEVTRSGE---EETLSLASESPSPN 1415
Sm      DTERAVSGNAGHLDYPGVGRRHLPLDDRSEEESNTEEVPRSLE---DETLSIASDSPNNS 1486
Ce      -----------------------------------------------------PPRKNRN- 1092
Dm      T-NSNVEADAGAAGEGDLGKRRSTDD---EEQSHTEGLGSGGQGSADMTQSLYEQKPQIQ 1366
Hs      -----------------------------QMASHSAVLEA-G------------------ 1060
Mm      -----------------------------QMASHSAVLEA-G------------------ 1047

Fh      GDDPVSTNRAIELNGTDLASEEHDTEADDVYQAPEACYSMIAASCGAGNTSAASIVHDGD 1475
Sm      QDNSMSIKPDADLSG--------GGEGDDVYQAPDSYYLPNINNSNSQNLQPN------S 1532
Ce      -------------------------------------------------QDESGSIDEEP 1103
Dm      QKPAVP-----------------HKPTKLTIYATPIAKL-----------TEPASSASSTQ 1399
Hs      ------------------------------------------------------------ 1060
Mm      ------------------------------------------------------------ 1047

Fh      SLPKSLPSLITRLERLIDELVRLTQSV-SDNTVCDNNPELALLQVTDTLDAFRAEVEAYV 1534
Sm      TSQESITNL---LERTIE-LVNWTKDLCSYCEMEYEIPNASFIELSNCLDSFRIELEAYI 1588
Ce      VSKDMIVSLLKVIQKEFVNLFNLASSEI------TDEKLQQFVIMADNVQKLHSTCSVYA 1157
Dm      ISRESILELVGLLEGSLK-------HPV------NAIAGSQWLQLSDKLNILHNSCVIFA 1446
Hs      -------------------------------------------------KNLYTFCVSYV 1071
Mm      -------------------------------------------------KNLYTFCVSYV 1058
                                                         . :      :

Fh      VCRETNLDGLPTTVLHACDMLGRQTSLLAFEIGSADTSMTVDTGDSTTQRSRNPWSSLCT 1594
Sm      VEHISINPDLTKTILILCDNLGKQTTNLSLHLKTMEV-------------DKPAFSVHCN 1635
Ce      EQ---IS---PHSKFRFKELLS------QLEIYNRQIKFSHNP------RAKPVDDKLKM 1199
Dm      ENGA-MP---PHSKFQFRELVT------RVEAQSQHLRS-------AGSKNVQDNERLVA 1489
Hs      DS-I-QQ---MRNKFAFREAIN------KLENNLRELQICPAT-AGSGPAATQDFSKLLS 1119
Mm      DS-I-QQ---MRNKFAFREAIN------KLESNLRELQICPAT-ASSGPAATQDFSKLLS 1106
                    . :   : :        ..    .                   .

Fh      SILPALEDLLKNLVRLRGVESISSS---SEVSSNEKP----ESSNSNERPTAIAGPASDR 1647
Sm      SCHSCLKEIHEHIVQLLNKSKVTNAVSTITLTTGSTTSVTNQTHLHNDRSFSTTD-NTTT 1694
Ce      AFQDCFDQIMRLVDR--------------------------------------------- 1214
Dm      EVGQSLRQISNALNR--------------------------------------------- 1504
Hs      ----SVKEISDIVQR--------------------------------------------- 1130
Mm      ----SVKEISDIVRR--------------------------------------------- 1117
            ..::    : :

Fh      RVCGSVVLGKV    1658
Sm      TTTTTHVFGKV    1705
Ce      -----------    1214
Dm      -----------    1504
Hs      -----------    1130
Mm      -----------    1117
```

ABL proto-oncogene 2, non-receptor tyrosine kinase (Abl2):

```
Fh        MPTKMVGIKHTLDSSINTIGKLKKEVTNQMTKRPGYCHVKHKTWFRKVCSRHLLSEGQRG 60
Dm        ------------------------------------------------------------ 0
Ce        ------------------------------------------------------------ 0
Sm        ------------------------------------------------------------ 0
Hs        ------------------------------------------------------------ 0
Mm        ------------------------------------------------------------ 0


Fh        SADPDPLGKTNYNFLVLECNQGDPTPTVLDQSSRAGSDRRPQIPYKMTMSDFVSGSINES 120
Dm        ------------------------------------------------------------ 0
Ce        ------------------------------------------------------------ 0
Sm        ------------------------------------------------------------ 0
Hs        ---------------------------MGQQVGRVGEAPGLQQPQPRGI----RG---SS 26
Mm        ---------------------------MGQQVGRVGEAPGLQQPQPRGI----RG---SS 26


Fh        NLPSNGRLRSATH-NACPIKGSIKRSPESGTEINSTNPGNNNASNQSQAAFIPPGA--QI 177
Dm        ----------------------------MGNCLTTQKGEPDKPA-----DRIKLDDPPTI 27
Ce        ----------------------------MGSCIGKEDPPPGATS--------PVHTSSTL 24
Sm        -------------------------------------MGAQHTK---E----RPR----R 12
Hs        AARPSGRRRDPAGRTTETGFNIFTQHE-------------------------ALH----R 57
Mm        AARPSGRRRDPAGRTADAGFNVFTQHDHFASCVEDGFEGDKTGGSSPE----VLH----R 78

                                                                  SH3
Fh        LVDCGLA----TALPTSASFGHMSEVNLGTTSTGTTVTSTITATTSSANSSGSENLYVAL 233
Dm        GVGVGVP---QIPMPSHAGQ---PPEQI-----RPVPQIP-----ESETAGANAKIFVAL 71
Ce        ----G-----RESLPSHPRI---PS--I-----GPIAASSSGNTIDKNQNISQSANFVAL 65
Sm        TYHPKVDHELQINTSTSWIQNHNPPTNF----KGS-------VAHTSQINQPSDQLLIAL 61
Hs        PYGCDVE-PQALNEAIRWSSKEN----L----L--------------GATESDPNLFVAL 94
Mm        PFGCDAE-SQALNEAIRWSSKEN----L----L--------------GATESDPNLFVAL 115
                                     :                        .    :**

                                SH3
Fh        HDYVKR----VDDDLNMTKGQTFRILDRS-HCDWWYAECTTTGQRGYVPKNHLARV---- 284
Dm        YDYDAR----TDEDLSFRKGEHLEILNDT-QGDWWLARSKKTRSEGYIPSNYVAKL---- 122
Ce        FQYDAR----TDDDLSFKKDDILEILNDT-QGDWWFARHKATGRTGYIPSNYVARE---- 116
Sm        FDFTPPNTLENQSQVRVEKGDRLQLFGYSADGDWSDVECIRTNERGWIPTNYTGRIPVPP 121
Hs        YDFVAS----GDNTLSITKGEKLRVLGYNQNGEWSEVRS--KNGQGWVPSNYITPV---- 144
Mm        YDFVAS----GDNTLSITKGEKLRVLGYNQNGEWSEVRS--KNGQGWVPSNYITPV---- 165
          .::        :. : . *.: :.::. . . :*  ..   .   *::*.*:

                                                                SH2
Fh        ---------------------------------------TSLESNEWYFGELRRIEAEHYL 306
Dm        ---------------------------------------KSIEAEPWYFRKIKRIEAEKKL 144
Ce        ---------------------------------------KSIESQPWYFGKMRRIDAEKCL 138
Sm        NDHSTTLNTGSASGSSLTFQHTSSHDVSCNGSQGSLARVGLESEKWYHGAIQRSYAEYL- 180
Hs        ---------------------------------------NSLEKHSWYHGPVSRSAAEYL- 165
Mm        ---------------------------------------NSLEKHSWYHGPVSRSAAEYL- 186
                                                 .:* . **.  : *  **

                                        SH2
Fh        QLPGNDHGSFLVRVSESQSSEYSLSVREEDTIKHYRIRSRSSRTNPDLKRFYISRQLPFV 366
Dm        LLPENEHGAFLIRDSESRHNDYSLSVRDGDTVKHYRIRQL------DEGGFFIARRTTFR 198
Ce        LHTLNEHGAFLVRDSESRQHDLSLSVRENDSVKHYRIRQL------DHGGYFIARRRPFA 192
Sm        -LNSGITGSFLIRESESHPGQLTISLRYEGQIWHYRIHRD------DSNMYYVIESNKFT 233
Hs        -LSSLINGSFLVRESESSPGQLSISLRYEGRVYHYRINTT------ADGKVYVTAESRFS 218
Mm        -LSSLINGSFLVRESESSPGQLSISLRYEGRVYHYRINTT------TDSKVYVTAESRFS 239
                 *:**:* ***   : ::*:*  . : ****.             ::     *

               SH2                                              TyrKc
Fh        TIQQLVGHYTENQSGLCCRLSRPCIKPTHPEPVGLSHKLIDKWEISKSSIVLKERIGQGQ 426
Dm        TLQELVEHYSKDSDGLCVNLCKPCVQIEKPVTEGLSHRTRDQWEIDRTSLKFVRKLGSGQ 258
Ce        TLHDLIAHYQREADGLCVNLGAPCAKSEAPQTTTF--TYDDQWEVDRRSVRLIRQIGAGQ 250
Sm        SVSDLVHHHEKHSDGLACTLLYPAPKRDRTSSELRMDPSFDIREIDRTEIVMKHKLGSGQ 293
Hs        TLAELVHHHSTVADGLVTTLHYPAPKCNKPT-VYGVSPIHDKWEMERTDITMKHKLGGGQ 277
```

```
Mm          TLAELVHHHSTVADGLVTTLHYPAPKCNKPT-VYGVSPIHDKWEMERTDITMKHKLGGGQ 298
            :: :*: *:    .**    *   *. :               *   *:.: .: : .::* **
                                           TyrKc
Fh          FGEVYKAVWNK-TAIVAVKTLKPSSCDAADFLREAQIMKRLHHPNLIQLYAVCTQSEPFY 485
Dm          FGDVWEGLWNN-TTPVAIKTLKSGTMDPKDFLAEAQIMKKLRHTKLIQLYAVCTVEEPIY 317
Ce          FGEVWEGRWNV-NVPVAVKKLKAGTADPTDFLAEAQIMKKLRHPKLLSLYAVCTRDEPIL 309
Sm          YGVVYEAVWKPYNVLVAVKTLKENVTVRDEFLEEARLMKSLRHPNLVTLLGACTREPPYY 353
Hs          YGEVYVGVWKKYSLTVAVKTLKEDTMEVEEFLKEAAVMKEIKHPNLVQLLGVCTLEPPFY 337
Mm          YGEVYVGVWKKYSLTVAVKTLKEDTMEVEEFLKEAAVMKEIKHPNLVQLLGVCTLEPPFY 358
            :* *: . *:   .  **:*.** .     :** ** :** ::* :*: * ..** . *
                                           TyrKc
Fh          LVTEYMSKGSLLAYLQSPEGRG--LSIQCLVMMAAKIASGMAYLESRRHIHRDLAARNVL 543
Dm          IITELMKHGSLLEYLQAIAGKGRSLKMQTLIDMAAQIAAGMAYLESQNYIHRDLAARNVL 377
Ce          IVTELMQENLL----TFLQRRGRQCQMPQLVEISAQVAAGMAYLEEMNFIHRDLAARNIL 365
Sm          IVTEFMCNGNLLDYLRTHPRTE--LTPSVLLHMATQVARGMAYLEQHNFIHRDLAARNCL 411
Hs          IVTEYMPYGNLLDYLRECNREE--VTAVVLLYMATQISSAMEYLEKKNFIHRDLAARNCL 395
Mm          IVTEYMPYGNLLDYLRECSREE--VTAVVLLYMATQISSAMEYLEKKNFIHRDLAARNCL 416
            ::** *   . *                  *: :::::: .* ***. ..********* *
                                          TyrKc
Fh          VGEQHVVKIADFGLARM-IHNREYVAHAGARFPIKWTAPEAANYSRFTIKSDVWSFGILL 602
Dm          VGDGNIVKIADFGLARL-IKEDEYEARVGARFPIKWTAPEAANYSKFSIKSDVWSFGILL 436
Ce          INNSLSVKIADFGLARILMKENEYEARTGARFPIKWTAPEAANYNRFTTKSDVWSFGILL 425
Sm          VGTQHTIKVADFGLARCIERDLTYRAHEGAKFPIKWTAPEGLVYNLFSTKSDVWAFGILL 471
Hs          VGENHVVKVADFGLSRLMTG-DTYTAHAGAKFPIKWTAPESLAYNTFSIKSDVWAFGVLL 454
Mm          VGENHVVKVADFGLSRLMTG-DTYTAHAGAKFPIKWTAPESLAYNTFSIKSDVWAFGVLL 475
            :.     :*:*****:*       * *: **:*********.   *. *: *****:**:**
                                           TyrKc
Fh          TEIVTYGRSPYPGMHNAEVLRQVEAGYRMSKPSGCPPELYDLMLECWAADENRRPSFAEI 662
Dm          TELVTYGRIPYPGMTNAEVLTQVEHGYRMPQPPNCEPRLYEIMLECWHKDPMRRPTFETL 496
Ce          TEIVTFGRLPYPGMTNAEVLQQVDAGYRMPCPAGCPVTLYDIMQQCWRSDPDKRPTFETL 485
Sm          WEIATYGKTPYPGVELQDVYVLLERGTRMLCPEGCPEPVYELMLQCWQWLPEQRPPFSDI 531
Hs          WEIATYGMSPYPGIDLSQVYDLLEKGYRMEQPEGCPPKVYELMRACWKWSPADRPSFAET 514
Mm          WEIATYGMSPYPGIDLSQVYDLLEKGYRMEQPEGCPPKVYELMRACWKWSPADRPSFAET 535
             *:.*:*   ****:    :*    :: * **  * .*    :*::*   **       ** *
            TyrKc
Fh          HTRLEEYCEDERPTYRNMGGTNLFAVTRGLRSPTT------------------------- 697
Dm          QWKLEDFYTSDQSDYKEAQAY--------------------------------------- 517
Ce          QWKLEDLFNLDSSEYKEASINF-------------------------------------- 507
Sm          LNQLESMPTNSTMEEEEEQIDFDITSTTSSSSLLLNNNEIKVINSSESNRSIKHPPNIAY 591
Hs          HQAFETMFHDSSISEVLLHCAN---QTCITL----------------------------- 542
Mm          HQAFETMFHDSSISEEVAEELG---RTASSSSVV-----------------------PYL 569
               :*      .

Fh          ---SVNPVPSSSGGSSNARYFQNMENLQISVSP--------------------------- 727
Dm          ------------------------------------------------------------ 517
Ce          ------------------------------------------------------------ 507
Sm          PRPPLPPRPPPPRRSTSCDYL-IDENDDNQFDMIKEDSKSDLGSDIPVNAMNNNKNNVSH 650
Hs          ------------------------------------------------------------ 542
Mm          PRLPLLPSKTRTLRKQG---E-NKENLDGGLDAAES----LASSSAPAGFIRSTQASS-- 619

Fh          ------------------------------------------------------------ 727
Dm          ------------------------------------------------------------ 517
Ce          ------------------------------------------------------------ 507
Sm          KSHRGIFTRSFKNPLSKQLHIGDLSTDEFGTAEKDLPFTNSGSLGRKRTAPRPPRRTTPV 710
Hs          ------------------------------------------------------------ 542
Mm          --GSPALPRKQRDKS-PSSLLEDAKETCFTRDRKG---GFFSSFMKKRNAPTPPKRSSSF 673

Fh          ------------------------------------------------------------ 727
Dm          ------------------------------------------------------------ 517
Ce          ------------------------------------------------------------ 507
Sm          KPLNFLTDFNLLEDNNCVCPNPYRDDFDHAPLPPPPVHLDSSHHQIHQTRSQ-QHPRPAP 769
```

```
Hs          ------------------------------------------------------------ 542
Mm          REMENQPHKKY-----ELTGLPEQDRMA-MTLP---RNCQRSKLQLERTVSTSSQPEENV 724

Fh          ------------------------------------------------------------ 727
Dm          ------------------------------------------------------------ 517
Ce          ------------------------------------------------------------ 507
Sm          QSNNGSLSNNSNSTPFSVPSVMSSSAIFPSSRQFSYVDPIPLDNSLTRSICPDRLISPLA 829
Hs          ------------------------------------------------------------ 542
Mm          DRANDMLPKKSEE------------GAAPA-RE------RPKAKLLPRG----ATALPLR 761

Fh          ------------------------------------------------------------ 727
Dm          ------------------------------------------------------------ 517
Ce          ------------------------------------------------------------ 507
Sm          PPTPSKHVQLSSKSPALSKSTVNSDLSNKTN-PVVDDCHNKRSMDSNFLNESYSGNNVSS 888
Hs          ------------------------------------------------------------ 542
Mm          APDPAIT---ESDSPGVGVAGVAAAPKGKERNGGTRLGVAGVPEDGEQLGWSSPAKAVAV 818

Fh          ------------------------------------------------------------ 727
Dm          ------------------------------------------------------------ 517
Ce          ------------------------------------------------------------ 507
Sm          KPNVHPTN----ISSSV---------FQE-ELRDRLKQHSQ-QLK-SSS--QQKPITPKT 930
Hs          ------------------------------------------------------------ 542
Mm          LPTTHNHKVPVLISPTLKHTPADVQLIGTDSQGNKFKLLSEHQVTSSGDKDRPRRVKPKC 878

Fh          ------------------------------------------------------------ 727
Dm          ------------------------------------------------------------ 517
Ce          ------------------------------------------------------------ 507
Sm          VPNNSPCN--VTNSVMCNQEKSDSKSSVSNSSPYFTIPRLRPPPRSANPPLISSNNTSGS 988
Hs          ------------------------------------------------------------ 542
Mm          APPPPPVMRLLQHPSTCSDPEEEPT----------------APPAGQHTPETQEGG-KKA 921

Fh          ------------------------------------------------------------ 727
Dm          ------------------------------------------------------------ 517
Ce          ------------------------------------------------------------ 507
Sm          SNNQMSTRGKV-----EPPHCSSELIRSLASTQIKNDNASNSTV-TTTSKLPTNINSNNN 1042
Hs          ------------------------------------------------------------ 542
Mm          APGPMPSSGKPGRPVMPPPQVP-LPTSSISPAKMANGTAGTKVALRKTKQAAEKISAD-- 978

Fh          ------------------------------------------------------------ 727
Dm          ------------------------------------------------------------ 517
Ce          ------------------------------------------------------------ 507
Sm          NSPKPHFKRSENEISDKLSDIIQKRLSWTGPTDDNSAGNQKRSQRLYLPTILSTSSTALD 1102
Hs          ------------------------------------------------------------ 542
Mm          ----KISKEALLECADLLSSAITEPV----P----------------------------- 1001

Fh          ------------------------------------------------------------ 727
Dm          ------------------------------------------------------------ 517
Ce          ------------------------------------------------------------ 507
Sm          ETNGNNHCVPPTKYHLKKHLSNILKELERLIRMNDKKIPGNITSSPSSAAISSSSSTSNL 1162
Hs          ------------------------------------------------------------ 542
Mm          -----------------------------------------------------------N 1002

Fh          ------------------------------------------------------------ 727
Dm          ------------------------------------------------------------ 517
Ce          ------------------------------------------------------------ 507
```

```
Sm      EQLIELADQMEACRLSCSAYIDQATCSARAKFNFRDRFSCLQTFSSLLRSKRLDSICNNN 1222
Hs      ------------------------------------------------------------ 542
Mm      SQLV---DTGHQLLDYCSGYVDSIP-QTRNKFAFREAVSKLELSLQELQVS---STA--- 1052

Fh      ------------------------------------------------------------ 727
Dm      ------------------------------------------------------------ 517
Ce      ------------------------------------------------------------ 507
Sm      TSNLSKYNKNNTNLLKDAENAIKEIINDLDKLTDNVNTTLTLETTLNNSNFPEHKPNSNL 1282
Hs      ------------------------------------------------------------ 542
Mm      -AGVPGTNP----VLNNLLSCVQEI-------SDVVQR---------------------- 1078

Fh      --------------------------    727
Dm      --------------------------    517
Ce      --------------------------    507
Sm      ATSNQSNLTCTENLSITDNRSATVFT    1308
Hs      --------------------------    542
Mm      --------------------------    1078
```

Rac-alpha serine/threonine-protein kinase (Akt1):

```
Ce      ------------------------------------------------------------ 0
Fh      ------------------------------------------------------------ 0
Sm      MEILCNYSQNPVVGSSKSSHTEHEPVSVSAKVQDTTASQRRNINCTSSNIFSSLQNAHLP 60
Hs      ------------------------------------------------------------ 0
Mm      ------------------------------------------------------------ 0
Dm      ------------------------------------------------------------ 0

Ce      -----------------------------MSMT-SLSTKSRRQEDVVIEGWLHKKGEHIR 30
Fh      ------------------------------------------------------------ 0
Sm      VVPNPNVLFKNELTENYSDNLTPRISPYLLGMLQCRPVSLPLTRNVVKEGWLMKRGEHIK 120
Hs      ----------------------------------------MSDVAIVKEGWLHKRGEYIK 20
Mm      ----------------------------------------MNDVAIVKEGWLHKRGEYIK 20
Dm      ----------------MSINTTFDL----SSPSVTSGHALTEQTQVVKEGWLMKRGEHIK 40

Ce      NWRPRYFMIFNDGALLGFRAKPKEGQP--FPEPLNDFMIKDAATMLFEKPRPNMFMVRCL 88
Fh      ------------------------------------------------------------ 0
Sm      NWRRRYFKLREDGTFYGYKIQPKDDMA----QPLNNFTVRDCQIICLNKPKPYTFLIRGL 176
Hs      TWRPRYFLLKNDGTFIGYKERPQDVDQ--REAPLNNFSVAQCQLMKTERPRPNTFIIRCL 78
Mm      TWRPRYFLLKNDGTFIGYKERPQDVDQ--RESPLNNFSVAQCQLMKTERPRPNTFIIRCL 78
Dm      NWRQRYFVLHSDGRLMGYRSKPADSASTPSDFLLNNFTVRGCQIMTVDRPKPFTFIIRGL 100

Ce      QWTTVIERTFYAESAEVRQRWIHAIESISKKYKGTNANPQEELMETNQQPKIDEDSEFAG 148
Fh      ------------------------------------------------------------ 0
Sm      QWTNVVERLFFVETEAERNYWLSAIQSVANRLKSSFEQPVSV---HN------------- 220
Hs      QWTTVIERTFHVETPEEREEWTTAIQTVADGLKKQEEEEMDF---RSGSP---------- 125
Mm      QWTTVIERTFHVETPEEREEWATAIQTVADGLKRQEEETMDF---RSGSP---------- 125
Dm      QWTTVIERTFAVESELERQQWTEAIRNVSSRLIDVGEVAMTP---SEQTDMTDVDM--AT 155

                                                             S_TKc
Ce      AAHAIMGQPSSGHGDNCSIDFRASMISIADTSEAAKRDKITMEDFDFLKVLGKGTFGKVI 208
Fh      -----------------------------------------------MDTLPN-EIQVSI 12
Sm      ------------------LNLAENMI---VDIPQRPVKRYSVNDFRLLKVLGKGTFGKVI 259
Hs      ----------------SDNSGAEEM----EVSLAKPKHRVTMNEFEYLKLLGKGTFGKVI 165
Mm      ----------------SDNSGAEEM----EVSLAKPKHRVTMNEFEYLKLLGKGTFGKVI 165
Dm      ---------------IAEDELSEQFSVQGTTCNSSGVKKVTLENFEFLKVLGKGTFGKVI 200
                                                       :. * :  :   *
                                   S_TKc
```

```
Ce     LCKEKRTQKLYAIKILKKDVIIAREEVAHTLTENRVLQRCKHPFLTELKYSFQTNDRLCF 268
Fh     NILHKRKGTFFGIP------IVSKEEVEHTLTENRVLQQCRHPFMTQLRYSFTTPDRLCF 66
Sm     LCQENETGHFYAMKILKKSVLIEKEEVVHTMTENRVLQQCKHPFMTELRYSFTTPNYLCF 319
Hs     LVKEKATGRYYAMKILKKEVIVAKDEVAHTLTENRVLQNSRHPFLTALKYSFQTHDRLCF 225
Mm     LVKEKATGRYYAMKILKKEVIVAKDEVAHTLTENRVLQNSRHPFLTALKYSFQTHDRLCF 225
Dm     LCREKATAKLYAIKILKKEVIIQKDEVAHTLTESRVLKSTNHPFLISLKYSFQTNDRLCF 260
           .: .   :.:       :: ::** **:**.***:   .***:   *:*** * : ***
                                      S_TKc

Ce     VMEFAIGGDLYYHLNREVQMNKEGFSEPRARFYGSEIVLALGYLHAN-SIVYRDLKLENL 327
Fh     VMEYVNGGELYFHLSRER-----FFPEERTRFYAAEITLALGYLHSQ-NVVYRDLKLENL 120
Sm     VMEYVNGGELFFHLQRDR-----VFSEERAKFYGAEITLALGYLHHQ-NVVYRDLKLENL 373
Hs     VMEYANGGELFFHLSRER-----VFSEDRARFYGAEIVSALDYLHSEKNVVYRDLKLENL 280
Mm     VMEYANGGELFFHLSRER-----VFSEDRARFYGAEIVSALDYLHSEKNVVYRDLKLENL 280
Dm     VMQYVNGGELFWHLSHER-----IFTEDRTRFYGAEIISALGYLHSQ-GIIYRDLKLENL 314
       **::. **:*::**.::        * * *::**.:**   **.*** : .::*********
                                     S_TKc

Ce     LLDKDGHIKIADFGLCKEEISFGDKTSTFCGTPEYLAPEVLDDHDYGRCVDWWGVGVVMY 387
Fh     LLDKDGHIKIADFGLCKEEMYYGAFTKTFCGTPEYLAPEVLLDNDYGRAVDWWGLGVVMY 180
Sm     LLDKDGHIKIADFGLCKEDMYYGASTKTFCGTPEYLAPEVLLDNDYGRSVDWWGLGVVMY 433
Hs     MLDKDGHIKITDFGLCKEGIKDGATMKTFCGTPEYLAPEVLEDNDYGRAVDWWGLGVVMY 340
Mm     MLDKDGHIKITDFGLCKEGIKDGATMKTFCGTPEYLAPEVLEDNDYGRAVDWWGLGVVMY 340
Dm     LLDKDGHIKVADFGLCKEDITYGRTTKTFCGTPEYLAPEVLDDNDYGQAVDWWGTGVVMY 374
       :********::******* :  *    .************** *:***:.***** *****
                                        S_TKc

Ce     EMMCGRLPFYSKDHNKLFELIMAGDLRFPSKLSQEARTLLTGLLVKDPTQRLGGGPEDAL 447
Fh     EMMCGRLPFYSTEHEILFELILQENIEFPSRLSLEAQDILSELLIKDPAKRLGGGPSDAL 240
Sm     EMMCGRLPFYSSDHEVLFELILQENVSFPARLSHPAQDILSRLLIKDPTSRLGGGIQDVL 493
Hs     EMMCGRLPFYNQDHEKLFELILMEEIRFPRTLGPEAKSLLSGLLKKDPKQRLGGGSEDAK 400
Mm     EMMCGRLPFYNQDHEKLFELILMEEIRFPRTLGPEAKSLLSGLLKKDPTQRLGGGSEDAK 400
Dm     EMICGRLPFYNRDHDVLFTLILVEEVKFPRNITDEAKNLLAGLLAKDPKKRLGGGKDDVK 434
       **:*******. :*: ** **:   :: **   :   *: :*: ** *** .***** .*.
          S_TKc                                   S_TK_X

Ce     EICRADFFRTVDWEATYRKEIEPPYKPNVQSETDTSYFDNEFTSQPVQLTPPSRSGALA- 506
Fh     EVMKHHFFFLLDWDRLVRKDILPPWKPDVTSPTDTKYIPEEFQCESIALTPPEKP--VTD 298
Sm     EVMAHLFFASVDWDRLIRKDIQPPWKPDVVDEKDTKYVPDEFKDTSVDLTPPNDNEDNMN 553
Hs     EIMQHRFFAGIVWQHVYEKKLSPPFKPQVTSETDTRYFDEEFTAQMITITPPDQDDS-ME 459
Mm     EIMQHRFFANIVWQDVYEKKLSPPFKPQVTSETDTRYFDEEFTAQMITITPPDQDDS-ME 459
Dm     EIQAHPFFASINWTDLVLKKIPPPFKPQVTSDTDTRYFDKEFTGESVELTPPDPTGP-LG 493
       *:     **   : *      *.: **:**:*  . .** *. .**     :  :***.
             S_TK_X

Ce     TVDEQEEMQSNFTQFSFHNVMGSINRIHEASEDNEDYDMG-      546
Fh     HI----LNGPYFEQFSFHGSRQSLSSHMSGFSFTDQF----      331
Sm     RI----VDGPYFEQFSFHGSRQSLNSRVSGYSFGDTF----      586
Hs     CVDS--ERRPHFPQFSYSASGTA------------------      480
Mm     CVDS--ERRPHFPQFSYSASGTA------------------      480
Dm     SIA----EEPLFPQFSYQGDMASTLGTSSHISTSTSLASMQ      530
        :           * ***:      :
```

Protein kinase C (Pkc):

```
                                           PB1
Fh     ------------------MTVGNEEIPVKYIHNSIAMVTNLPSQADLKAMQSQVMELCQF 42
Sm     ------------------------------------MVTSCPLSANLQVIDQQVRELCSF 24
Ce     -------------MSSPTSLEEDGDIKLKTRFQGQVVVLYARPPLILDDFFALLKDACKQ 47
Dm     ------------------------------------------------------------ 0
Hs     MPTQRDSSTMSHTVAGGGSGDHSHQVRVKAYYRGDIMITHFEPSISFEGLCNEVRDMCSF 60
Mm     MPTQRDSSTMSHTVACGGGGDHSHQVRVKAYYRGDIMITHFEPSISFEGLCSEVRDMCSF 60

                         PB1
Fh     SPDCRFTLKWIDEEQDPCVLSSDLELEEAVRLLHLNKECQLTVLVFDGIPLEPGKPCPGE 102
Sm     TKDQPFTIKWIDEEHDPIVISSEMELKEAFRLHEVNKEWQLTVHVFNGVPSEPGKPCPGE 84
Ce     HKKQDITVKWIDEDGDPISIDSQMELDEAVRCLNSSQEAELNIHVFVGKPELPGLPCQGE 107
```

```
Dm      --------------------------------------MGKLACLFPNVPQAPGLSCDGE 22
Hs      DNEQLFTMKWIDEEGDPCTVSSQLELEEAFRLYELNKDSELLIHVFPCVPERPGMPCPGE 120
Mm      DNEQPFTMKWIDEEGDPCTVSSQLELEEAFRLYELNKDSELLIHVFPCVPERPGMPCPGE 120
                                                    :*   *  **  * **

                                            C1
                                  __________________________
Fh      DLSMYRRGAKRWKKKYYLLHGHKFAARRFNKNAECAYCHGRIWGLGQQGFKCITCRLLLH 162
Sm      DINMYRRGAKRWKKKFYLLHGHQFAARRFNRNAVCAYCKERIWGLGQQGFKCINCRLLLH 144
Ce      DKTVYRRGARRWKK-IYLYNGHRFQAKRLNRRIQCFICHDYIWGIGRQGFRCVDCRLCVH 166
Dm      DRSIYRRGARRWRK-LYRVNGHIFQAKRFNRRAFCAYCQDRIWGLGRQGFKCIQCKLLVH 81
Hs      DKSIYRRGARRWRK-LYCANGHTFQAKRFNRRAHCAICTDRIWGLGRQGYKCINCKLLVH 179
Mm      DKSIYRRGARRWRK-LYCANGHTFQAKRFNRRAHCAICTDRIWGLGRQGYKCINCKLLVH 179
        * .:*****:**:*  *  :** * *:*:*:.  *  *   ***:*:**::*: *:* :*

          C1
        ___________
Fh      RRCRGCVQHKCGEPLTIPGPLNPVTVGRVTPLSPETPNEFAYLNN-------N---ASAN 212
Sm      RRCQGGVRHKCGEAFIRPTYQNMRSISTFSTGS--TPTVWDNNGNRPTTIATNLSSETSN 202
Ce      KKCHRHVRTHCGQALQGPN-IIPMAPASGSLKGAR----------------SN------- 202
Dm      KKCHKLVQKHCTDQPEPLVKERAEE------SSD--PIP----------VPLP------- 116
Hs      KKCHKLVTIECGRHSLPQEPVMPMDQ--SSMHSDHAQTV----------IPYN------- 220
Mm      KKCHKLVTIECGRHSLPPEPMMPMDQ---TMHPDHTQTV----------IPYN------- 219
        ::*:  *  .*

Fh      SRPTSYQLSHAGV-ETPNHNSGASRLEIAYAQQSTFEEERNRPRTGIQNP-------AFV 264
Sm      TLPPRLPVTIQSYKDEPRPPKQEKIIALSERESNQTSDKHITPTSDIKNNLKPGSDNLII 262
Ce      ------TSSSTTR------------SGGG-IDNGAFHEHE------IE------------ 225
Dm      ------PLPYEAM------------SGGA-EACETHDHAH------IV------------ 139
Hs      ------PSSHESL------------DQVG-EEKEA------------------------- 236
Mm      ------PSSHESL------------DQVG-EEKEA------------------------- 235
                                    .

                                                           S_TKc
                                                    _________
Fh      DRR------KPRCTEISVPRPDVVIPILEERIAEMSVITTTSSSGQVGLQDFKLLKVIGR 318
Sm      ERSGVHPSSVPVPVSIIDKVDDKSIPIIEQRIIDIPDI--PITSGRVGLHDFNLLKVIGR 320
Ce      ---------SPGST---------------SHDASRAMNGNGSSKWAVSLNDFRLLTVIGR 261
Dm      ---------APPP---------------------PEDPLEPGTQRQYSLNDFELIRVIGR 169
Hs      ----------------------------------MNTRESGKASSSLGLQDFDLLRVIGR 262
Mm      ----------------------------------MNTRESGKASSSLGLQDFDLLRVIGR 261
                                                  :.   .*:** *: ****
                                    S_TKc
        ____________________________________________________________
Fh      GSYAKVFQVEHIPTQRIYAMKVIKKETILDEEDIEWVQTEKHVFECATNHPFLVGLHSCF 378
Sm      GSYAKVFQVEHKPTNRIYAMKVIKKETILDEEDIDWVQTEKHVFECATNHPFLVGLHSCF 380
Ce      GSYAKVVQAEHVSTRQIYAIKIIKKEMFNEDEDIDWVQTEKSVFEAASNYPFLVGLHSCF 321
Dm      GSYAKVLMVELRRTRRIYAMKVIKKALVTDDEDIDWVQTEKHVFETASNHPFLVGLHSCF 229
Hs      GSYAKVLLVRLKKTDRIYAMKVVKKELVNDDEDIDWVQTEKHVFEQASNHPFLVGLHSCF 322
Mm      GSYAKVLLVRLKKTDRIYAMKVVKKELVNDDEDIDWVQTEKHVFEQASNHPFLVGLHSCF 321
        ******. ..   * :***:*::**  . ::***:****** *** *:*:**********
                                    S_TKc
        ____________________________________________________________
Fh      QTDSRLFFVIEFVNGGDLMFYMQRNLRLAEDYARFYSAEICLALNFLHERGIIYRDLKLD 438
Sm      QTRSRLFFVIEFVNGGDLMFYMQRNLRLAEDYARFYAAEICIALNFLHERGIIYRDLKLD 440
Ce      QTESRLFFVIEFVPGGDLMFHMQQQRKLPEEHARFYSGEIILALHFLHSRGIIYRDLKLD 381
Dm      QTPSRLFFVIEFVRGGDLMYHMQRQRRLPEEHARFYAAEISLALNFLHEKGIIYRDLKLD 289
Hs      QTESRLFFVIEYVNGGDLMFHMQRQRKLPEEHARFYSAEISLALNYLHERGIIYRDLKLD 382
Mm      QTESRLFFVIEYVNGGDLMFHMQRQRKLPEEHARFYSAEISLALNYLHERGIIYRDLKLD 381
        ** ********:* *****::**:: :* *::****:.** :**::**.:**********
                                   S_TKc
        ____________________________________________________________
Fh      NVLMDSEGHIKLTDYGMCKEGITADVMTSTFCGTPNYIAPEILRGENYNFSVDWWALGVL 498
Sm      NVLMDSEGHIKLTDYGMCKEGIIGDMTTTTFCGTPNYIAPEILKGESYSFSVDWWALGVL 500
Ce      NVLIDAEGHIKLTDYGMCKENIKDGDLTSTFCGTPNYIAPEILRGDEYGFSVDWWALGVL 441
Dm      NVLLDHEGHIKLTDYGMCKEGIRPGDTTSTFCGTPNYIAPEILRGEDYGFSVDWWALGVL 349
Hs      NVLLDSEGHIKLTDYGMCKEGLRPGDTTSTFCGTPNYIAPEILRGEDYGFSVDWWALGVL 442
Mm      NVLLDSEGHIKLTDYGMCKEGLRPGDTTSTFCGTPNYIAPEILRGEDYGFSVDWWALGVL 441
        ***:* **************.:  .  *:**************:*:.*.***********
                                   S_TKc
        ____________________________________________________________
Fh      MFEMLAGRSPWENVGQSVNQDQNSEDYLFQVILTRPIRFPRSISVRATNILRAFLTKNPA 558
Sm      MFEMLAGRSPWEGVGQSANPDQNTEDYLFQIILSRPIRFPRSISVRATSILNAFLQKVPT 560
```

```
Ce      MFEMMAGRSPFDIVGMQ-NSEENTEDYLFQIILERQIRIPRSLSVRASGILKGFLNKDPT 500
Dm      LYEMLAGRSPFDLAGASENPDQNTEDYLFQVILEKTIRIPRSLSVRAASVLKGFLNKNPA 409
Hs      MFEMMAGRSPFDIVGSSDNPDQNTEDYLFQVILEKQIRIPRSLSVKAASVLKSFLNKDPK 502
Mm      MFEMMAGRSPFDIVGSSDNPDQNTEDYLFQVILEKQIRIPRSLSVKAASVLKSFLNKDPK 501
        ::**:*****::  .*  .  *  ::*:******:**  :  **:***:**:*:.:*..**  *  *
                  S_TKc                              S_TK_X

Fh      ERLGCLPD--SNFADIMNHSFFRP-VDWVALEQKQIAPPYRPTYANDYDLTHFDPTFTDE 615
Sm      ERLGCAPN--SSFGDIMEHGFFKP-IDWVALERKEVHPPYRPTCGGDRDLIHFDPAFTDE 617
Ce      ERLGCKLDINEGLRDMKEHQFFRGFIDWEALEQKAVAPPYHPAVESDRDLTHFDHQFTDE 560
Dm      DRLGCHRE--SAFMDIVSHPFFKN-MDWELLERKQVTPPFKPRLDSDRDLANFPPEFTGE 466
Hs      ERLGCHPQ--TGFADIQGHPFFRN-VDWDMMEQKQVVPPFKPNISGEFGLDNFDSQFTNE 559
Mm      ERLGCHPQ--TGFADIQGHPFFRN-VDWDMMEQKQVVPPFKPNISGEFGLDNFDSQFTNE 558
        :****  :     : *:  *  **:   :**   :*:*  :  **::*     .:  .*  :*     **.*
                  S_TK_X

Fh      PVVFTPDNEEVVSKIDQTEFEGFEYVNPLLMTLEEPV 652
Sm      PVVLTPDNEAVMSRMDQTEFDGFEYVNPLLMSLDEQV 654
Ce      LPQLSPDNPDVIARIDQSEFDGFEYVNPLQMSREDSV 597
Dm      AVQLTPDDDHVIDNIDQSEFEGFEYVNPLLMSLEDCV 503
Hs      PVQLTPDDDDIVRKIDQSEFEGFEYINPLLMSAEECV 596
Mm      PVQLTPDDDDIVRKIDQSEFEGFEYINPLLMSAEECV 595
           ::**:  ::  .:**:**:****:***  *:  ::  *
```

Polo-like kinase 1 (Plk1):

```
                                                            S_TKc
Ce      -----------------MQRVQPSAARVKSQKKEKAPPDVPDVILDGERKTRYEKGKFLG 43
Fh      ----------------------------MASKDAAKNKDPPEIIIDAKNKRKYTRGRFLG 32
Sm      ----------------------------MAANDSAKPKDPPEVIVDVTRKREFLRGRFLG 32
Dm      ----------------------------MAAKPEDKSTDIPDRLVDINQRKTYKRMRFFG 32
Hs      MSAAVTAGKLARAPADPGKAGVPGVAAPGAPAAAPPAKEIPEVLVDPRSRRRYVRGRFLG 60
Mm      MNAAAKAGKLARAPADLGKGGVPGDAVPGAPVAAPLAKEIPEVLVDPRSRRQYVRGRFLG 60
                                   :         :  *:  ::*    :   :  :  :*:*
                                 S_TKc

Ce      KGGFAHCYELRNKSTGELFAGKVVPKALLIKQYQRDKMAQEVQIHRNLQHRNVVKLYHFF 103
Fh      KGGFAKCYELVDQETNETFAGKVVPKAALTKASQKEKMQQEINIHRTLRHDNVVGFHGYF 92
Sm      KGGFAKCYELVDKKTGEKYAGKVINKSALVKESQKEKMRQEISIHRSLKHENVVRFYGNF 92
Dm      KGGFAKCYEIIDVETDDVFAGKIVSKKLMIKHNQKEKTAQEITIHRSLNHPNIVKFHNYF 92
Hs      KGGFAKCFEISDADTKEVFAGKIVPKSLLLKPHQREKMSMEISIHRSLAHQHVVGFHGFF 120
Mm      KGGFAKCFEISDADTKEVFAGKIVPKSLLLKPHQKEKMSMEISIHRSLAHQHVVGFHDFF 120
        *****:*:*:  :  .*  :  :***::  *   :  *   *::*    *:  ***.*  *  ::*  ::   *
                                 S_TKc

Ce      EDKSNVYITLELCPRRSLMELHKRRKAVTEPEARYFTYQIVEGVLYLHNLKIVHRDLKLG 163
Fh      EDRDFIYIVLELCKRRSLLELHKRRRFISEPEARYFLRQIVHGCQYLHKNKIMHRDLKLA 152
Sm      EDSEFIYILLELCNRRSLLELHKRRKYVTEPEARYFMKQIVHGCQYLHQNKVMHRDLKLA 152
Dm      EDSQNIYIVLELCKKRSMMELHKRRKSITEFECRYYIYQIIQGVKYLHDNRIIHRDLKLG 152
Hs      EDNDFVFVVLELCRRRSLLELHKRRKALTEPEARYYLRQIVLGCQYLHRNRVIHRDLKLG 180
Mm      EDSDFVFVVLELCRRRSLLELHKRRKALTEPEARYYLRQIVLGCQYLHRNQVIHRDLKLG 180
        **  .  :::  ****  :**::******:  ::*  *.**:    **:  *    ***    ::******.
                                S_TKc

Ce      NLFLNDELQVKIGDFGLATTCD-NDERKKTLCGTPNYIAPEVLNKIGHSFEVDLWAIGCI 222
Fh      NLFLSDELIIKIGDFGLASRIVHEGEKKKTLCGTPNYIAPEVLTKGGHSFEVDCWSLGCI 212
Sm      NLFLDDNLKIKIGDFGLASRISHEGEKKKTLCGTPNYIAPEILSKGGHSFEVDSWSLGCI 212
Dm      NLFLNDLLHVKIGDFGLATRIEYEGERKKTLCGTPNYIAPEILTKKGHSFEVDIWSIGCV 212
Hs      NLFLNEDLEVKIGDFGLATKVEYDGERKKTLCGTPNYIAPEVLSKKGHSFEVDVWSIGCI 240
Mm      NLFLNEDLEVKIGDFGLATKVEYEGERKKTLCGTPNYIAPEVLSKKGHSFEVDVWSIGCI 240
        ****.:  *  :********:      :.*:**************:*.*  *******  *::**:
                               S_TKc

Ce      LYILLFGHPPFESKSLEETYSRIKNNNYVIPTSASAAASQLIRVLLDPVPSRRPNARAVC 282
Fh      LYTLLVGKPPFETSELEKTYTKIRHNEYQIPTRVSTSASKLIRALLHADPSKRPSMFTIL 272
Sm      LYTLLVGSPPFETTKLEETYARIKQNEYRIPIRVSTNASMLIRSLLHADPERRPNMFNVL 272
Dm      MYTLLVGQPPFETKTLKDTYSKIKKCEYRVPSYLRKPAADMVIAMLQPNPESRPAIGQLL 272
Hs      MYTLLVGKPPFETSCLKETYLRIKKNEYSIPKHINPVAASLIQKMLQTDPTARPTINELL 300
Mm      MYTLLVGKPPFETSCLKETYLRIKKNEYSIPKHINPVAASLIQKMLQTDPTARPTIHELL 300
```

```
        :* **.* ****:. *:.** :*:: :* :*      *: ::  :*.  *  **    :
        S_TKc
Ce      RDHFFKSGFMPARLPVSCLTMVPHLNDDEYAEENVS--PSGTIDQRGPHQA-GRSGLSAI 339
Fh      DDEFFK-DYTPNCLPVTALTTCPRFDVT--I--QSGRRPLSDINPLEDLPITSGGGTVQK 327
Sm      DHDFFK-DFTPNGLPVSCLSTCPRFDTMHRP--QTGRRPLSDINPVEDVPITSGGGALGN 329
Dm      NFEFLKGSKVPMFLPSSCLTMAPRIGSNDTIEDSMHRKPLMEMNGIRPD----DTRLEST 328
Hs      NDEFFTSGYIPARLPITCLTIPPRFSIAPSSLDPSNRKPLTVLNKGLENPLPERPREKEE 360
Mm      NDEFFTSGYIPARLPITCLTIPPRFSIAPSSLDPSSRKPLKVLNKGVENPLPDRPREKEE 360
          .*:. .  *  ** :.*:  *::.            *   ::

Ce      PAHLVSRNSERQQTHRMEAYRQPTDCYLSNLLAQVN------------DLLATPTADIDD 387
Fh      PS-----------AAPVD--AEPDDCWLLTLKEEVCLSLCLSNSVQIQTLLKEAEVLESN 374
Sm      VG-----------GAAVDKAVQPDDCWLGLLKHKLG------------RVLEEPKEFDDS 366
Dm      FLKA-NLHDAITASAQVCRHSEDYRSDIESLYQQLT------------NLI-N-GKPRIL 373
Hs      --------------PVVRETGEVVDCHLSDMLQQLH------------SVN-A-SKPSER 392
Mm      --------------PVVRETNEAIECHLSDLLQQLT------------SVN-A-SKPSER 392
                        :    :   . :  :  ::              :
                                         POLO_box
Ce      AEAALDSYQSPEALPVFWISKWVDYSDKYGIGYQLCDNSVGVLFNDNSRIMLDTAGTQLT 447
Fh      TLLAMEESEHPASCPVYWVSKWVDYSDKYGLGYQLCDNSSGVVFNDVTRLLLAANLQNMQ 434
Sm      SPKAMEESEVPAACPIYWVSKWVDYSDKYGLGYQLCDNSYGVVFNDVTRLLLTTNEQNLQ 426
Dm      QGNLGDENTDPAAQPLFWISKWVDYSDKYGFGYQLCDEGIGVMFNDTTKLILLPNQINVH 433
Hs      GLVRQEEAEDPACIPIFWVSKWVDYSDKYGLGYQLCDNSVGVLFNDSTRLILYNDGDSLQ 452
Mm      GLVRQEEAEDPACIPIFWVSKWVDYSDKYGLGYQLCDNSVGVLFNDSTRLILYNDGDSLQ 452
             :.   * . *::*:***********:******:. **:*** ::::*     .:
                POLO_box                POLO_box
Ce      YIEKTEKEHYFDMESAIPSGLQKKMTLLKYFRSYMNDHLLQAGQQVTRKVGDDLARLPTL 507
Fh      YIEANGKEHFYTKS-DHPESLMKKITLLNYFKQYMQENLL-------------------- 473
Sm      YIDEHGTERLFTLS-KHPEYLSKKVTLLTCFKAYMHQNLLKAGENIARPDTDAMTRLPFL 485
Dm      FIDKDGKETYMTTT-DYCKSLDKKMKLLSYFKRYMIEHLVKAGANNVNIESDQISRMPHL 492
Hs      YIERDGTESYLTVS-SHPNSLMKKITLLKYFRNYMSEHLLKAGANITPREGDELARLPYL 511
Mm      YIERDGTESYLTVS-SHPNSLMKKITLLNYFRNYMSEHLLKAGANITPREGDELARLPYL 511
        :*:   .*          . * **:.**. *: ** ::*:
                                      POLO_box
Ce      RVWFRTKSAIVLHLSNGTVQINFFNDHIKMVLCPLMQAVTFIDENKRMFTYKFSHLAENG 567
Fh      -------------------KINFFEDHAKIILCPLMRAVTYIDSNREFKTYQFKLLSKGG 514
Sm      RRWFRTRSAIVLHLSNGTLQINFFDDHAKIILCPLMRAVTYIDANREFRTYRFKHLREFG 545
Dm      HSWFRTTCAVVMHLTNGSVQLNF-SDHMKLILCPRMSAITYMDQEKNFRTYRFSTIVENG 551
Hs      RTWFRTRSAIILHLSNGSVQINFFQDHTKLILCPLMAAVTYIDEKRDFRTYRLSLLEEYG 571
Mm      RTWFRTRSAIILHLSNGTVQINFFQDHTKLILCPLMAAVTYINEKRDFQTYRLSLLEEYG 571
                           ::** .** *::*** * *:*::: :: : **::. : : *
          POLO_box
Ce      CPEKFLHRIQYAKCMIQRLVEEHTKEETKHNAPAANAV-----RLPSTSSNVRLESAADI 622
Fh      ITQELVNRLNYAIVMIDTLLSPSQNSCPSK----KSAPPDSKSAVAPVSSAVRQATAAGS 570
Sm      LSKDLANRLRYAQEMIERLIPKCGTTSSNVNDPNTKMTVDNGKSNKPVSADVQKTNATGT 605
Dm      VSKDLYQKIRYAQEKLRKMLEKMFT----------------------------------- 576
Hs      CCKELASRLRYARTMVDKLLSSRSASNRLKAS---------------------------- 603
Mm      CCKELASRLRYARTMVDKLLSSRSASNRLKAS---------------------------- 603
          :.:  ::.**   :  ::

Ce      QPAYPSSSRR-----        632
Fh      AAAAKAIARLTKPEN        585
Sm      GAATKKITRPTNQ--        618
Dm      ---------------        576
Hs      ---------------        603
Mm      ---------------        603
```
